# Stomatal responses of terrestrial plants to global change

**DOI:** 10.1038/s41467-023-37934-7

**Published:** 2023-04-17

**Authors:** Xingyun Liang, Defu Wang, Qing Ye, Jinmeng Zhang, Mengyun Liu, Hui Liu, Kailiang Yu, Yujie Wang, Enqing Hou, Buqing Zhong, Long Xu, Tong Lv, Shouzhang Peng, Haibo Lu, Pierre Sicard, Alessandro Anav, David S. Ellsworth

**Affiliations:** 1 Guangdong Provincial Key Laboratory of Applied Botany, South China Botanical Garden, Chinese Academy of Sciences, Xingke Road 723, Guangzhou, 510650 China; 2 College of Life Sciences, Gannan Normal University, Ganzhou, 341000 China; 3 School of Geographical Sciences, Jiangsu Second Normal University, Nanjing, 211200 China; 4 Guangdong Provincial Key Laboratory of Silviculture, Protection and Utilization, Guangdong Academy of Forestry, Guangzhou, Guangdong, 510520 China; 5 Key Laboratory of Vegetation Restoration and Management of Degraded Ecosystems, South China Botanical Garden, Chinese Academy of Sciences, Guangzhou, 510650 China; 6 Department of Ecology & Evolutionary Biology, Princeton University, Princeton, NJ USA; 7 High Meadows Environmental Institute, Princeton University, Princeton, NJ USA; 8 Division of Geological and Planetary Sciences, California Institute of Technology, Pasadena, California, 91125 USA; 9 State Key Laboratory of Soil Erosion and Dryland Farming on the Loess Plateau, Northwest A&F University, Yangling, 712100 China; 10 Department of Geography, Faculty of Arts and Sciences, Beijing Normal University, Zhuhai, 519087 China; 11 ARGANS Ltd, 260 route du Pin Montard, 06410 Biot, France; 12 ENEA, Climate Modeling Laboratory, CR Casaccia, 301 Via Anguillarese, 00123 Rome, Italy; 13 Hawkesbury Institute for the Environment, Western Sydney University, Penrith, NSW Australia

**Keywords:** Plant ecology, Climate-change ecology

## Abstract

Quantifying the stomatal responses of plants to global change factors is crucial for modeling terrestrial carbon and water cycles. Here we synthesize worldwide experimental data to show that stomatal conductance (*g*_s_) decreases with elevated carbon dioxide (CO_2_), warming, decreased precipitation, and tropospheric ozone pollution, but increases with increased precipitation and nitrogen (N) deposition. These responses vary with treatment magnitude, plant attributes (ambient *g*_s_, vegetation biomes, and plant functional types), and climate. All two-factor combinations (except warming + N deposition) significantly reduce *g*_s_, and their individual effects are commonly additive but tend to be antagonistic as the effect sizes increased. We further show that rising CO_2_ and warming would dominate the future change of plant *g*_s_ across biomes. The results of our meta-analysis provide a foundation for understanding and predicting plant *g*_s_ across biomes and guiding manipulative experiment designs in a real world where global change factors do not occur in isolation.

## Abstract (web summary)

Stomatal conductance is an important plant ecophysiological trait and a common parameter in earth system models. This global meta-analysis shows how CO_2_, warming and other global change factors affect stomatal conductance individually and interactively.

## Introduction

Stomata are small pores bounded by a pair of guard cells on plant leaf surfaces that regulate carbon uptake and water loss of terrestrial plants^1^. Stomatal conductance (*g*_s_), determined by stomatal pore aperture and density, has long been a fundamental parameter of earth system models^2^. As *g*_s_ is sensitive to various environmental change factors, its dynamics are essential for understanding and modeling global carbon and water cycles in a changing climate^3,4^.

**Fig. 1:**
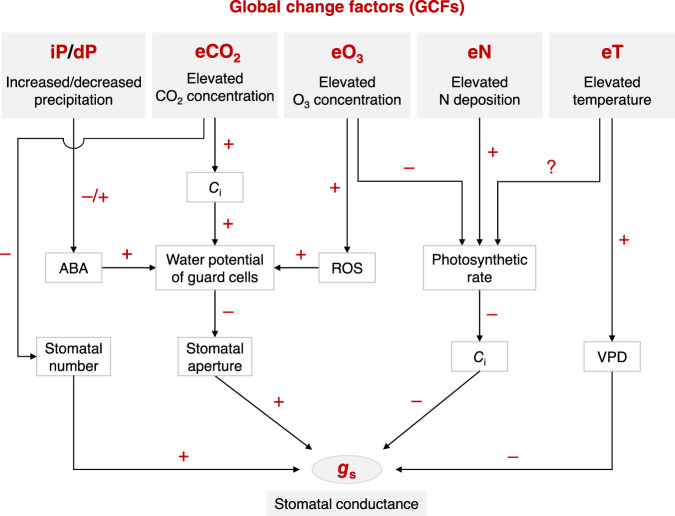
A conceptual diagram depicting the physiological mechanisms underlying the effects of global change factors on stomatal conductance (*g*_s_). +, –, and ? indicate positive, negative, and uncertain effects, respectively. *C*_i_: intercellular CO_2_ concentration, ABA: abscisic acid, ROS: reactive oxygen species, VPD: vapor pressure deficit.

Despite our knowledge about how stomata are regulated by global change factors (GCFs) such as elevated carbon dioxide (CO_2_)^5–9^, warming^10^, change in precipitation^11^, enhanced nitrogen (N) deposition^12^ and surface ozone (O_3_) pollution^13,14^ (Fig. 1), our ability to predict *g*_s_ in the future is still limited due to three main concerns. First, stomatal sensitivities to different GCFs (e.g., the percentage change of *g*_s_ per 100 ppm CO_2_ increase) remain largely unknown. This is in part due to the fact that the climate forcing used in many manipulation experiments (particularly in the precipitation and nitrogen deposition experiments) exceeded the Intergovernmental Panel on Climate Change (IPCC) predicted ranges^15^, thus suggesting that the stomatal sensitivities normalized to the forcing factor, rather than the overall magnitude of changes, provide more meaningful information for models^16^. Second, the interaction of these *g*_s_ sensitivities with climate and plant attributes remains poorly understood, limiting our predictive capacity to broad groups of plants having common features (i.e., biomes or plant functional types). Although *g*_s_ has been related to natural climate gradients^3^, this does not mean that space-for-time substitution can inform how *g*_s_ will change in the future. Third, there is a paucity of evidence for the *g*_s_ response to interactions between GCFs, despite their importance for predicting *g*_s_ in the real world where multiple GCFs are simultaneously in play (e.g., warming × elevated CO_2_ concentration)^17^. Empirical data from manipulative experiments will better inform land surface models, such as the Community Atmosphere Biosphere Land Exchange (CABLE) model^18^, if these limitations are resolved. Current land surface models commonly predict *g*_s_ from net leaf photosynthesis using the Ball-Berry model, which has considered the effects of atmospheric CO_2_ concentration but neither the interactions between GCFs nor the differential responses across plant functional types or biomes^2^.

Here we synthesized experimental data from 616 published papers to examine the responses of *g*_s_ to different GCFs, including elevated CO_2_ concentration (eCO_2_), elevated temperature (eT), increased/decreased precipitation (iP/dP), elevated nitrogen deposition (eN), and elevated O_3_ concentration (eO_3_), both individually and in combination. Unlike most previous meta-analyzes^7,19–26^, we focused on the stomatal sensitivity to various GCFs in addition to the overall magnitude of change. Then, we examined whether the responses of *g*_s_ to the two-factor combinations were additive, synergistic, or antagonistic. We ultimately predicted the changes in *g*_s_ by the end of this century across biomes under different scenarios of greenhouse gas emissions^15^, based on the stomatal sensitivities to different GCFs. We found that the GCFs’ effects were commonly additive but became antagonistic as their effect sizes increased. Furthermore, our analysis suggests that rising CO_2_ and warming will likely have dominant impacts on the future change in plant gs across biomes.

## Results

### Overview of the global change experiments

Our dataset included 5352 pairs of treatment versus control observations for 444 species across different vegetation biomes (Supplementary Data 1). The manipulative experiments were mainly conducted in temperate biomes of the Northern Hemisphere in the United States, Europe, and China. The experiments were less common in tropical and subtropical forests (Fig. S1a). The treatment magnitudes of eCO_2_ and outdoor eT experiments were within the change ranges projected under all the RCPs (Fig. S1b, c). In contrast, the magnitudes of iP/dP, eN, and eO_3_ were generally greater than the projected ranges from IPCC predictions (Fig. S1d–g).

### Stomatal responses to single global change factors

**Fig. 2:**
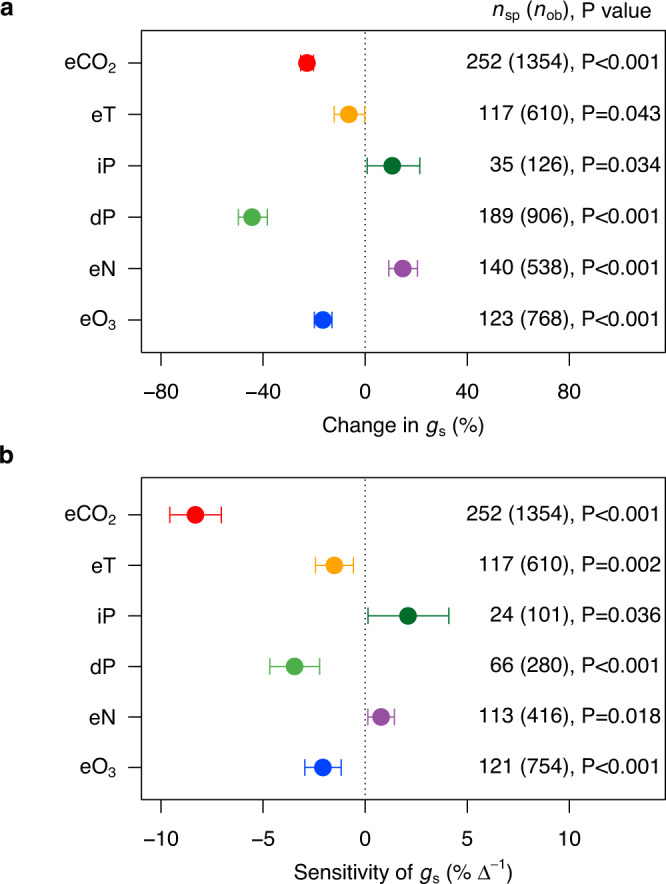
Responses of *g*_s_ to global change factors (GCFs). **a** The overall changes of *g*_s_ in response to GCFs. **b** The *g*_s_ sensitivities in response to GCFs. $\triangle$ in (**b**) represents one unit of eCO_2_ (100 ppm increase), eT (1 °C increase), iP/dP (10% change), eN (1 g m^–2^ yr^–1^ increase), or eO_3_ (10 ppb increase). The weighted mean values are reported with error bars indicating the 95% confidence interval. All the responses are significantly different from zero at *P* < 0.05. The numbers outside and inside parentheses represent the number of species (*n*_sp_) and observations (*n*_ob_), respectively. See Fig. 1 for variable abbreviations.

Stomatal conductance was reduced significantly by eCO_2_, eT, dP, and eO_3_, changing on average by –8.3% per 100 ppm CO_2_ increase, –1.5% per 1 °C temperature increase, –3.5% per 10% precipitation decrease, and –2.1% per 10 ppb O_3_ increase (Fig. 2). By contrast, *g*_s_ was enhanced significantly by iP and eN, with sensitivities of +2.1% per 10% precipitation increase and +0.8% per 1 g m^–2^ year^–1^ nitrogen increase (Fig. 2).

**Fig. 3:**
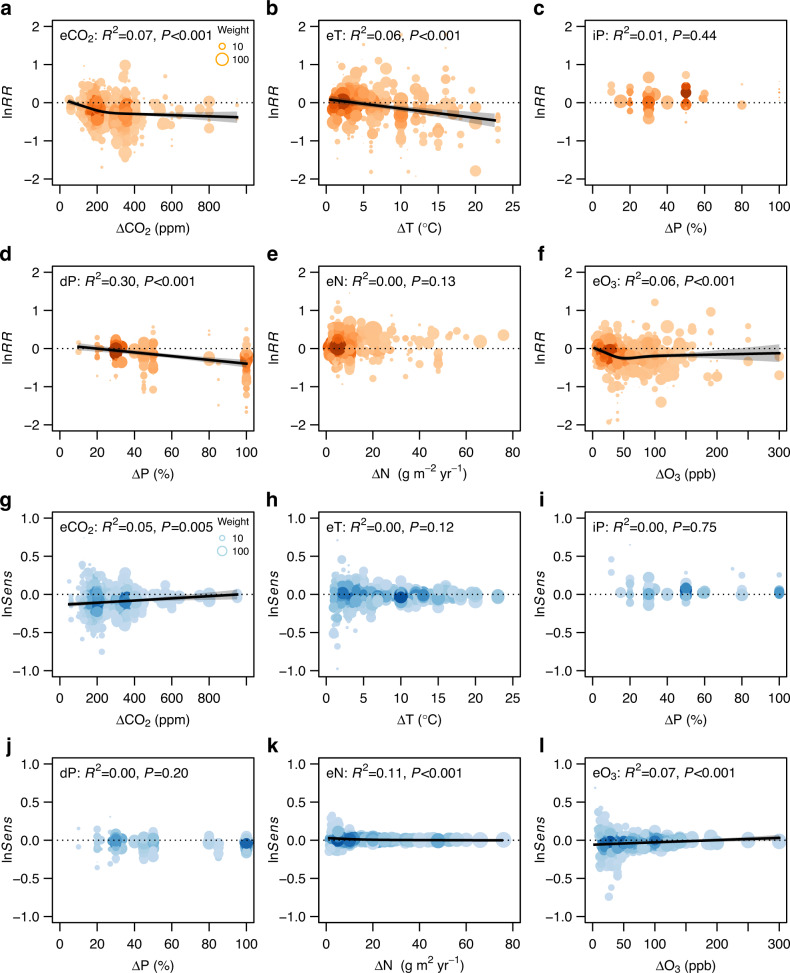
Stomatal responses to global change factors in relation to treatment magnitude. **a**–**f** Natural log-transformed response ratio (ln*RR*) of *g*_s_ to GCFs. **g**–**l** Natural log-transformed sensitivity (ln*Sens*) of *g*_s_ to GCFs. The size of each point represents the adjusted weight of each data point, and the darker the color means the higher the point density. The error bands surrounding the regression lines represent the 95% confidence interval. See Fig. 1 for variable abbreviations.

**Fig. 4:**
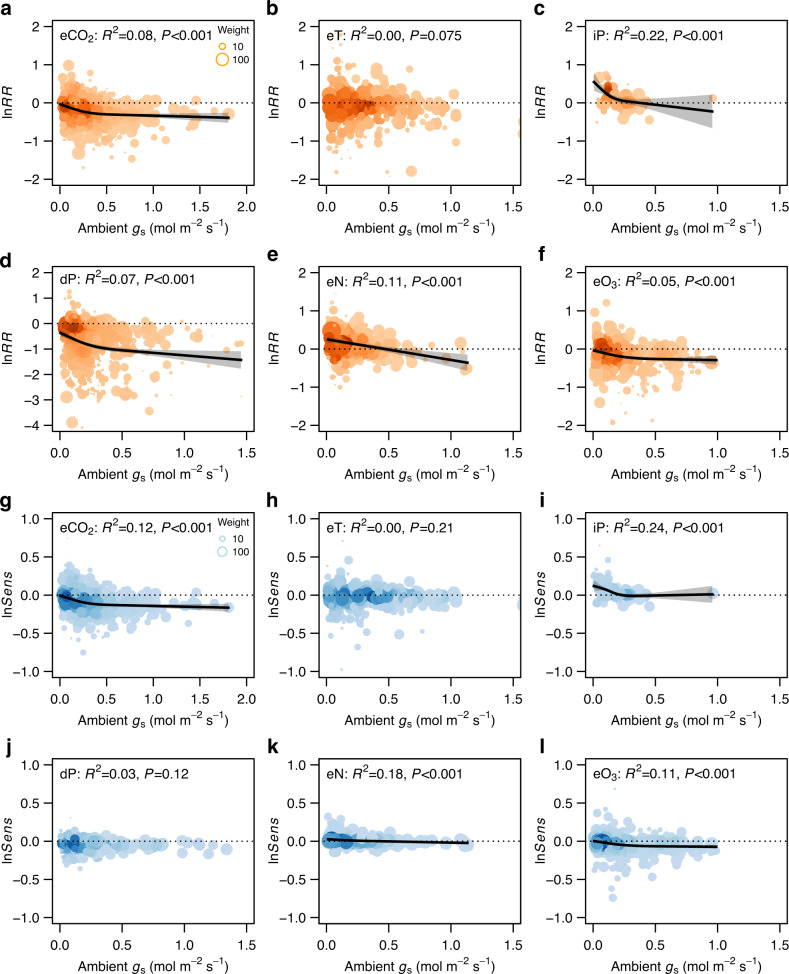
Stomatal responses to global change factors in relation to plant ambient *g*_s_. **a**–**f** Natural log-transformed response ratio (ln*RR*) of *g*_s_ to GCFs. **g**–**l** Natural log-transformed sensitivity (ln*Sens*) of *g*_s_ to GCFs. The size of each point represents the adjusted weight of each data point, and the darker the color means the higher the point density. The error bands surrounding the regression lines represent the 95% confidence interval. See Fig. 1 for variable abbreviations.

The overall magnitudes of changes were significantly greater for the indoor CO_2_, dP, and eN experiments than for the outdoor ones (Fig. S2a), but the stomatal sensitivities did not significantly differ between the indoor and outdoor experiments (Fig. S2b). The overall change magnitudes increased significantly with the treatment magnitudes for eCO_2_, eT, dP, and eO_3_ experiments (Fig. 3a, b, d, f). The reductions of *g*_s_ peaked at ΔCO_2_ of *ca*. 300 ppm (Fig. 3a) or ΔO_3_ of 50 ppb (Fig. 3f), and the stomatal sensitivity decreased significantly with the treatment magnitude of CO_2_, N, and O_3_ (Fig. 3g, k, l). The *g*_s_ response ratio did vary significantly with experimental duration under eCO_2_ (Fig. S3a) because the short-term eCO_2_ experiments commonly exerted greater treatment magnitudes than the longer-term ones. However, the *g*_s_ sensitivity did not vary significantly with eCO2 experimental duration (Fig. S3g), and the *g*_s_ responses to other GCFs did not vary with experimental duration (Fig. S3b–f, h–1). The response ratios of *g*_s_ changed significantly with ambient *g*_s_ under all GCFs except eT (Fig. 4a–f). The *g*_s_ sensitivities to eCO_2_ and eO_3_ were significantly higher for greater ambient *g*_s_ (Fig. 4g, l), and the sensitivities to iP and eN were higher for smaller ambient *g*_s_ (Fig. 4i, k). The *g*_s_ responses increased significantly with mean annual temperature under eCO_2_ but no other GCFs (Fig. S4). The *g*_s_ responses to eCO_2_, eT, and dP did not change with aridity index (AI; Fig. S5a, b, d, g, h, j), both the response ratio and sensitivity to iP decreased with increasing AI (Fig. S5c, i), and the response ratios varied significantly but the sensitivities did not vary with AI under eN and eO_3_ (Fig. S5e, f, k, 1). However, vapor pressure difference (VPD) did not affect the stomatal response ratio or sensitivity to any of the global change factors (Fig. S6).

The stomatal sensitivities differed significantly among vegetation biomes (Table 1) and plant functional types (Table S1). Most biomes showed significant sensitivity to eCO_2_, except boreal forest, which only showed significant sensitivity to eT, and Mediterranean woodland, which was not sensitive to any of the tested GCFs (Table 1). Only desert plants showed significant sensitivity to iP (Table 1). Conifers showed a lower sensitivity to eCO_2_ than broad-leaved trees, grasses and forbs (Table S1).

**Table 1:** Global environmental change sensitivity of stomatal conductance across biomes

Biome	eCO_2_	eT	iP	dP	eN	eO_3_
Boreal forest	–2.9 [–8.7, 3.2]	**–26.5 [–35.7, –16.1]**	/	/	0.2 [–3.1, 3.7]	–1.3 [–4.1, 1.7]
	*5(16)*,*P* = 0.34	*4(18),P* < *0.001*			*5(10)*,*P* = 0.88	*6(54)*,*P* = 0.40
Temperate forest	**–6.9 [–10.1, –3.6]**	–0.5 [–7.2, 6.6]	3.7 [–0.5, 8.0]	**–3.7 [–5.6, –1.7]**	0.7 [–1.1, 2.5]	**–2.2 [–3.9, −0.5]**
	*43(261),P* < *0.001*	*14(39)*,*P* = 0.89	*7(12)*,*P* = 0.083	*19(97),P* < *0.001*	*24(77)*,*P* = 0.51	*58(398),P* = *0.011*
Subtropical forest	**–8.1 [–11.6, –4.5]**	/	–3.2 [–6.8, 0.5]	–1.9 [–4.2, 0.4]	**1.0 [0.1, 2.0]**	**–1.9 [–3.8, –0.1]**
	*17(95),P* < *0.001*		*4(12)*,*P* = 0.10	*8(55)*,*P* = 0.11	*22(113),P* = *0.031*	*27(99),P* = *0.041*
Tropical forest	/	/	/	**–6.3 [–10.0, –2.4]**	/	/
				*15(15),P* = *0.002*		
Temperate grassland	**–9.6 [–13.7, –5.7]**	**–7.7 [–13.8, –1.1]**	2.7 [–4.1, 10.0]	**–3.5 [–4.8, –2.1]**	0.6 [–0.4, 1.5]	/
	*47(113),P* < *0.001*	*7(39),P* = *0.023*	*6(7)*,*P* = 0.45	*19(72),P* < *0.001*	*23(76)*,*P* = 0.20	
Mediterranean woodland	–1.5 [–8.1, 5.6]	3.6 [–2.9, 10.6]	/	–0.3 [–3.3, 2.8]	0.7 [–2.4, 4.0]	–3.8 [–9.8, 2.7]
	*6(23)*,*P* = 0.67	*4(37)*,*P* = 0.29		*9(58)*,*P* = 0.84	*10(29)*,*P* = 0.61	*8(13)*,*P* = 0.24
Desert	**–9.8 [–13.7, –5.7]**	/	**3.3 [0.8, 5.8]**	/	/	/
	*7(127),P* < *0.001*		*10(68),P* = *0.009*			
Cropland	**–11.8 [–14.3, –9.2]**	**–5.4 [–10.2, –0.2]**	/	/	0.3 [–0.9, 1.5]	–1.5 [–5.0, 2.2]
	*22(266),P* < *0.001*	*6(47),P* = *0.041*			*3(33)*,*P* = 0.48	*8(43)*,*P* = 0.42
*Q*_B_	**15.5**	**23.4**	**8.8**	7.5	5.2	0.7
*P*	**0.017**	**<0.001**	**0.031**	0.11	0.63	0.95

The weighted mean sensitivities (means [–95%CI, + 95%CI]) under eCO_2_ (% + 100 ppm^–1^), eT (% + 1 °C^–1^), iP (% + 10%^–1^), dP (% – 10%^–1^), eN (% + 1 g^–1^ m^–2^ yr^–1^), and eO_3_ (% + 10 ppb^–1^) are reported. Sensitivities significantly different from zero at *P* < 0.05 are shown in bold, with the sample size of species (observation) shown in italic. *Q*_B_: between-group heterogeneity, significant *Q*_B_ at *P* < 0.05 which indicates that the sensitivities differ among biomes are shown in bold. See Fig. 1 for variable abbreviations.

### Interactions between global change factors

**Fig. 5:**
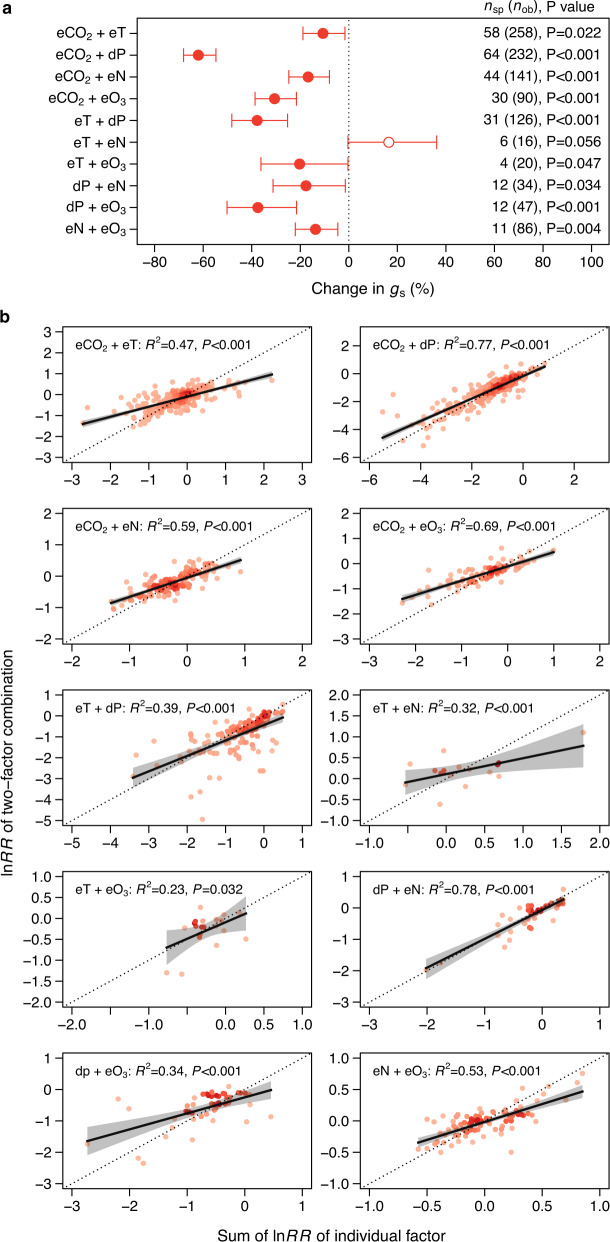
Interactive effects between global change factors on *g*_s_. **a** The overall changes of *g*_s_ in response to two-factor combinations. The error bars represent ±95% of the confidence interval for the weighted means with filled and open points indicating significant (*P* < 0.05) and insignificant (*P* > 0.05) differences from zero, respectively. The numbers outside and inside parentheses represent the number of species (*n*_sp_) and observations (*n*_ob_), respectively. **b** Relations between ln*RR* (natural log-transformed response ratio) of two-factor combination and sum of ln*RR* of the corresponding individual factor. The error bands surrounding the regression lines represent the 95% confidence interval. See Fig. 1 for variable abbreviations.

The GCFs’ effects can be additive, antagonistic, or synergistic according to whether the combined effect size is equal, smaller, or larger than the sum of the individual effect size (Fig. S7). All two-factor combinations of GCFs significantly reduced *g*_s_ except eT+eN, which did not significantly change *g*_s_ (Fig. 5a). The interactions between iP and other GCFs were unable to test due to data availability. The individual effects were commonly additive as most points fell around the 1:1 line, but tended to be antagonistic with increasing effect sizes, where the combined effect sizes were smaller than the sum of the individual effect sizes (Fig. 5b).

### Future changes in stomatal conductance

**Fig. 6:**
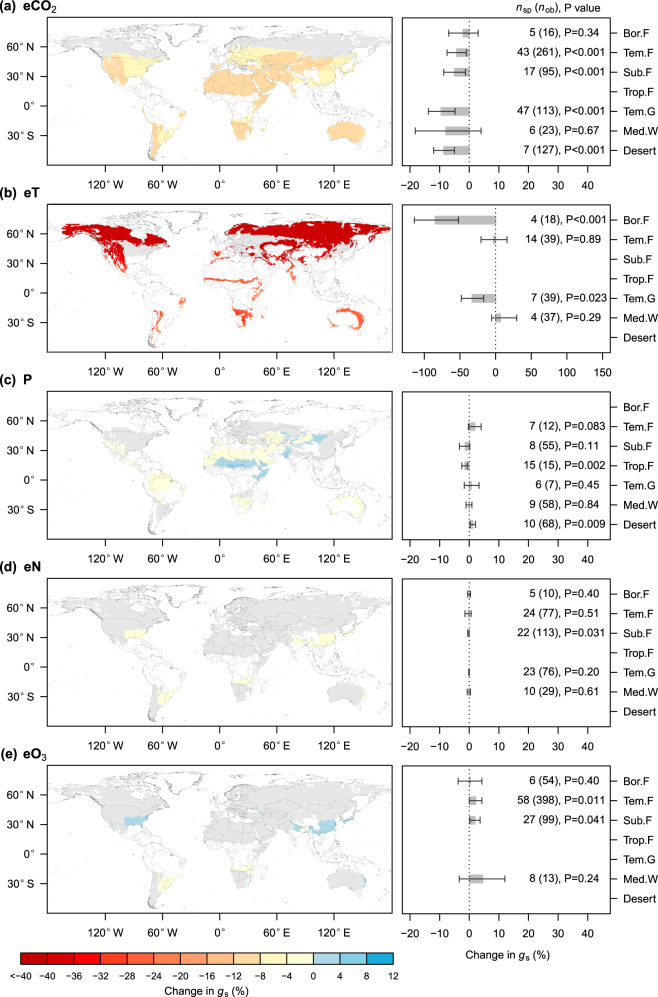
Predicted changes in *g*_s_ across biomes by the end of the twenty-first century under the sustainable emission scenario (SSP1-2.6/RCP2.6). **a** Changes induced by elevated CO_2_. **b** Changes induced by warming. **c** Changes induced by changed precipitation. **d** Changes induced by elevated N deposition. **e** Changes induced by elevated O_3_. The left panels display global maps depicting changes in *g*_s_, with grey and white land colors indicating areas where changes are statistically insignificant and where is a lack of data, respectively. The right panels depict biome-level predictions, with error bars representing the 95% confidence interval. The numbers outside and inside parentheses represent the number of species (*n*_sp_) and observations (*n*_ob_), respectively. Bor.F: boreal forest, Tem.F: temperate forest, Sub.F: subtropical forest, Trop.F: tropical forest, Tem.G: temperate grassland, Med.W: Mediterranean woodland.

We examined how individual GCFs alone might change the *g*_s_ of terrestrial plants by the end of this century based on the predicted magnitudes of changes in different GCFs and the stomatal sensitivities revealed in this study. Under the sustainable emission scenario (SSP1-2.6/RCP2.6), eCO_2_ would significantly reduce *g*_s_ for temperate forests, subtropical forests, temperate grassland, and desert plants, with reduction magnitudes ranging from 4.3% to 9.5% (Fig. 6a). eT would significantly reduce *g*_s_ for boreal forest and temperate grassland by 84.8% and 33.1%, respectively (Fig. 6b). Compared with eCO_2_ and eT, the effects of changed precipitation, eN, and eO_3_ on *g*_s_ would be rather small (Fig. 6c–e). The relative impacts of GCFs were consistent across scenarios (Figs. S8 and 9), i.e., rising CO_2_ and warming would dominate future changes in *g*_s_ across biomes.

## Discussion

The land surface component of fully coupled climate-carbon cycle models is highly sensitive to the stomatal formulation^27,28^. Hence understanding the stomatal response of terrestrial plants to global change is key to improve future coupled water and carbon cycle predictions. Recent meta-analyzes have provided important perspectives on the stomatal response to several GCFs, including eCO_2_^7,19–22,24^, eN^23^, and eO_3_^25,26^. Our global data synthesis quantified the stomatal responses to five major GCFs across vegetation biomes and revealed two main findings to improve our understanding of stomatal response to global change. First, we showed that the effects between GCFs were commonly additive but tended to be antagonistic as effect sizes increased. Second, we demonstrated that rising CO_2_ and warming would dominate future changes in *g*_s_ across biomes.

The overall response patterns of *g*_s_ to various GCFs are generally consistent with theory predictions shown in Fig. 1. A recent study showed that *g*_s_ responses to eCO_2_ can be predicted by the optimal stomatal theory, which holds that plants regulate stomata to maximize photosynthesis and minimize water transpiration to achieve optimal water use efficiency^29^. In the past decades, the decline of *g*_s_ is one of the most consistent responses of plants to eCO_2_ that has been documented^7,8,20–22^. The eCO_2_ can reduce *g*_s_ by decreasing stomatal aperture in the short term and/or stomatal density and size in the long term^5,7^, but long-term eCO_2_ does not necessarily reduce stomatal density^30^. It has been illustrated that, even though the relative contributions of changes in stomatal aperture vs changes in stomatal density and size differed, short-term and long-term eCO_2_ resulted in similar *g*_s_ responses^6^, in line with our result that *g*_s_ sensitivity did not vary with experimental duration (Fig. S3g).

We found that conifer trees exhibited the lowest sensitivity to eCO_2_ compared to other plant functional types, in line with previous findings that the stomata of gymnosperm trees were less sensitive to eCO_2_^31^. This pattern was associated with their overall lower ambient *g*_s_, because stomatal sensitivity to eCO_2_ declined significantly with decreasing ambient *g*_s_ (Fig. 4g). As stomata respond to CO_2_ concentration at the intercellular space rather than the leaf surface^32^, it is reasonable that plants with higher ambient *g*_s_ responded more strongly to eCO_2_ because it allows more gases to enter and lead to greater increases of CO_2_ in the intercellular space. The lower stomatal sensitivity of conifers to eCO_2_ and the pattern that stomatal sensitivity reduced with decreasing mean annual temperature (MAT, Fig. S4g) together explained why *g*_s_ of boreal forests were not sensitive to eCO_2_ (Table 1).

Warming is considered to affect plant *g*_s_ indirectly via Rubisco activity (*V*_cmax_, and consequently photosynthesis and its linkage to *g*_s_), vapor pressure deficit (VPD), and plant water status^10^. Both consistent^33^ and inconsistent^34^ changes between *g*_s_ and *V*_cmax_ have been reported, and warming could change *g*_s_ independently with VPD in some species^35^, suggesting complex interactions among these mechanisms. It has been proposed that with increasing temperature, *g*_s_ first increases and peaks at an optimal temperature and then decreases at high temperature^10^. Since the differences between optimal temperatures and growth temperatures might vary with biomes^36^, the response of plant *g*_s_ to warming is difficult to predict from theory. Here our meta-analysis revealed that warming overall significantly reduced *g*_s_ (Fig. 2), and boreal forests showed the highest sensitivity to eT (Table 1). As mentioned above, it should be noted that lowered *g*_s_ under eT were linked to lowered photosynthetic rates in some but not all cases^37,38^.

Stomatal responses to changed precipitation are mainly regulated by abscisic acid^11^. In line with the theory prediction, iP (increased precipitation) significantly enhanced *g*_s_ and dP (decreased precipitation) decreased it. However, there were no significant differences in the stomatal sensitivity to dP among biomes, while the stomatal sensitivity to iP varied significantly across biomes (Table 1). Plants grown in drier habitats with lower ambient *g*_s_ were more sensitive to iP (Figs. 4i and S5i) but showed no differences in the sensitivity to dP (Figs. 4j and S5j). The results suggest that stomatal sensitivities across biomes are convergent in the responses to drought while divergent in the responses to increased precipitation.

The effect of eN on plant *g*_s_ is most likely indirect, and recent studies suggest two potential mechanisms. First, higher N availability stimulates plant *g*_s_ as a byproduct of enhanced photosynthetic rate, as evidenced by a positive correlation between the responses of these two traits under eN^23^. Second, N enrichment might deplete soil cations including calcium, which is important for the control of the stomatal aperture^39^. Our results showed that eN significantly increased plant *g*_s_, with a threefold greater enhancement for the indoor than the outdoor experiments (Fig. S2a). A previous meta-analysis showed that compared with field experiments, indoor N addition induced a twofold greater enhancement of plant biomass^40^, suggesting that indoor N addition stimulated photosynthetic rate and, as a byproduct, plant *g*_s_ to a greater magnitude.

Elevated O_3_ may reduce *g*_s_ directly via producing reactive oxygen species that actively participate in the regulation of the stomatal aperture^13,25^ or indirectly as a symptom of a decline in photosynthetic rate^14^. Our findings that eO_3_ significantly reduced *g*_s_ confirm previous results of meta-analyzes^25,26^. Besides, the result that plants with greater ambient *g*_s_ were more sensitive to eO_3_ (Fig. 4l) can be explained by a unifying theory, which proposes that cumulative uptake of O_3_ into the leaf universally controls the responses of plants (including photosynthesis and growth) across plant functional types^41^. Accordingly, greater treatment magnitude and ambient *g*_s_, both facilitating the cumulative uptake of O_3_, are more likely to result in larger stomatal responses.

In the real world, different GCF combinations might occur in a given terrestrial ecosystem, but any GCFs will co-occur with eCO_2_^42^. For combined eCO_2_ and other GCFs, their effects on *g*_s_ were commonly additive but tended to be antagonistic when the effect sizes became larger (Fig. 5b). The result indicates that eCO_2_ might partly diminish the effect of other GCFs on stomata. For instance, it is possible that the lowered *g*_s_ caused by eCO_2_ reduced the uptake and thus the negative impact of O_3_^41^. Similar to our results, prior studies also showed that the GCFs’ effects tended to be antagonistic as the effect sizes became larger for plant biomass^43^, plant N:P ratio^44^, and plant diversity^45^. Interestingly, all these studies used the same method (i.e., paired meta-analyzes), and different methods might lead to contrasting conclusions. For example, studies using Hedges’ *d* method reported that GCFs’ effects on plant biomass and carbon storage were overall additive^15,46^. The Hedges’ *d* method did not reveal how the interactions vary with effect sizes^44^. The results highlight the importance of the analytical method and imply that models might overestimate the global change effects if additive effects among GCFs are assumed when the effect sizes become larger.

Among the GCF combinations we evaluated, how eCO_2_ interacts with eN and dP is of particular interest. First, the progressive nitrogen limitation hypothesis proposes that available nitrogen becomes increasingly limiting under eCO_2_ as it is sequestered in long-lived plant biomass and soil organic matter^47^. Our dataset cannot directly test this hypothesis, but we found interesting interactions between eCO_2_ and eN. Although eN alone significantly increased *g*_s_, combined eN and eCO_2_ significantly decreased it (Figs. 2a and 5a), suggesting that eCO_2_ overrode the effect of eN on *g*_s_. The results highlight the importance of expanding global change experiments from single to multiple factors in interaction, especially when the effects conflict between GCFs as they did between eCO_2_ and eN. Second, there is a long-standing hypothesis that rising CO_2_ will ameliorate the impact of drought on plants^48^. Water saved by having lower *g*_s_ under eCO_2_ in the initial period of drought can be used to keep stomata open longer, for photosynthesis in the later phase of drought^49^. Therefore, *g*_s_ under eCO2+dP may decline less rapidly and transform from being lower to being higher than under dP alone with time. Some studies did show greater *g*_s_ under eCO_2_ + dP than under dP alone^50,51^. However, the water-saving effect of eCO_2_ at the ecosystem scale, if any, would be smaller than the *g*_s_ response at the leaf scale, which is often related to the stimulation of the leaf area index (LAI) of the canopy^17^. Besides, we argue that although eCO_2_ may save water during the initial phase of drought^52^, it might inversely increase water loss as drought persists. One reason for this is that under severe droughts, plants continue to lose water via the leaf cuticle and incompletely closed stomata in spite of predominately closed stomata. Hence, water loss from leaves continues through minimum leaf conductance (*g*_min_) rather than mean *g*_s_^53^. As LAI would increase under eCO_2_^54^, canopy water loss might also increase if *g*_min_ is not affected. Secondly, though not directly addressed in most experiments, species competition for survival during water-limited periods might involve water use rather than water savings^55,56^. Together, the findings involving eCO_2_ interactions with eN and dP support an imperative for future experiments to further address interactions with eCO_2_ in large-scale manipulations.

Compared with other GCFs, rising CO_2_ and warming would exert a greater effect on *g*_s_ across biomes in the future (Fig. 6). This is due to the greater *g*_s_ sensitivity to eCO_2_ and eT (Table 1), as well as greater predicted change magnitude in CO_2_ concentration by the end of this century^42,57^. Under SSP2-4.5 and SSP5-8.5, a few values of predicted *g*_s_ change magnitude in Figs. S8 and 9 are unreliable for several biomes, including boreal forests, which may be related to the nonlinear response patterns to eCO_2_^6,19,58^ and eT^10^. Overall, we found that *g*_s_ was reduced nonlinearly with increasing CO_2_ (Fig. 3a) and the *g*_s_ sensitivity decreased gradually with rising CO_2_ (Fig. 3g), suggesting a decreasing marginal effect of eCO_2_. The decreasing marginal effect is even more evident when comparing our results from experiments with those from historical records. For example, over the past 150 years, when CO_2_ was increased from 290 ppm to 390 ppm, *g*_s_ was reduced by 34% per 100 ppm CO_2_ increase for plants grown in Florida^59^, while in the eCO_2_ experiments where CO_2_ was on average increased from 370 ppm to 700 ppm, *g*_s_ was reduced by only 8.2% per 100 ppm CO_2_ increase. However, for the warming experiments, we could not derive a nonlinear relationship between *g*_s_ and temperature^60^. For the boreal forest biome, the *g*_s_ sensitivity to eT was derived from relatively low warming magnitudes (0.7–1.3 °C); such sensitivity may not be applied to future warming magnitudes under SSP2-4.5 (2.0–7.3 °C) and SSP5-8.5 (3.7–12.5 °C). Besides, due to the same reason, the prediction that eT would reduce the *g*_s_ of boreal forests by 84.8% under SSP1-2.6 with warming magnitudes of 1.1–5.3 °C (Fig. 6b) should be employed with caution. The results highlight the importance of dose-response curves between *g*_s_ and GCFs for predictions, which represent significant challenges for future global change manipulations since the cost of covering a wider treatment range of GCFs will be much higher.

Stomatal conductance plays a critical role in plant carbon and water flux. Earth system models integrating the stomatal responses to CO_2_ yielded very different projections of future global patterns of precipitation^61^ and river runoff^62^. Our findings can inform models by providing stomatal sensitivities of different biomes and plant functional types to major GCFs. Another implication of our results for models is that co-occurring eCO_2_ and other GCFs would exert antagonistic effects on *g*_s_ when the effect sizes become larger, and the combined effects would be overestimated if additive effects are assumed.

There are mainly three limitations to the current data synthesis. First, global change experiments are rare in tropical forests, although they represent the most important biomes impacting global carbon and water cycles and have been shown to be sensitive to global changes^15^. While the Coupled Model Intercomparison Project Phase 5 (CMIP5) models assume a great stomatal sensitivity to eCO_2_ in tropical regions^63^, empirical data remain lacking. Second, experiments with three or more GCFs are needed to confirm and expand the generality of the *g*_s_ response patterns observed here. But our results also highlight the influences of rising CO_2_ and temperature, suggesting that future studies would need to focus on the overall or net effects of climate change drivers on *g*_s_. Third, dose-response curves are critical for accurately predicting future *g*_s_ under different emission scenarios, but we could only derive an average dose-response curve between *g*_s_ and eCO_2_ based on current data. Thus a great challenge for future global change manipulations would be investigating the dose-response curves for different GCFs across species and biomes. Resolving these limitations in future experiments will significantly improve our understanding and prediction of *g*_s_ and terrestrial carbon and water cycling in a changing climate.

## Methods

### Data sources and compilation

Published papers that reported *g*_s_ responses to GCFs were searched in Web of Science, using the following keywords: TS = (stomatal conductance OR gs) AND TS = (carbon dioxide OR CO2 OR warming OR increasing temperature OR elevated temperature OR precipitation OR rainfall OR drought OR water stress OR nitrogen deposition OR N deposition OR nitrogen addition OR N addition OR ozone OR O3). In addition, papers cited in previous reviews and meta-analysis articles that studied plant responses to global changes were also surveyed. All the papers were further selected by two steps (Fig. S10), and finally, 616 articles (Supplementary References) that met the following criteria were included in the data synthesis:

- Studies were conducted in terrestrial ecosystems, including forests, deserts, grasslands, and croplands. Both outdoor and indoor studies were included as the *g*_s_ sensitivities were not significantly different between the two experimental approaches (Fig. S2). However, only the outdoor studies were included when analyzing the *g*_s_ responses across biomes and the relationships with climates because, for the indoor studies, plants were grown in a controlled environment and thus did not reflect the influence of the local climates.
- Studies examined the effects of simulated GCFs (i.e., eCO_2_, eT, iP/dP, eN, eO_3_), individually or in combination. Each study must include control and treatment groups representing the current and future environment, respectively. For instance, experiments had different N input levels but using artificially potting soil (i.e., the mixture of sand, clay, and vermiculite, etc.) or adding P/K (or Hoagland’s solution) were excluded because they could not represent the effects of N deposition on natural ecosystems.
- *g*_s_ was measured as mole H_2_O of flux per unit of leaf area (mol m^−2^ s^−1^) under certain reference conditions (i.e., near-saturation light intensity, treatment-depended CO_2_ concentration and temperature, and prevailing humidity) using infrared gas analyzers such as LI-6400/6800 (LI-COR, Lincoln, USA), CIRAS-2/3 (PP Systems, Hitchin, UK), and LCA-2/3/4 (ADC-Biosciences, Hoddesdon, UK).
- Studies reported the mean values and standard errors of *g*_s_ of both control and treatment groups with at least three replicates (*n* ≥ 3).

Data were extracted from the tables, figures, text, and supporting information of the source papers. The Web Plot Digitizer (https://apps.automeris.io/wpd) was used to extract data from figures. All relevant observations provided by the source papers were extracted. Observations from different sites or species were considered independent, but multiple observations of the same species at the same site were considered non-independent and treated using the “shifting the unit of analysis” approach (see Text below). Experimental factors which might affect the *g*_s_ responses including treatment magnitude and experimental duration were also extracted. Basic climatic parameter values (MAT, AI, and water vapor pressure (VP)) were obtained from Worldclim (http://www.worldclim.org) based on the site locations if site climate information was not provided in the source papers. VP at saturation (VPsat) was calculated as $a\times {{{{{\rm{exp }}}}}}\left[b\times {{{{{\rm{T}}}}}}/\left(c+{{{{{\rm{T}}}}}}\right)\right]$, where *a*, *b*, *c*, and T are constants of 0.611 kPa, 17.502 (unitless), 240.97 °C, and monthly air temperature, respectively, and${{{{{\rm{VPD}}}}}}={{{{{\rm{VPsat}}}}}}-{{{{{\rm{VP}}}}}}$. According to the classification of the Koppen-Geiger climatic zones, the biomes of outdoor experiments were categorized into boreal forest, temperate forest, subtropical forest, tropical forest, temperate grassland, Mediterranean woodland, desert, or cropland based on the site locations using the R package ‘kgc’ (https://cran.r-project.org/web/packages/kgc/). Plant species were classified into eight plant functional types: conifer, deciduous broad-leaved tree, evergreen broad-leaved tree, shrub, C3 grass, C4 grass, legume forb, or nonlegume forb. All data used in this study can be accessed in the Supplementary Data file.

### Meta-analysis

The effect size was estimated using the natural log-transformed response ratio (${{{{\mathrm{ln}}}}}{RR}$) ^64^:

1 $${{{{{\mathrm{ln}}}}}}{RR}={{{{{\mathrm{ln}}}}}}\left({\overline{{X}}}_{{{{{\rm{T}}}}}}/{\overline{{X}}}_{{{{{\rm{C}}}}}}\right)$$

with a variance of:

2 $${v}_{{{{{{\rm{lnRR}}}}}}}=\frac{{{{{{{\rm{SE}}}}}}}_{T}^{2}}{{\bar{X}}_{T}^{2}}+\frac{{{{{{{\rm{SE}}}}}}}_{C}^{2}}{{\bar{X}}_{C}^{2}}+{\tau }^{2}$$

where ${\bar{X}}_{{{\mbox{T}}}}$ (and ${{SE}}_{{{\mbox{T}}}}$) and ${\bar{X}}_{{{\mbox{C}}}}$ (and ${{SE}}_{{{\mbox{C}}}}$) represent the mean (and standard error) of the treatment and control group, respectively; ${\tau }^{2}$ was the between-studies variance and estimated using the R package ‘metafor’ 3.0-2^65^.

The original weighting factor (*w*) of each effect size was given as^64,65^:

3 $${{\mbox{w}}}=1/{{{\mbox{v}}}}_{{{{{\mathrm{ln}}}}}{{\mbox{RR}}}}$$

When calculating the overall response ratio, the unit of analysis was a species in a site, that is, multiple observations for the same species in the same site were considered non-independent. A “shifting the unit of analysis” method^66^ was used to take this non-independence into account. Specifically, the non-independent response ratios were averaged within species within sites, by adjusting the weight of each effect size as^23,67^:

4 $${w}^{\ast }=w/{n}_{{{{{{\rm{ob}}}}}}}$$

where ${w}^{*}$ was the adjusted weighting factor of each effect size, *n*_ob_ was the total number of observations for the same species at the same site.

The overall *g*_s_ response was quantified using the weighted mean response ratio ($\overline{{{{{{\rm{ln}}}}}}RR}$):

5 $$\overline{{{{{{\rm{ln}}}}}}RR}=\frac{{\sum }_{{{{{{\rm{i}}}}}}=1}^{m}{w}_{i\,}^{\ast }\times {{{{{{\rm{lnRR}}}}}}}_{i}}{{\sum }_{{{{{{\rm{i}}}}}}=1}^{m}{w}_{i}^{\ast }}$$

where *m* was the total number of observations for a given GCF or GCFs combination, ${{{{{{\rm{ln}}}}}}{{{{{\rm{RR}}}}}}}_{i}$ and ${w}_{i}^{*}$ were the natural log-transformed response ratio and the adjusted weighting factor of the *i*th observation, respectively.

The standard error of $\overline{{{{{{\rm{ln}}}}}}RR}$ was calculated as:

6 $${{{{{{\rm{SE}}}}}}}_{\overline{{{{{{\rm{ln}}}}}}RR}}=\sqrt{\frac{1}{{\sum }_{{{{{{\rm{i}}}}}}=1}^{m}{w}_{i}^{\ast }}}$$

And the 95% confidence interval (CI) for $\overline{{{{{{\rm{ln}}}}}}RR}$ was calculated as:

7 $$95\%\,{{{{{\rm{CI}}}}}}=\overline{{{{{{\rm{ln}}}}}}RR}\pm 1{.96\times {{{{{\rm{SE}}}}}}}_{\overline{{{{{{\rm{ln}}}}}}RR}}$$

The $\overline{{{{{{\rm{ln}}}}}}RR}$ with 95% CI was estimated with the random-effects model in R using the package ‘metafor’ 3.0–2^65^. Finally, $\overline{{{{{{\rm{ln}}}}}}RR}$ was transformed to percentage change (%) as:

8 $${Percentage\; change}=({{{{{\rm{exp}}}}}} ({\overline{{{{{{\rm{ln}}}}}}RR}})-1)\times 100$$

The impacts of treatment magnitude, plant attributes (ambient *g*_s_, vegetation biomes, and plant functional types), and climate on the *g*_s_ responses were assessed via the meta-regression function of the package ‘metafor’. Both linear and nonlinear regressions were performed, and the nonlinear meta-regressions were conducted by fitting the “restricted cubic splines” model with the additional help of the ‘rms’ package. Results of the better-performed linear or nonlinear regressions with higher R^2^ values were reported. The publication bias was evaluated by funnel plots. Funnel plot asymmetry was further tested with Egger’s regression in R using the ‘metafor’ package. Results showed all the studies except eT+dP were distributed symmetrically around the mean effect size in the funnel plots (Fig. S11), suggesting publication bias only might exist in the warming and drought combined experimental data.

### Stomatal sensitivity to global change factors

The natural log-transformed *g*_s_ sensitivity (${{{{\mathrm{ln}}}}}{Sens}$) was calculated as ^68^:

9 $${{{{\mathrm{ln}}}}}\,Sens=\frac{{{{{\mathrm{ln}}}}}\,RR}{\varDelta }$$

with a variance of:

10 $${v}_{{{{{{\rm{sens}}}}}}}=\frac{{v}_{{{{{{\rm{RR}}}}}}}}{{\varDelta }^{2}}$$

where ${{{{\mathrm{ln}}}}}{RR}$ was the natural log-transformed response ratio, ${v}_{{{\mbox{RR}}}}$ was the variance of ${{{{\mathrm{ln}}}}}{RR}$, and $\triangle$ was the treatment magnitude in standardized units^69^. Here, the absolute magnitudes were used for eCO_2_ (per 100 ppm increase), eT (per 1 °C increase), eN (per 1 g m^–2^ yr^–1^ increase), and eO_3_ (per 10 ppb increase), and the relative magnitude was used for iP/dP (per 10% change), according to previous studies^15,68^. The weighted means of ${{{{\mathrm{ln}}}}}{Sens}$ and percentage sensitivity were calculated similarly to the $\overline{{{{{{\rm{ln}}}}}}RR}$ (Eq. 5) and ${{{{{\rm{Percentage}}}}}}\;{{{{{\rm{change}}}}}}$ (Eq. 8) above.

### Interactions between global change factors

The interactions were evaluated via paired meta-analyzes, a conservative approach by comparing the effect size of combined factors with the sum of effect sizes of the corresponding individual factors^43,44^. For positive summed effect sizes, the synergistic, antagonistic, and additive interactions should fall above, below and on the 1:1 line, corresponding to larger (more positive), smaller (less positive), and equal combined effect sizes. For negative summed effect sizes, the synergistic, antagonistic, and additive interaction should fall below, above, and on the 1:1 line, corresponding to larger (more negative), smaller (less negative), and equal combined effect sizes, respectively (Fig. S7).

### Impacts of future global change

The global change impacts on *g*_s_ by the end of the twenty-first century were evaluated by multiplying the *g*_s_ sensitivities with the projected change magnitudes of GCFs under different emission scenarios. To make the predictions spatially explicit, we used the biome-specific *g*_s_ sensitivities (Table 1) combined with maps of global changes in different GCFs. For atmospheric CO_2_ concentrations, present and future conditions were produced by averaging data from the Mauna Loa Observatory, Hawaii (http://CO2now.org) for the period 1981–2000 and the SSP Database (https://tntcat.iiasa.ac.at/SspDb/) for the period 2081–2100, respectively (Table S2). For temperatures and precipitations, the bioclimatic variables ‘mean annual temperature’ (BIO1) and ‘annual precipitation’ (BIO12) in the WorldClim v2.1 climate database (https://worldclim.org) were used. In WorldClim, present and future conditions were produced by averaging historical data for the period 1970–2000 and averaging data from eight general circulation models (GCMs) (Table S2) for the period 2081–2100, respectively. For N depositions, average values from seven models (Table S2) of the Coupled Model Intercomparison Project Phase 6 (CMIP6) datasets for the period 1981–2000 and 2081–2100 were used. For ground-level O_3_ concentrations, current and future conditions were produced via averaging data from six global atmospheric chemistry transport models (Table S2) during a period centered around 2000 and 2100, respectively^70^. Three future Shared Socioeconomic Pathways (SSP) scenarios were used for the predicted future changes in CO_2_ concentration, temperature, precipitation, and N deposition: (1) the SSP1-2.6, a sustainable scenario that respects perceived environmental boundaries; (2) the SSP2-4.5, ‘middle of the road’ scenario which emission trends do not shift markedly from historical patterns; and (3) the SSP5-8.5, rapid economic and social development coupled with the exploitation of abundant fossil fuel resources. The SSPs are not yet available for ground-level O_3_, thus the corresponding Representative Concentration Pathway (RCP) scenarios (RCP2.6, RCP4.5, and RCP8.5, respectively) were used. Only the predictions of the direct effects of each GCF on *g*_s_ were made because there were complex combinations of multiple GCFs in a specific location that were unable to be taken into account using currently available data. The average changes of *g*_*s*_ with 95% CI at the biome level were estimated by calculating the average change magnitude of each GCF, and multiplying it by the weighted mean sensitivity with 95% CI of each GCF in Table 1.

### Reporting summary

Further information on research design is available in the Nature Portfolio Reporting Summary linked to this article.

## Supplementary information

Supplementary Information

Supplementary Data 1

Reporting Summary

## Data Availability

The data generated in this study are provided in the Supplementary Data file.

## Code availability

The code supporting the findings of meta-analysis presented here is available at Figshare (10.6084/m9.figshare.21800822.v4).
