# Supplementary material for: Stomatal responses of terrestrial plants to global change
Source: Nat Commun. 2023 Apr 17;14:2188. doi: 10.1038/s41467-023-37934-7 (PMC10110556; doi:10.1038/s41467-023-37934-7)
Supplement: Supplementary file 1 — Supplementary Information [file 41467_2023_37934_MOESM1_ESM.pdf]

Supplementary Information of

Stomatal responses of terrestrial plants to global change

Xingyun Liang[1]; Defu Wang[1]; Qing Ye[1,2*]; Jinmeng Zhang[3]; Mengyun Liu[4]; Hui Liu[1,5]; Kailiang Yu[6,7]; Yujie Wang[8]; Enqing Hou[1,5]; Buqing Zhong[1,5]; Long Xu[1]; Tong Lv[9]; Shouzhang Peng[9]; Haibo Lu[10]; Pierre Sicard[11]; Alessandro Anav[12]; David S. Ellsworth[13]

[1] *Guangdong Provincial Key Laboratory of Applied Botany, South China Botanical Garden, Chinese Academy of Sciences, Xingke Road 723, Guangzhou 510650, China;*

[2] *College of Life Sciences, Gannan Normal University, Ganzhou 341000, China;*

[3] *School of Geographical Sciences, Jiangsu Second Normal University, Nanjing 211200, China;*

[4] *Guangdong Provincial Key Laboratory of Silviculture, Protection and Utilization, Guangdong Academy of Forestry, Guangzhou, Guangdong 510520, China;*

[5] *Key Laboratory of Vegetation Restoration and Management of Degraded Ecosystems, South China Botanical Garden, Chinese Academy of Sciences, Guangzhou 510650, China;*

[6] *Department of Ecology & Evolutionary Biology, Princeton University, NJ, USA;*

[7] *High Meadows Environmental Institute, Princeton University, NJ, USA;*

[8] *Division of Geological and Planetary Sciences, California Institute of Technology, Pasadena, California 91125, USA;*

[9] *State Key Laboratory of Soil Erosion and Dryland Farming on the Loess Plateau, Northwest A&F University, Yangling 712100, China;*

[10] *Department of Geography, Faculty of Arts and Sciences, Beijing Normal University, Zhuhai 519087, China;*

[11] *ARGANS Ltd, 260 route du Pin Montard, 06410 Biot, France;*

[12] *ENEA, Climate Modeling Laboratory, CR Casaccia, 301 Via Anguillarese, 00123 Rome, Italy;*

[13] *Hawkesbury Institute for the Environment, Western Sydney University, Penrith, NSW, Australia.*

[*] **Corresponding author:** Qing Ye (qye@scbg.ac.cn)

Table S1 | Global environmental change sensitivity of stomatal conductance across plant functional types. The weighted mean sensitivities (means [–95%CI, +95%CI]) under eCO_2 (% + 100 ppm^{-1}), eT (% + 1 °C^{-1}) , iP (% + 10%$^{-1}$) , dP (% – 10%$^{-1}$), eN (% + 1 g^{-1} m^{-2} yr^{-1}), and eO_3 (% + 10 ppb^{-1}) are reported. Sensitivities significantly different from zero at $P < 0.05$ are shown in bold, and the numbers of species (observation) are shown in italic. Q_B: between-group heterogeneity, significant Q_B at $P < 0.05$ are shown in bold and indicate that the sensitivities differ among plant functional types. eCO_2: elevated CO_2 concentration, eT: elevated temperature, iP/dP: increased/decreased precipitation, eN: elevated nitrogen deposition, and eO_3: elevated O_3 concentration.

Plant functional type	eCO2	eT	iP	dP	eN	eO3
Conifer	**–3.6 [–6.4, –0.7]**	–2.6 [–5.3, 0.3]	5.5 [–0.1, 11.3]	**–3.7 [–5.7, –1.6]**	**1.2 [0.1, 2.3]**	**–2.4 [–4.3, –0.6]**
	26(141),P=0.015	*24(81),P=0.078*	*5(10),P=0.052*	*10(86),P<0.001*	*16(63),P=0.027*	*15(126),P=0.010*
Deciduous broadleaf	**–7.1 [–9.2, –5.0]**	–2.7 [–5.5, 0.2]	2.0 [–2.8, 7.0]	**–3.4 [–6.6, -0.0]**	–0.1 [–0.8, 0.5]	–1.3 [–2.7, 0.2]
	66(406) ,P<0.001	*22(91),P=0.069*	*4(20),P=0.43*	*15(75),P=0.047*	*26(96),P=0.70*	*52(461),P=0.089*
Evergreen broadleaf	**–10.3 [–12.8, –7.7]**	–0.4 [–2.5, 1.6]	–3.6 [–7.7, 0.7]	**–4.8 [–7.6, –1.8]**	**1.7 [1.0, 2.3]**	**–2.1 [–3.9, –0.2]**
	47(114),P<0.001	*20(64),P=0.69*	*3(9),P=0.10*	*19(40),P=0.002*	*21(66),P<0.001*	*26(75),P=0.030*
Shrub	**–7.2 [–12.5, –1.6]**	1.3 [–0.8, 3.5]	**3.4 [0.8, 6.1]**	–1.6 [–3.7, 0.6]	0.9 [–0.4, 2.3]	–2.9 [–6.8, 1.2]
	17(161),P=0.013	*10(70),P=0.21*	*6(53),P=0.011*	*7(30),P=0.14*	*12(39),P=0.17*	*5(6),P=0.17*
C3 grass	**–10.4 [–13.0, –7.8]**	–0.1 [–1.7, 1.5]	3.1 [–5.8, 12.9]	**–3.8 [–6.0, –1.5]**	**1.4 [0.7, 2.2]**	**–3.1 [–5.8, –0.3]**
	35(267),P<0.001	*13(183),P=0.87*	*2(2),P=0.51*	*6(21),P=0.001*	*9(54),P<0.001*	*10(49),P=0.029*
C4 grass	**–8.8 [–13.8, –3.5]**	–1.3 [–4.0, 1.5]	0.9 [–6.6, 8.9]		**1.5 [0.3, 2.2]**	0.7 [–4.1, 5.7]
	17(56),P=0.001	*4(22),P=0.36*	*2(5),P=0.82*		*8(23),P=0.012*	*3(6),P=0.77*
Legume forb	**–11.3 [–14.1, –8.4]**	0.4 [–4.7, 5.8]	/	**–4.7 [–8.0, –1.2]**	0.4 [–1.9, 2.9]	–3.2 [–7.0, 0.8]
	18(109),P<0.001	*4(15),P=0.88*		*4(4),P=0.009*	*7(7),P=0.72*	*4(4),P=0.12*
Nonlegume forb	**–8.7 [–12.8, –4.5]**	**–1.9 [–3.4, –0.4]**	2.1 [–7.2, 12.4]	–2.6 [–5.4, 0.2]	0.3 [–1.8, 2.4]	–1.7 [–5.1, 1.8]
	36(97),P<0.001	*22(78),P=0.015*	*2(2),P=0.67*	*5(16),P=0.073*	*14(53),P=0.80*	*6(16),P=0.34*
Q_B	**21.8**	10.2	9.1	5.4	**18.9**	3.6
P	**0.003**	0.18	0.17	0.61	**0.008**	0.82

Table S2 | Source of spatial data of future changes in environmental factors

Factors	Sources	Models	Future scenarios
Atmospheric CO_2 concentration	Mauna Loa Observatory<br>SSP Database		SSP1-2.6<br>SSP2-4.5<br>SSP5-8.5
Atmospheric temperature<br>Precipitation	Worldclim v2.1	BCC-CSM2-MR<br>CNRM-CM6-1<br>CNRM-ESM2-1<br>CanESM5<br>IPSL-CM6A-LR<br>MIROC-ES2L<br>MIROC6<br>MRI-ESM2-0	SSP1-2.6<br>SSP2-4.5<br>SSP5-8.5
Atmospheric N deposition	CMIP6 datasets	CESM2-WACCM<br>MIROC-ES2L<br>CMCC-ESM2<br>CMCC-CM2-SR5<br>MPI-ESM1-2-LR<br>EC-Earth3-Veg<br>ACCESS-ESM1-5	SSP1-2.6<br>SSP2-4.5<br>SSP5-8.5
Ground-level O_3 concentration	Sicard *et al.*, 2017	CESM-CAM<br>GFDL-AM3<br>GISS-ES2-R<br>MIROC-CHEM<br>MOCAGE<br>UM-CAM	RCP2.6<br>RCP4.5<br>RCP8.5

References

Sicard P, Anav A, De Marco A, Paoletti E. Projected global ground-level ozone impacts on vegetation under different emission and climate scenarios. *Atmos. Chem. Phys.* **17**, 12177-12196 (2017).

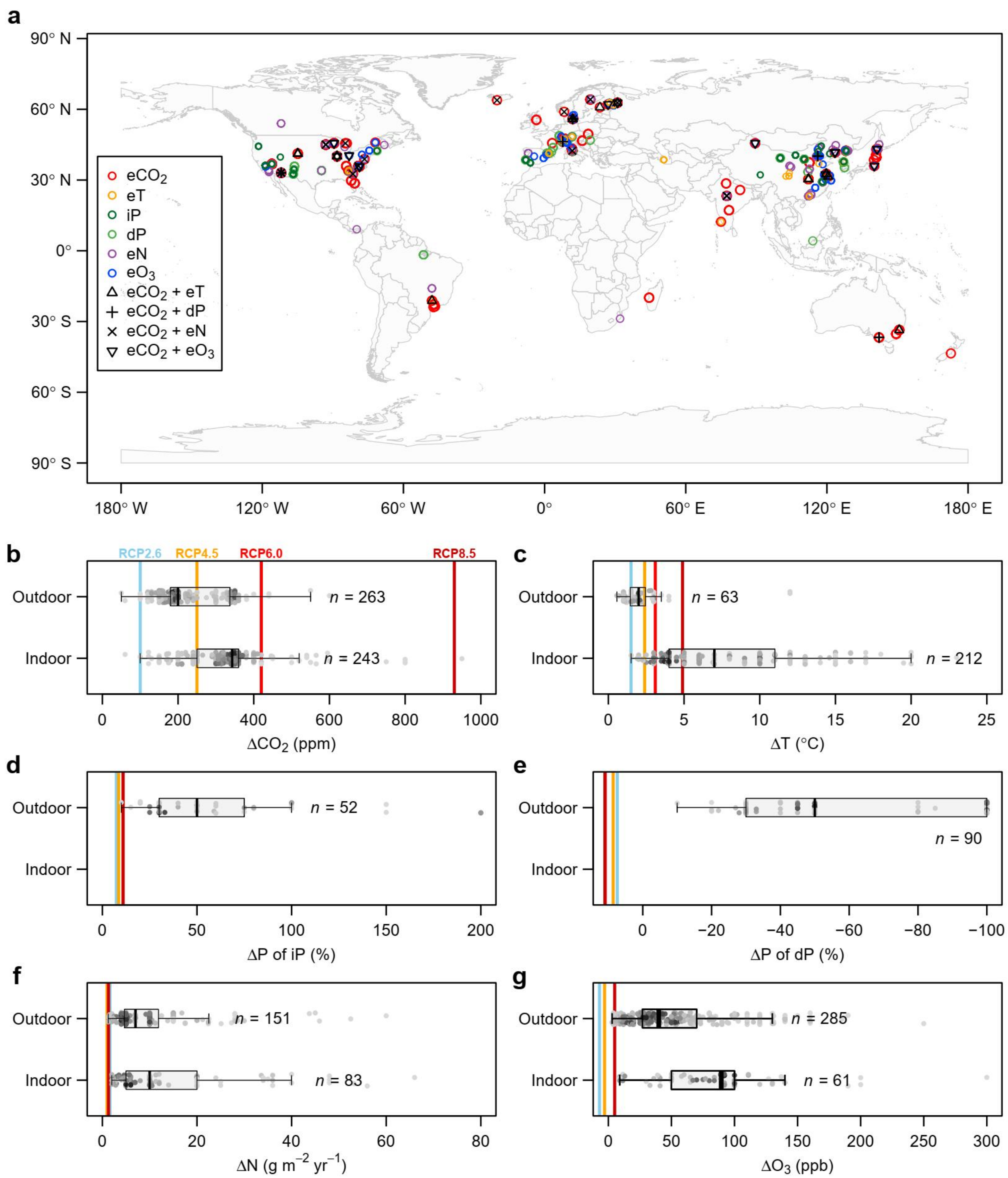

Figure S1 | Global distribution of study sites included in this meta-analysis and the magnitudes of experiments against model projections. a, The global distribution of study sites of single- and combined factor experiments, only outdoor experiments are shown. **b,** The increases in atmospheric CO_2 concentrations manipulated by eCO2 experiments and projected in all IPCC Representative Concentration Pathways (RCPs). **c,** The increases of Earth's land surface temperature manipulated by

eT experiments and projected in all RCPs. **d-e,** The changes in precipitation manipulated by precipitation experiments and projected in all RCPs. **f,** The changes in atmospheric nitrogen deposition manipulated by eN experiments and projected in different RCPs. **g,** The changes in atmospheric O_3 concentrations of eO_3 experiments and projected in different RCPs. In the box plots **b-g**, the centre line represents the median, the edges of the box indicate the 25th and 75th percentiles, and the whiskers indicate 1.5 × IQR (the interquartile range between the 25th and 75th percentiles).

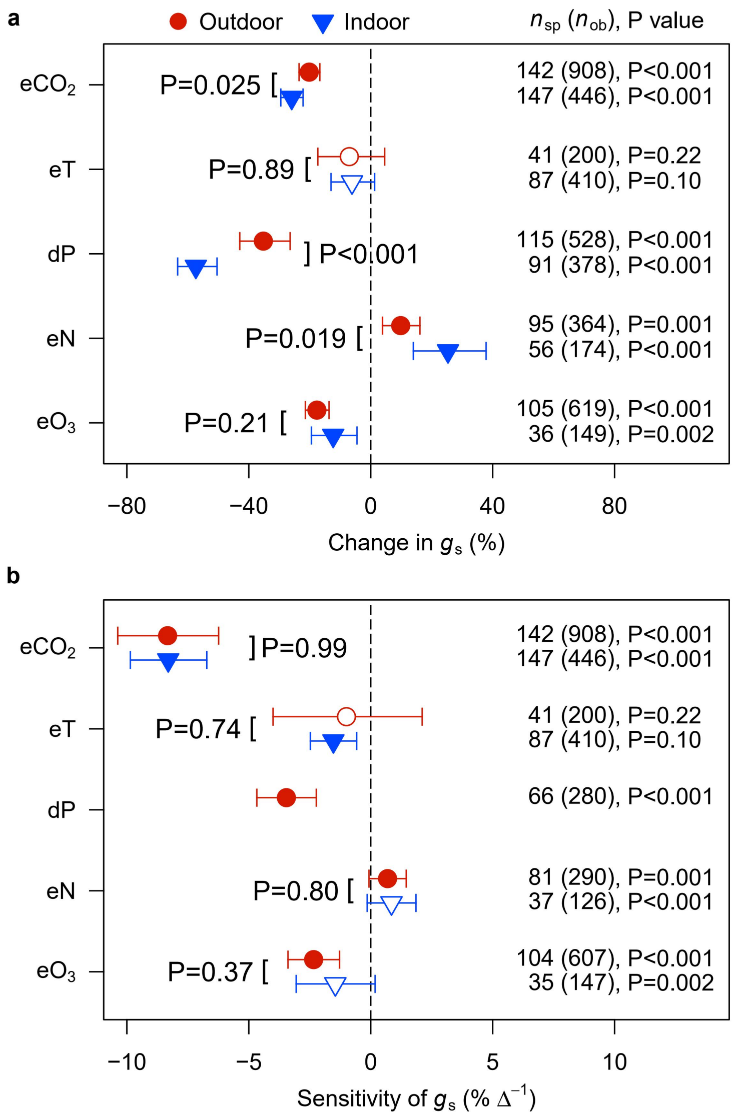

Figure S2 | Responses of g_s to global change factors in the outdoor against indoor experiments. **a,** The overall changes of g_s in response to global change factors. **b,** The g_s sensitivities in response to global change factors. The error bars represent the 95% confidence interval of the weighted means. Open and filled symbols represent insignificant ($P > 0.05$) and significant ($P < 0.05$) differences between the g_s response and zero, respectively. P values near the error bars indicate statistic significant levels for comparisons between indoor and outdoor groups. The numbers outside and inside parentheses represent the number of species (n_{sp}) and observations (n_{ob}), respectively.

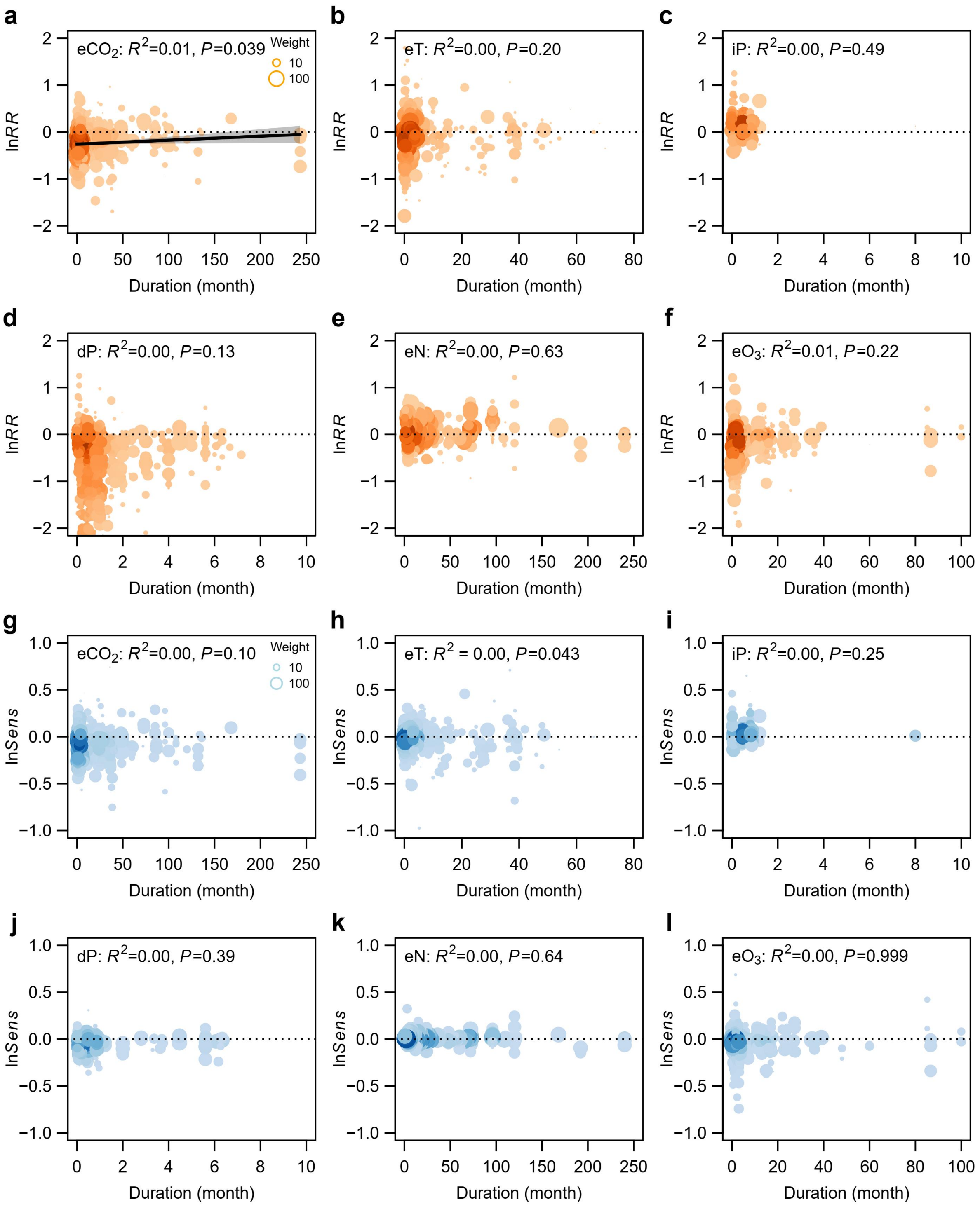

Figure S3 | Stomatal responses to global change factors in relation to experimental duration. ln*RR* and ln*Sens* represent natural log-transformed response ratio and sensitivity, respectively. The size of each point represents the adjusted weight of each data point, and the darker the color means the higher the point density. The error bands surrounding the regression lines represent the 95% confidence interval.

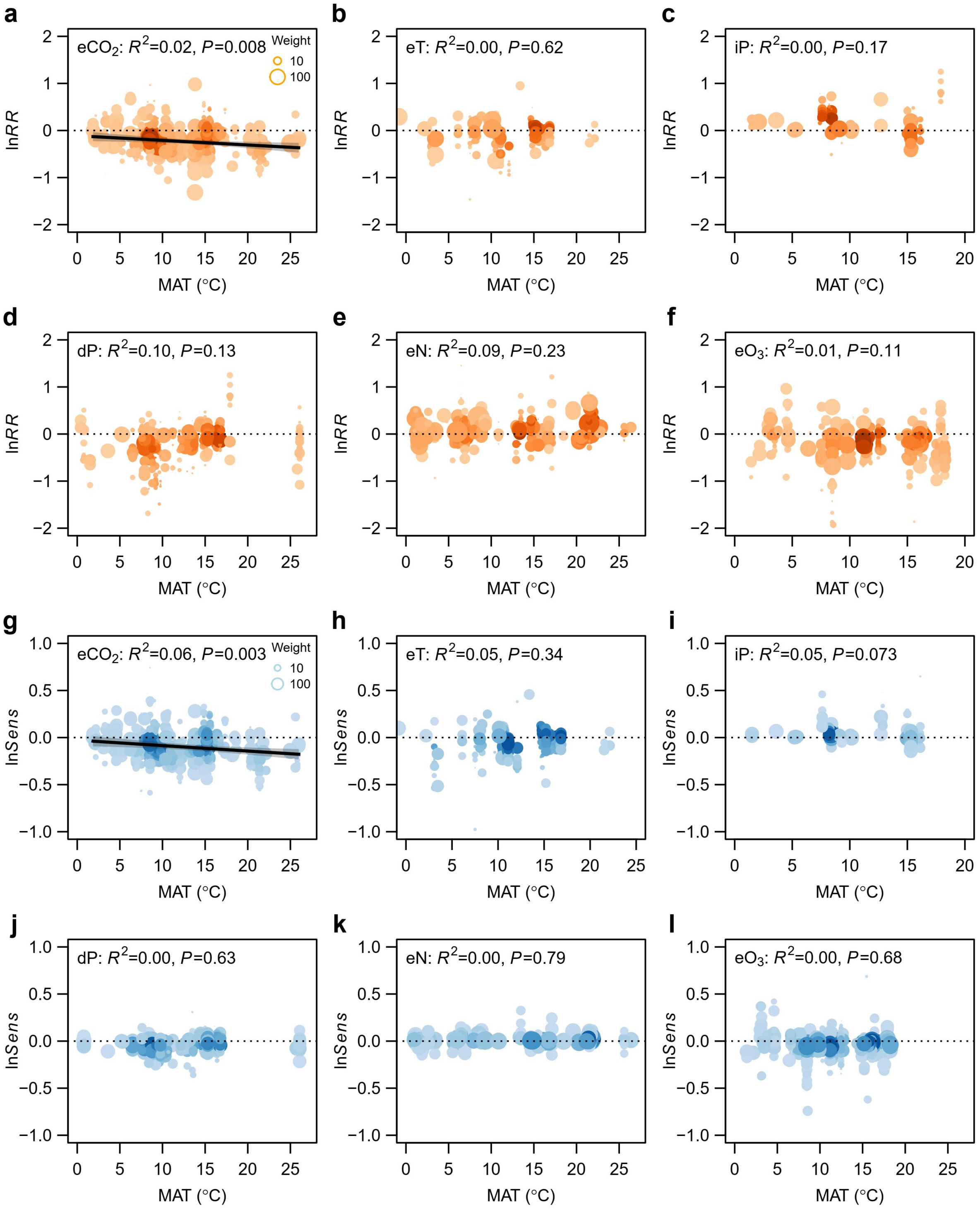

Figure S4 | Stomatal responses to global change factors in relation to mean annual temperature (MAT). ln*RR* and ln*Sens* represent natural log-transformed response ratio and sensitivity, respectively. The size of each point represents the adjusted weight of each data point, and the darker the color means the higher the point density. The error bands surrounding the regression lines represent the 95% confidence interval.

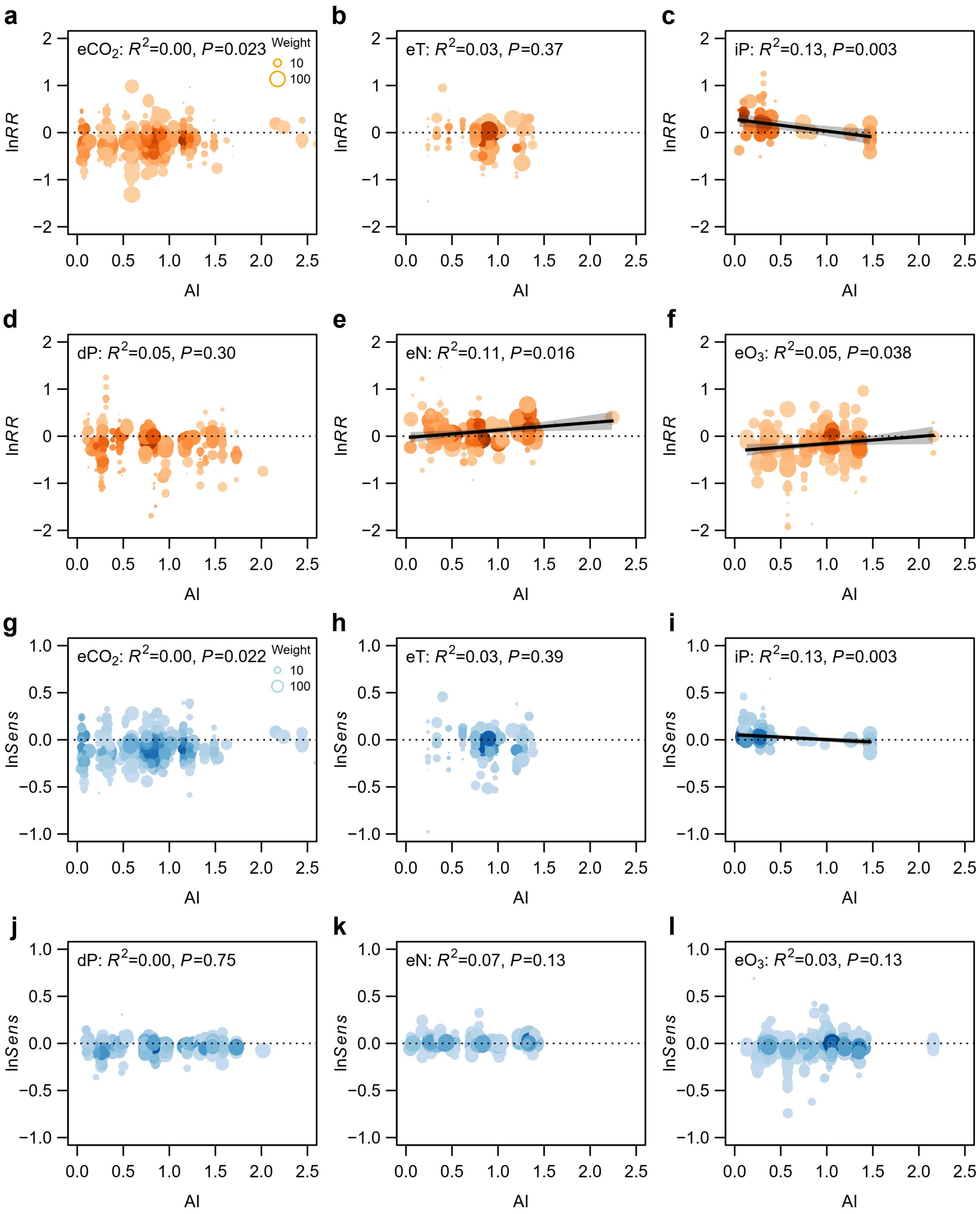

Figure S5 | Stomatal responses to global change factors in relation to aridity index (AI). ln*RR* and ln*Sens* represent natural log-transformed response ratio and sensitivity, respectively. The size of each point represents the adjusted weight of each data point, and the darker the color means the higher the point density. The error bands surrounding the regression lines represent the 95% confidence interval.

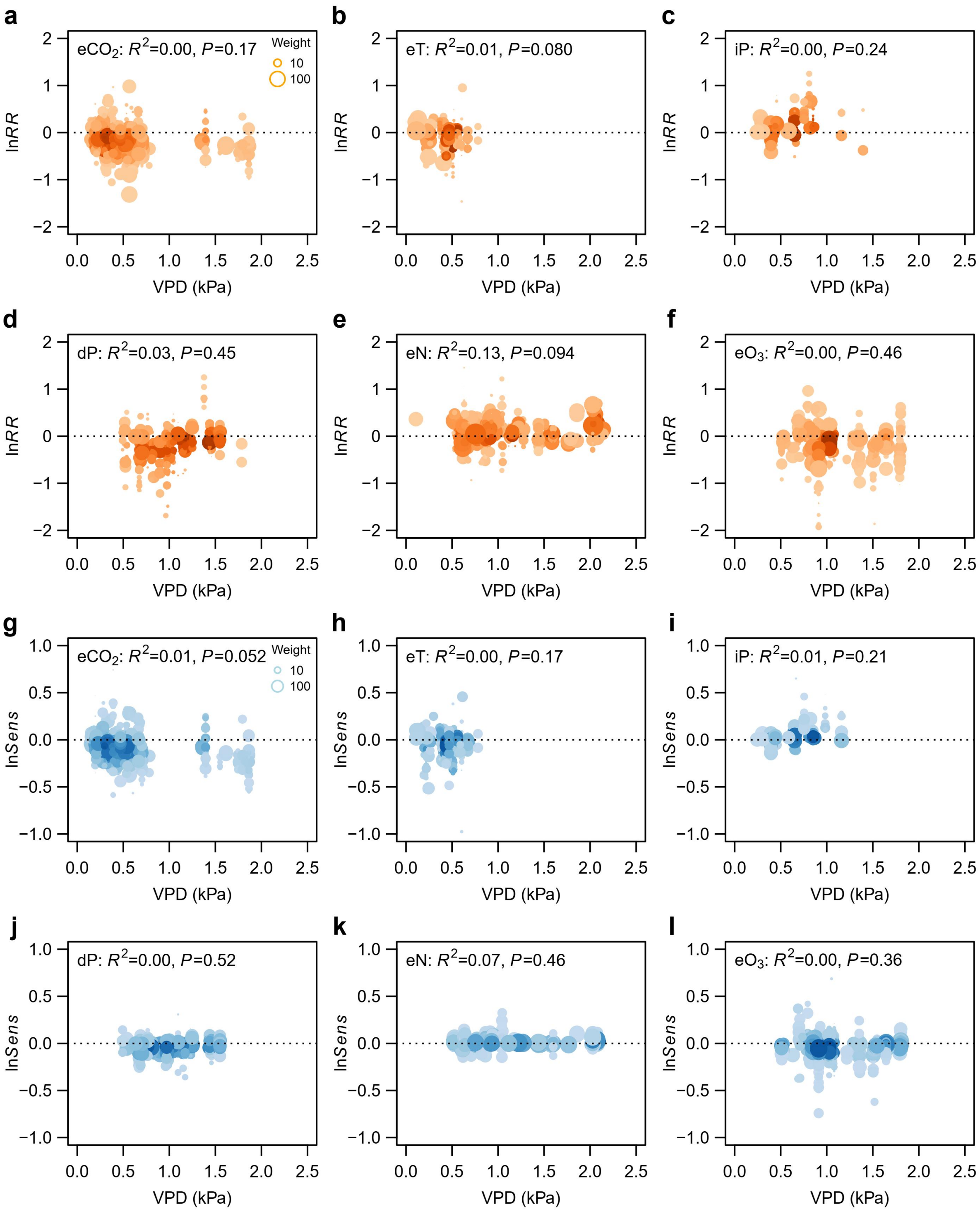

Figure S6 | Stomatal responses to global change factors in relation to vapor pressure difference (VPD). ln*RR* and ln*Sens* represent natural log-transformed response ratio and sensitivity, respectively. The size of each point represents the adjusted weight of each data point, and the darker the color means the higher the point density.

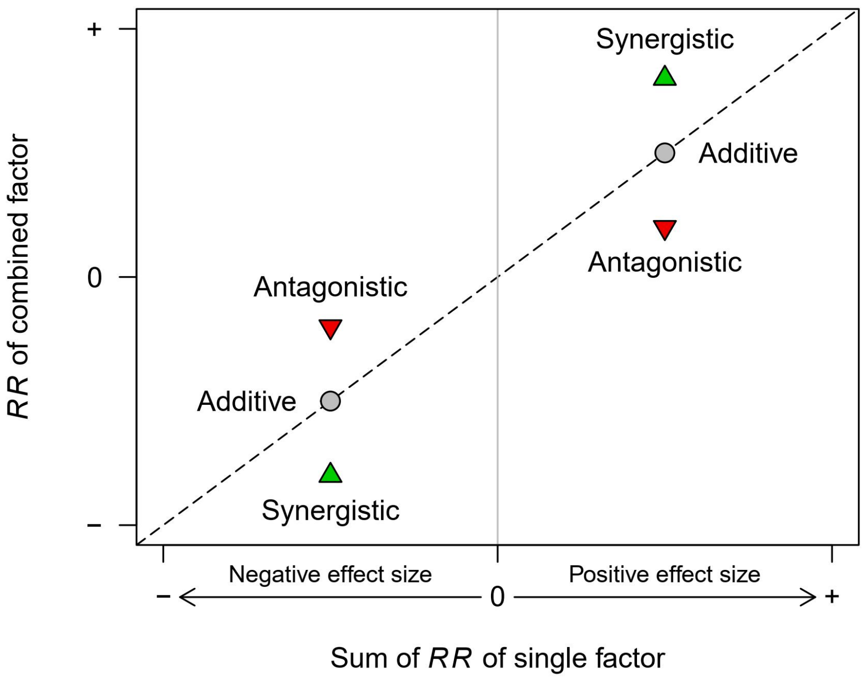

Figure S7 | The location of points that represent different interactive effects on the plot. Dashed line indicates 1:1 relation.

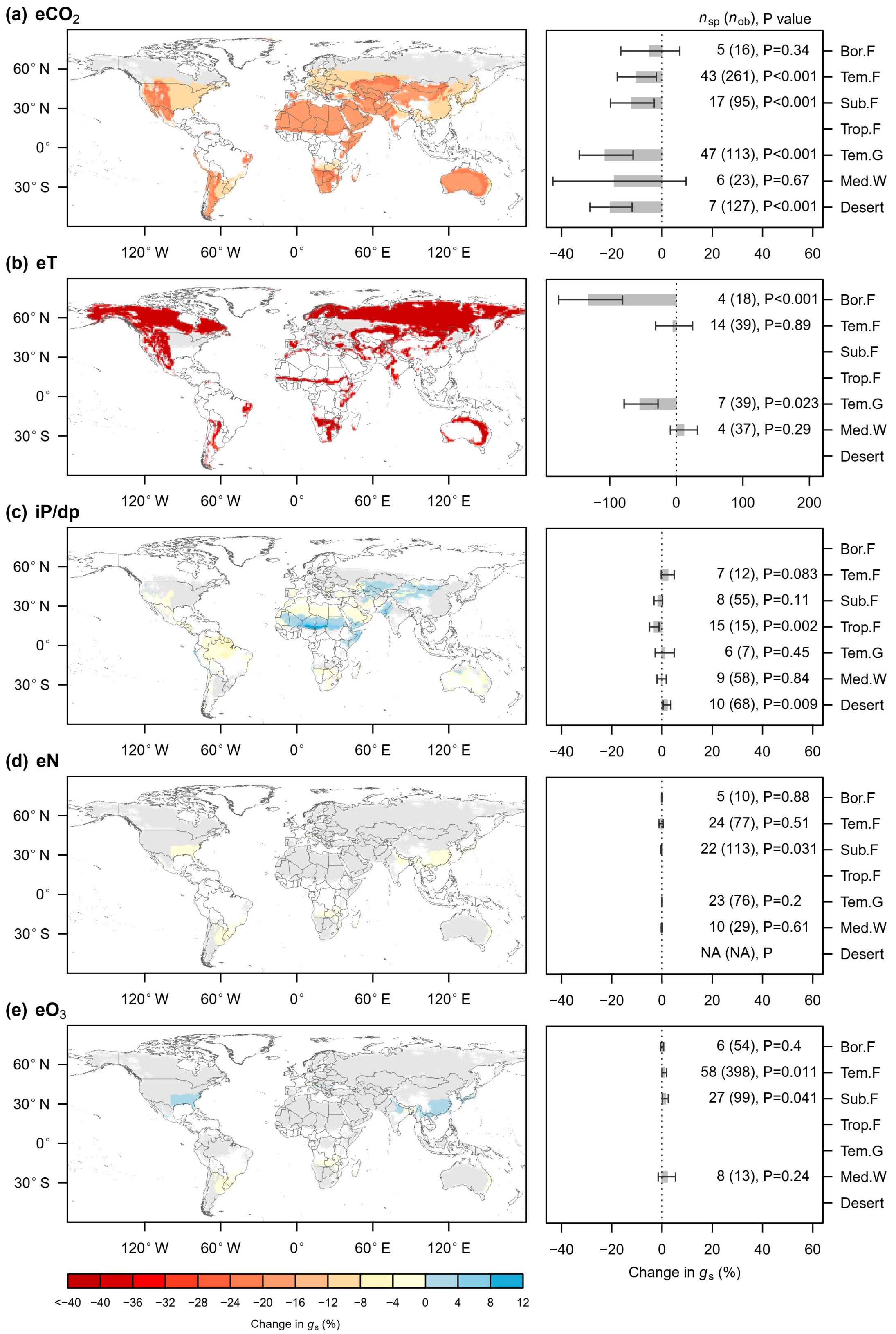

Figure S8 | Predicted changes in g_s across biomes by the end of the twenty-first century under SSP2-4.5/RCP4.5 scenario. The left panels display global maps depicting changes in g_s, with grey

and white land colors indicating areas where changes are statistically insignificant and where is a lack of data, respectively. The right panels depict biome-level predictions, with error bars representing the 95% confidence interval. The numbers outside and inside parentheses represent the number of species (n_{sp}) and observations (n_{ob}), respectively. Bor.F: boreal forest, Tem.F: temperate forest, Sub.F: subtropical forest, Trop.F: tropical forest, Tem.G: temperate grassland, Med.W: Mediterranean woodland.

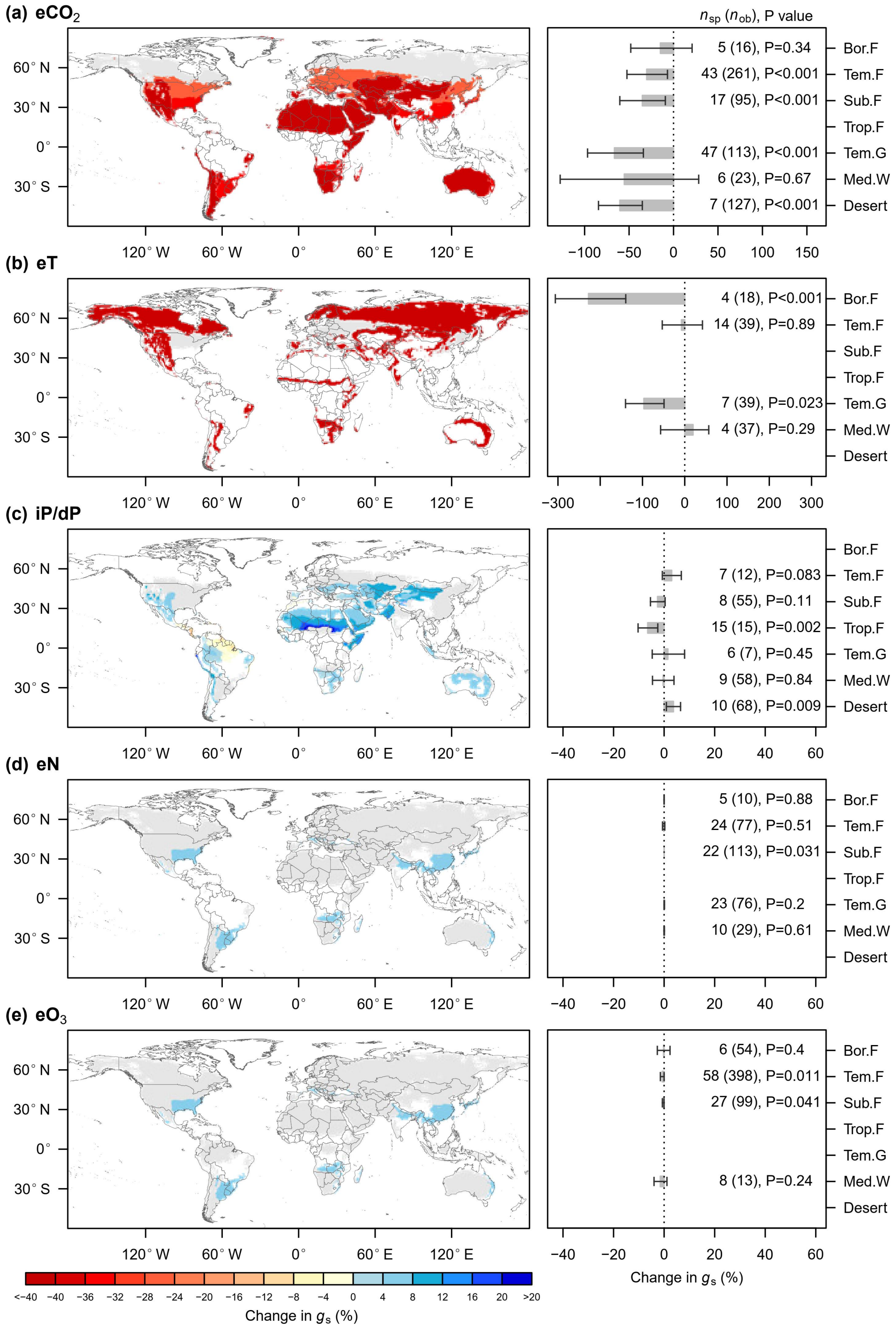

Figure S9 | Predicted changes in g_s across biomes by the end of the twenty-first century under SSP5-8.5/RCP8.5 scenario. The left panels display global maps depicting changes in g_s, with grey

and white land colors indicating areas where changes are statistically insignificant and where is a lack of data, respectively. The right panels depict biome-level predictions, with error bars representing the 95% confidence interval. The numbers outside and inside parentheses represent the number of species (n_{sp}) and observations (n_{ob}), respectively. Bor.F: boreal forest, Tem.F: temperate forest, Sub.F: subtropical forest, Trop.F: tropical forest, Tem.G: temperate grassland, Med.W: Mediterranean woodland.

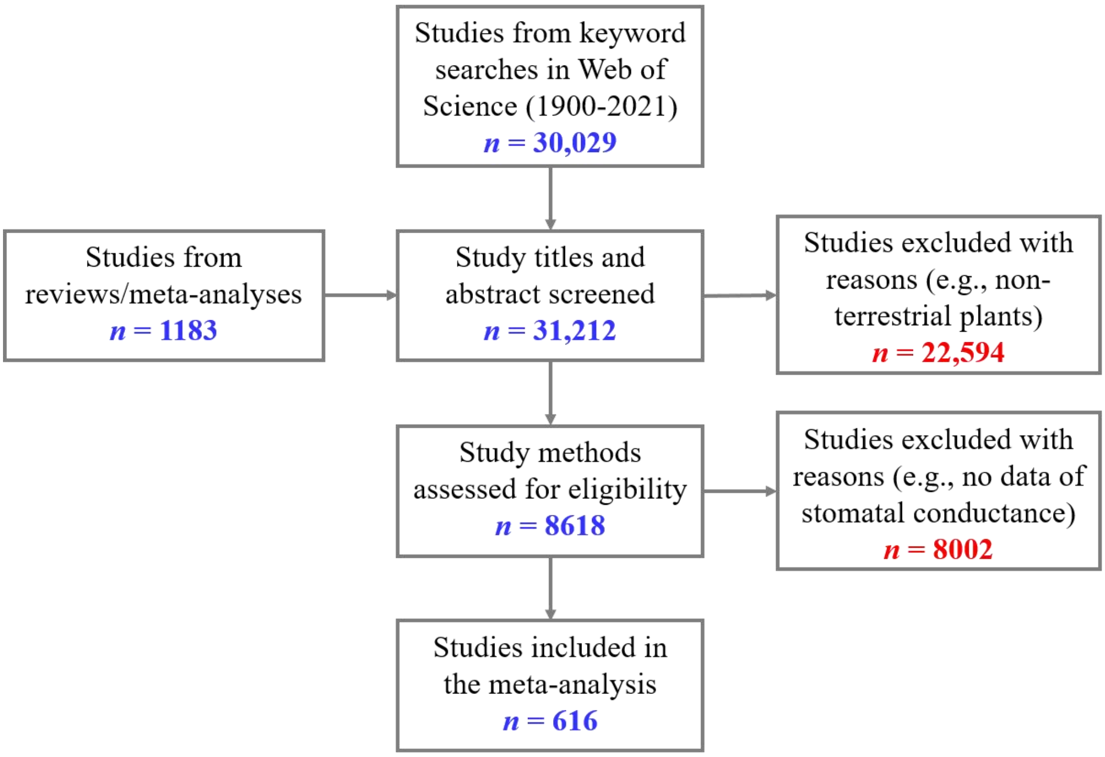

Figure S10 | PRISMA flow diagram depicting the literature search and study selection process.

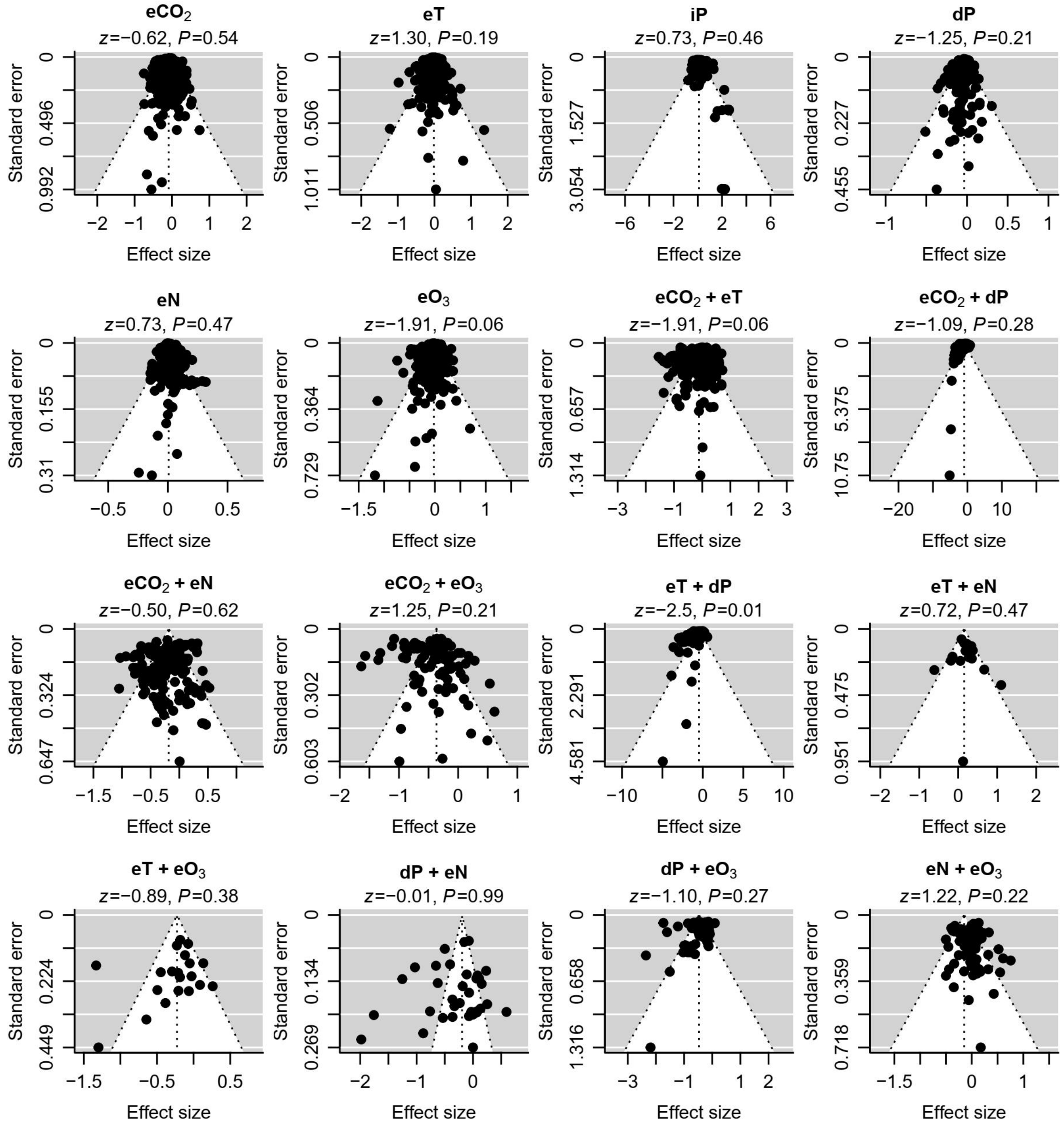

Figure S11 | Funnel plots for assessments of publication bias. Results of Egger's regression are given at the top to each panel, with *P* values > 0.05 indicate no publication bias.

Supplementary References

1. Abdelhakim LOA, Palma CFF, Zhou R, Wollenweber B, Ottosen CO, Rosenqvist E. The effect of individual and combined drought and heat stress under elevated CO_2 on physiological responses in spring wheat genotypes. *Plant Physiology and Biochemistry* **162**, 301-314 (2021).

2. Adachi M*, et al.* Soil and water warming accelerates phenology and down-regulation of leaf photosynthesis of rice plants grown under free-air CO_2 enrichment (FACE). *Plant and Cell Physiology* **55**, 370-380 (2014).

3. Adam NR, Wall GW, Kimball BA, Idso SB, Webber AN. Photosynthetic down-regulation over long-term CO_2 enrichment in leaves of sour orange (*Citrus aurantium*) trees. *New Phytologist* **163**, 341-347 (2004).

4. Agrawal M, Deepak SS. Physiological and biochemical responses of two cultivars of wheat to elevated levels of CO_2 and SO_2, singly and in combination. *Environmental Pollution* **121**, 189-197 (2003).

5. Ainsworth EA, Rogers A, Blum H, Nösberger J, Long SP. Variation in acclimation of photosynthesis in *Trifolium repens* after eight years of exposure to free air CO_2 enrichment (FACE). *Journal of Experimental Botany* **54**, 2769-2774 (2003).

6. Albert KR, Ro-poulsen H, Mikkelsen TN, Michelsen A, Van der Linden L, Beier C. Effects of elevated CO_2, warming and drought episodes on plant carbon uptake in a temperate heath ecosystem are controlled by soil water status. *Plant, Cell and Environment* **34**, 1207-1222 (2011).

7. Albert KR, Ro-Poulsen H, Mikkelsen TN, Michelsen A, van der Linden L, Beier C. Interactive effects of elevated CO_2, warming, and drought on photosynthesis of *Deschampsia flexuosa* in a temperate heath ecosystem. *Journal of Experimental Botany* **62**, 4253-4266 (2011).

8. Ambebe TF, Dang Q. Low moisture availability inhibits the enhancing effect of increased soil temperature on net photosynthesis of white birch (*Betula papyrifera*) seedlings grown under ambient and elevated carbon dioxide concentrations. *Tree Physiology* **29**, 1341-1348 (2009).

9. An J*, et al.* Physiological and growth responses to experimental warming in first-year seedlings of deciduous tree species. *Turkish Journal of Agriculture and Forestry* **41**, 175-182 (2017).

10. Anderson LJ, Cipollini D. Gas exchange, growth, and defense responses of invasive *Alliaria petiolata* (Brassicaceae) and native *Geum vernum* (Rosaceae) to elevated atmospheric CO_2 and warm spring temperatures. *American Journal of Botany* **100**, 1544-1554 (2013).

11. Anderson PD, Houpis JLJ, Helms JA, Momen B. Seasonal variation of gas exchange and pigmentation in branches of three grafted clones of mature *Ponderosa pine* exposed to ozone and acid rain. *Environmental Pollution* **97**, 253-263 (1997).

12. Anderson PD, Tomlinson PT. Ontogeny affects response of northern red oak seedlings to

elevated CO_2 and water stress - I. carbon assimilation and biomass production. *New Phytologist* **140**, 477-491 (1998).

13. Apple ME, Olszyk DM, Ormrod DP, Lewis A, Southworth D, Tingey DT. Morphology and stomatal function of douglas fir needles exposed to climate chance: elevated CO_2 and temperature. *International Journal of Plant Sciences* **161**, 127-132 (2000).
14. Aranda I, Cadahia E, de Simon BF. Leaf ecophysiological and metabolic response in *Quercus pyrenaica* Willd seedlings to moderate drought under enriched CO_2 atmosphere. *J Plant Physiol* **244**, 153083 (2020).
15. Aranda X, Agusti C, Joffre R, Fleck I. Photosynthesis, growth and structural characteristics of holm oak resprouts originated from plants grown under elevated CO_2. *Physiologia Plantarum* **128**, 302-312 (2006).
16. Aranjuelo I, Irigoyen JJ, Nogues S, Sanchez-Diaz M. Elevated CO_2 and water-availability effect on gas exchange and nodule development in N_2-fixing alfalfa plants. *Environmental and Experimental Botany* **65**, 18-26 (2009).
17. Aranjuelo I, Irigoyen JJ, Perez P, Martinez-Carrasco R, Sanchez-diaz M. Response of nodulated alfalfa to water supply, temperature and elevated CO_2: productivity and water relations. *Environmental and Experimental Botany* **55**, 130-141 (2006).
18. Aranjuelo I*, et al.* The response of nodulated alfalfa to water supply, temperature and elevated CO_2: photosynthetic downregulation. *Physiologia Plantarum* **123**, 348-358 (2005).
19. Avila RT*, et al.* Coffee plants respond to drought and elevated [CO_2] through changes in stomatal function, plant hydraulic conductance, and aquaporin expression. *Environmental and Experimental Botany* **177**, (2020).
20. Bachofen C, Moser B, Hoch G, Ghazoul J, Wohlgemuth T. No carbon "bet hedging" in pine seedlings under prolonged summer drought and elevated CO_2. *Journal of Ecology* **106**, 31-46 (2018).
21. Back J, Vanderklein DW, Topa MA. Effects of elevated ozone on CO_2 uptake and leaf structure in sugar maple under two light environments. *Plant, Cell and Environment* **22**, 137-147 (1999).
22. Balaguer L, Barnes JD, Panicucci A, Borland AM. Production and utilization of assimilates in wheat (*Triticum-aestivum* L.) leaves exposed to elevated O_3 and/or CO_2. *New Phytologist* **129**, 557-568 (1995).
23. Barker DH, Loveys BR, Egerton JJG, Gorton H, Williams WE, Ball MC. CO_2 enrichment predisposes foliage of a eucalypt to freezing injury and reduces spring growth. *Plant, Cell and Environment* **28**, 1506-1515 (2005).
24. Barker DH*, et al.* Enhanced monsoon precipitation and nitrogen deposition affect leaf traits and photosynthesis differently in spring and summer in the desert shrub *Larrea tridentata*. *New Phytologist* **169**, 799-808 (2006).

25. Bartkowiak SM, Samuelson LJ, McGuire MA, Teskey RO. Fertilization increases sensitivity of canopy stomatal conductance and transpiration to throughfall reduction in an 8-year-old loblolly pine plantation. *Forest Ecology and Management* **354**, 87-96 (2015).

26. Barton CVM, Jarvis PG. Growth response of branches of *Picea sitchensis* to four years exposure to elevated atmospheric carbon dioxide concentration. *New Phytologist* **144**, 233-243 (1999).

27. Bassow SL, McConnaughay KDM, Bazzaz FA. The response of temperate tree seedlings grown in elevated CO_2 to extreme temperature events. *Ecological Applications* **4**, 593-603 (1994).

28. Baudis M*, et al.* Intraspecific differences in responses to rainshelter-induced drought and competition of *Fagus sylvatica* L. across Germany. *Forest Ecology and Management* **330**, 283-293 (2014).

29. Bauweraerts I, Ameye M, Wertin TM, McGuire MA, Teskey RO, Steppe K. Acclimation effects of heat waves and elevated CO_2 on gas exchange and chlorophyll fluorescence of northern red oak (*Quercus rubra* L.) seedlings. *Plant Ecology* **215**, 733-746 (2014).

30. Bauweraerts I, Mannaerts TBHL, Wertin TM, McGuire MA, Teskey RO, Steppe K. Elevated CO_2 and growth temperature have a small positive effect on photosynthetic thermotolerance of *Pinus taeda* seedlings. *Trees* **28**, 1515-1526 (2014).

31. Bauweraerts I, Wertin TM, Ameye M, McGuire MA, Teskey RO, Steppe K. The effect of heat waves, elevated [CO_2] and low soil water availability on northern red oak (*Quercus rubra* L.) seedlings. *Global Change Biology* **19**, 517-528 (2013).

32. Bernacchi CJ*, et al.* Photosynthesis and stomatal conductance responses of poplars to free-air CO_2 enrichment (PopFACE) during the first growth cycle and immediately following coppice. *New Phytologist* **159**, 609-621 (2003).

33. Bernacchi CJ*, et al.* Hourly and seasonal variation in photosynthesis and stomatal conductance of soybean grown at future CO_2 and ozone concentrations for 3 years under fully open-air field conditions. *Plant, Cell and Environment* **29**, 2077-2090 (2006).

34. Berryman CA, Eamus D, Duff GA. Stomatal responses to a range of variables in two tropical tree species grown with CO_2 enrichment. *Journal of Experimental Botany* **45**, 539-546 (1994).

35. Besson CK*, et al.* Cork oak physiological responses to manipulated water availability in a Mediterranean woodland. *Agricultural and Forest Meteorology* **184**, 230-242 (2014).

36. Bethers S, Day ME, Bruce Wiersma G, Fernandez IJ, Alexander Elvir J. Effects of chronically elevated nitrogen and sulfur deposition on sugar maple saplings: nutrition, growth and physiology. *Forest Ecology and Management* **258**, 895-902 (2009).

37. Bettarini I, Vaccari FP, Miglietta F. Elevated CO_2 concentrations and stomatal density: observations from 17 plant species growing in a CO_2 spring in central Italy. *Global Change Biology* **4**, 17-22 (1998).

38. Beyers JL, Riechers GH, Temple PJ. Effects of long-term ozone exposure and drought on the

photosynthetic capacity of ponderosa pine (*Pinus ponderosa* L.). *New Phytologist* **122**, 81-90 (1992).

39. Bhattacharya NC*, et al.* Leaf water relations of cotton in a free-air CO_2-enriched environment. *Agricultural and Forest Meteorology* **70**, 171-182 (1994).

40. Bhusal N, Lee M, Han AR, Han A, Kim HS. Responses to drought stress in *Prunus sargentii* and *Larix kaempferi* seedlings using morphological and physiological parameters. *Forest Ecology and Management* **465**, (2020).

41. Bi Y, Qin J, Hu Y, Liu Q. Effects of elevated carbon dioxide concentration on photosynthetic characteristics ana growth of *Cinnamomum camphora* seedlings. *Journal of Northeast Forestry University* **39**, 27-29 (2011).

42. Bian F, Sun Y, Niu Y, Du Y, Zhai H. Effect of root-applied melatonin on photosystem II in grape leaves under heat stress. *Plant Physiology Journal* **53**, 257-263 (2017).

43. Birami B*, et al.* Hot drought reduces the effects of elevated CO_2 on tree water-use efficiency and carbon metabolism. *New Phytologist* **226**, 1607-1621 (2020).

44. Bista DR, Heckathorn SA, Jayawardena DM, Boldt JK. Effect of drought and carbon dioxide on nutrient uptake and levels of nutrient-uptake proteins in roots of barley. *American Journal of Botany* **107**, 1401-1409 (2020).

45. Biswas DK, Xu H, Li Y, Ma B, Jiang G. Modification of photosynthesis and growth responses to elevated CO_2 by ozone in two cultivars of winter wheat with different years of release. *Journal of Experimental Botany* **64**, 1485-1496 (2013).

46. Blackman CJ, Aspinwall MJ, Resco de Dios V, Smith RA, Tissue DT. Leaf photosynthetic, economics and hydraulic traits are decoupled among genotypes of a widespread species of *Eucalypt* grown under ambient and elevated CO_2. *Functional Ecology* **30**, 1491-1500 (2016).

47. Blumenthal DM*, et al.* Invasive forb benefits from water savings by native plants and carbon fertilization under elevated CO_2 and warming. *New Phytologist* **200**, 1156-1165 (2013).

48. Bobich EG, Barron-Gafford GA, Rascher KG, Murthy R. Effects of drought and changes in vapour pressure deficit on water relations of *Populus deltoides* growing in ambient and elevated CO_2. *Tree Physiology* **30**, 866-875 (2010).

49. Bollig C, Feller U. Impacts of drought stress on water relations and carbon assimilation in grassland species at different altitudes. *Agriculture, Ecosystems and Environment* **188**, 212-220 (2014).

50. Booker FL, Burkey KO, Pursley WA, Heagle AS. Elevated carbon dioxide and ozone effects on peanut: I. gas-exchange, biomass, and leaf chemistry. *Crop Science* **47**, 1475-1487 (2007).

51. Booker FL, Fiscus EL. The role of ozone flux and antioxidants in the suppression of ozone injury by elevated CO_2 in soybean. *Journal of Experimental Botany* **56**, 2139-2151 (2005).

52. Borjigidai A, Hikosaka K, Hirose T, Hasegawa T, Okada M, Kobayashi K. Seasonal changes in temperature dependence of photosynthetic rate in rice under a free-air CO_2 enrichment. *Annals of Botany* **97**, 549-557 (2006).

53. Bosac C, Gardner SDL, Taylor G, Wilkins D. Elevated CO_2 and hybrid poplar: a detailed investigation of root and shoot growth and physiology of *Populus-euramericana*. *Forest Ecology and Management* **74**, 103-116 (1995).

54. Bowman WD, Theodose TA, Fisk MC. Physiological and production responses of plant growth forms to increases in limiting resources in alpine tundra: implications for differential community response to environmental change. *Oecologia* **101**, 217-227 (1995).

55. Brilli F, Tricoli D, Fares S, Centritto M, Loreto F. The use of branch enclosures to assess direct and indirect effects of elevated CO_2 on photosynthesis, respiration and isoprene emission of *Populus alba* leaves. *Iforest-Biogeosciences and Forestry* **1**, 49-54 (2008).

56. Broadmeadow MSJ, Heath J, Randle TJ. Environmental limitations to O_3 uptake - some key results from young trees growing at elevated CO_2 concentrations. *Water Air and Soil Pollution* **116**, 299-310 (1999).

57. Broughton KJ*, et al.* The effect of elevated atmospheric CO_2 and increased temperatures on an older and modern cotton cultivar. *Functional Plant Biology* **44**, 1207-1218 (2017).

58. Broughton KJ*, et al.* Warming alters the positive impact of elevated CO_2 concentration on cotton growth and physiology during soil water deficit. *Functional Plant Biology* **44**, 267-278 (2017).

59. Bryant J, Taylor G, Frehner M. Photosynthetic acclimation to elevated CO_2 is modified by source : sink balance in three component species of chalk grassland swards grown in a free air carbon dioxide enrichment (FACE) experiment. *Plant, Cell and Environment* **21**, 159-168 (1998).

60. Bucci SJ*, et al.* Nutrient availability constrains the hydraulic architecture and water relations of savannah trees. *Plant, Cell and Environment* **29**, 2153-2167 (2006).

61. Bucher-Wallin IK*, et al.* Effects of elevated CO_2, increased nitrogen deposition and soil on evapotranspiration and water use efficiency of spruce-beech model ecosystems. *Phyton-Annales Rei Botanicae* **40**, 49-60 (2000).

62. Bunce JA. Stomatal conductance, photosynthesis and respiration of temperate deciduous tree seedlings grown outdoors at an elevated concentration of carbon-dioxide. *Plant Cell and Environment* **15**, 541-549 (1992).

63. Cai C*, et al.* Do all leaf photosynthesis parameters of rice acclimate to elevated CO_2, elevated temperature, and their combination, in FACE environments? *Global Change Biology* **24**, 1685-1707 (2017).

64. Calatayud V, Cervero J, Sanz MJ. Foliar, physiologial and growth responses of four maple species exposed to ozone. *Water Air and Soil Pollution* **185**, 239-254 (2007).

65. Calcerrada RJ, Letts MG, Rolo V, Roset S, Rambal S. Multiyear impacts of partial throughfall exclusion on *Buxus sempervirens* in a Mediterranean forest. *Forest Systems* **22**, 202-213 (2013).

66. Calfapietra C, Mugnozza GS, Karnosky DF, Loreto F, Sharkey TD. Isoprene emission rates under elevated CO_2 and O_3 in two field-grown aspen clones differing in their sensitivity to O_3. *New Phytologist* **179**, 55-61 (2008).

67. Calfapietra C*, et al.* Canopy profiles of photosynthetic parameters under elevated CO_2 and N fertilization in a poplar plantation. *Environmental pollution* **137**, 525-535 (2005).

68. Calfapietra C*, et al.* Isoprene synthase expression and protein levels are reduced under elevated O_3 but not under elevated CO_2 (FACE) in field-grown aspen trees. *Plant, Cell and Environment* **30**, 654-661 (2007).

69. Campbell DR, Wu CA, Travers SE. Photosynthetic and growth response of reciprocal hybrids to variation in water and nitrogen availability. *American journal of botany* **97**, 925-933 (2010).

70. Cao B, Dang Q, Zhang S. Relationship between photosynthesis and leaf nitrogen concentration in ambient and elevated [CO_2] in white birch seedlings. *Tree Physiology* **27**, 891-899 (2007).

71. Cao J*, et al.* Characteristics of photosynthesis in wheat cultivars with different sensitivi-ties to ozone under O_3 free air concentration enrichment conditions. *Acta Agronomica Sinica* **35**, 1500-1507 (2009).

72. Centritto M, Brilli F, Fodale R, Loreto F. Different sensitivity of isoprene emission, respiration and photosynthesis to high growth temperature coupled with drought stress in black poplar (*Populus nigra*) saplings. *Tree Physiology* **31**, 275-286 (2010).

73. Centritto M, Lee HSJ, Jarvis PG. Interactive effects of elevated [CO_2] and drought on cherry (*Prunus avium*) seedlings I. gowth, whole-plant water use efficiency and water loss. *New Phytologist* **141**, 129-140 (1999).

74. Centritto M, Lucas ME, Jarvis PG. Gas exchange, biomass, whole-plant water-use efficiency and water uptake of peach (*Prunus persica*) seedlings in response to elevated carbon dioxide concentration and water availability. *Tree Physiology* **22**, 699-706 (2002).

75. Chang H, An J, Roh Y, Son Y. Experimental warming and drought treatments reduce physiological activities and increase mortality of *Pinus koraiensis* seedlings. *Plant Ecology* **221**, 515-527 (2020).

76. Chaves MM, Pereira JS, Cerasoli S, CliftonBrown J, Miglietta F, Raschi A. Leaf metabolism during summer drought in *Quercus ilex* trees with lifetime exposure to elevated CO_2. *Journal of Biogeography* **22**, 255-259 (1995).

77. Chen G*, et al.* Leaf photosynthetic acclimation of *Echinochloa crusgalli* grown in rice field to free-air CO_2 enrichment (FACE). *Chinese Journal of Applied Ecology* **13**, 1201-1204 (2002).

78. Chen G*, et al.* Photosynthetic acclimation in rice leaves to free-air CO_2 enrichment related to both ribulose-1,5-bisphosphate carboxylation limitation and ribulose-1,5-bisphosphate

regeneration limitation. *Plant and Cell Physiology* **46**, 1036-1045 (2005).

79. Chen S, Bai Y, Zhang L, Han X. Comparing physiological responses of two dominant grass species to nitrogen addition in Xilin river basin of China. *Environmental and Experimental Botany* **53**, 65-75 (2005).

80. Chen Y, Yu J, Huang B. Effects of elevated CO_2 concentration on water relations and photosynthetic responses to drought stress and recovery during rewatering in Tall Fescue. *Journal of the American Society for Horticultural Science* **140**, 19-26 (2015).

81. Chen Z, Lu H, Liu S, Liu X, Liu C, Wan X. Responses to drought stress in *Prunus sargentii* and *Larix kaempferi* seedlings using morphological and physiological parameters. *Acta Ecologica Sinica* **38**, 2405-2413 (2018).

82. Chen Z, Lu H, Liu X, Liu C, Liu S, Wan X. Ecophysiological response of *Lindera obtusiloba* to rainfall reduction in Baotianman nature reserve. *Forest Research* **30**, 430-435 (2017).

83. Clark CS, Weber JA, Lee EH, Hogsett WE. Accentuation of gas exchange gradients in flushes of *Ponderosa pine* exposed to ozone. *Tree Physiology* **15**, 181-189 (1995).

84. Clifford SC, Stronach IM, Black CR, Singleton-Jones PR, Azam-Ali SN, Crout NMJ. Effects of elevated CO_2, drought and temperature on the water relations and gas exchange of groundnut (*Arachis hypogaea*) stands grown in controlled environment glasshouses. *Physiologia Plantarum* **110**, 78-88 (2000).

85. Coleman MD, Isebrands JG, Dickson RE, Karnosky DF. Photosynthetic productivity of aspen clones varying in sensitivity to tropospheric ozone. *Tree Physiology* **15**, 585-592 (1995).

86. Constable JVH, Litvak ME, Greenberg JP, Monson RK. Monoterpene emission from coniferous trees in response to elevated CO_2 concentration and climate warming. *Global Change Biology* **5**, 252-267 (1999).

87. Contran N, Günthardt-Goerg MS, Kuster TM, Cerana R, Crosti P, Paoletti E. Physiological and biochemical responses of *Quercus pubescens* to air warming and drought on acidic and calcareous soils. *Plant Biology* **15**, 157-168 (2013).

88. Cowling SA, Sage RF. Interactive effects of low atmospheric CO_2 and elevated temperature on growth, photosynthesis and respiration in *Phaseolus vulgaris*. *Plant, Cell and Environment* **21**, 427-435 (1998).

89. Crous KY*, et al.* Photosynthesis of temperate *Eucalyptus globulus* trees outside their native range has limited adjustment to elevated CO_2 and climate warming. *Global Change Biology* **19**, 3790-3807 (2013).

90. Cruz JL, Alves AAC, LeCain DR, Ellis DD, Morgan JA. Elevated CO_2 concentrations alleviate the inhibitory effect of drought on physiology and growth of cassava plants. *Scientia Horticulturae* **210**, 122-129 (2016).

91. Curtis PS, Teeri JA. Seasonal responses of leaf gas-exchange to elevated carbon-dioxide in

Populus grandidentata. *Canadian Journal of Forest Research* **22**, 1320-1325 (1992).

92. Curtis PS*, et al.* Gas exchange, leaf nitrogen, and growth efficiency of *Populus tremuloides* in a CO_2 enriched atmosphere. *Ecological Applications* **10**, 3-17 (2000).

93. Curtis PS, Zak DR, Pregitzer KS, Teeri JA. Above - and belowground response of *Populus grandidentata* to elevated atmospheric CO_2 and soil N availability. *Plant and Soil* **165**, 45-51 (1994).

94. Dahal K, Knowles VL, Plaxton WC, Hüner NPA. Enhancement of photosynthetic performance, water use efficiency and grain yield during long-term growth under elevated CO_2 in wheat and rye is growth temperature and cultivar dependent. *Environmental and Experimental Botany* **106**, 207-220 (2014).

95. Dai L*, et al.* Reduced photosynthetic thermal acclimation capacity under elevated ozone in poplar (*Populus tremula*) saplings. *Global Change Biology* **27**, 2159-2173 (2021).

96. Damesin C, Galera C, Rambal S, Joffre R. Effects of elevated carbon dioxide on leaf gas exchange and growth of cork-oak (*Quercus suber* L.) seedlings. *Annales Des Sciences Forestieres* **53**, 461-467 (1996).

97. Darbah JNT*, et al.* Will photosynthetic capacity of aspen trees acclimate after long-term exposure to elevated CO_2 and O_3? *Environmental Pollution* **158**, 983-991 (2010).

98. Davidson AM, Da Silva D, Saa S, Mann P, DeJong TM. The influence of elevated CO_2 on the photosynthesis, carbohydrate status, and plastochron of young peach (*Prunuspersica*) trees. *Horticulture Environment and Biotechnology* **57**, 364-370 (2016).

99. De Oliveira MF, Marenco RA. Gas exchange, biomass allocation and water-use efficiency in response to elevated CO_2 and drought in andiroba (*Carapa surinamensis*, Meliaceae). *Iforest-Biogeosciences and Forestry* **12**, 61-68 (2019).

100. Del Pozo A, Pérez P, Morcuende R, Alonso A, Martínez-Carrasco R. Acclimatory responses of stomatal conductance and photosynthesis to elevated CO_2 and temperature in wheat crops grown at varying levels of N supply in a Mediterranean environment. *Plant Science* **169**, 908-916 (2005).

101. DesRochers A, van den Driessche R, Thomas BR. NPK fertilization at planting of three hybrid poplar clones in the boreal region of Alberta. *Forest Ecology and Management* **232**, 216-225 (2006).

102. Diksaityte A, Virsile A, Zaltauskaite J, Januskaitiene I, Juozapaitiene G. Growth and photosynthetic responses in *Brassica napus* differ during stress and recovery periods when exposed to combined heat, drought and elevated CO_2. *Plant Physiology and Biochemistry* **142**, 59-72 (2019).

103. Dixon M, Le Thiec D, Garrec JP. Reactions of Norway spruce and beech trees to 2 years of ozone exposure and episodic drought. *Environmental and Experimental Botany* **40**, 77-91 (1998).

104. Dixon M, Thiec DL, Garrec JP. The growth and gas exchange response of soil-planted Norway spruce [*Picea abies* (L.) Karst.] and red oak (*Quercus rubra* L.) exposed to elevated CO_2 and to naturally occurring drought. *New Phytologist* **129**, 265-273 (1995).

105. Djanaguiraman M, Prasad PVV, Boyle DL, Schapaugh WT. High temperature stress and soybean leaves: leaf anatomy and photosynthesis. *Crop Science* **51**, 2125-2131 (2011).

106. Dobson MC, Taylor G, Freersmith PH. The control of ozone uptake by *Picea abies* (L.) karst and *P. sitchensis* (Bong) carr during drought and interacting effects on shoot water relations. *New Phytologist* **116**, 465-474 (1990).

107. Domec J-C, Palmroth S, Ward E, Maier CA, ThÉRÉZien M, Oren RAM. Acclimation of leaf hydraulic conductance and stomatal conductance of *Pinus taeda* (loblolly pine) to long-term growth in elevated CO_2 (free-air CO_2 enrichment) and N-fertilization. *Plant, Cell and Environment* **32**, 1500-1512 (2009).

108. Drake JE*, et al.* The capacity to cope with climate warming declines from temperate to tropical latitudes in two widely distributed *Eucalyptus* species. *Global Change Biology* **21**, 459-472 (2015).

109. Du X*, et al.* Inorganic nitrogen fertilizers induce changes in ammonium assimilation and gas exchange in *Camellia sinensis* L. *Turkish Journal of Agriculture and Forestry* **39**, 28-38 (2015).

110. Du Y, Li J, Wang H, Tang X, Hu F. Effects of high temperature stress on the flag leaf photosynthesis and chlorophyll fluorescence parameters of rice. *Chinese Journal of Ecology* **31**, 2541-2548 (2012).

111. Duan B, Dong T, Zhang X, Zhang Y, Chen J. Ecophysiological responses of two dominant subalpine tree species *Betula albo-sinensis* and *Abies faxoniana* to intra-and interspecific competition under elevated temperature. *Forest Ecology and Management* **323**, 20-27 (2014).

112. Duan B, Zhang X, Li Y, Li L, Korpelainen H, Li C. Plastic responses of *Populus yunnanensis* and *Abies faxoniana* to elevated atmospheric CO_2 and warming. *Forest Ecology and Management* **296**, 33-40 (2013).

113. Duan H, Chaszar B, Lewis JD, Smiths RA, Huxman TE, Tissue DT. CO_2 and temperature effects on morphological and physiological traits affecting risk of drought-induced mortality. *Tree Physiology* **38**, 1138-1151 (2018).

114. Duan H*, et al.* Elevated [CO_2] does not ameliorate the negative effects of elevated temperature on drought-induced mortality in *Eucalyptus radiata* seedlings. *Plant, Cell and Environment* **37**, 1598-1613 (2014).

115. Duan H*, et al.* Drought responses of two gymnosperm species with contrasting stomatal regulation strategies under elevated [CO_2] and temperature. *Tree Physiology* **35**, 756-770 (2015).

116. Duan H, Ontedhu J, Milham P, Lewis JD, Tissue DT. Effects of elevated carbon dioxide and elevated temperature on morphological, physiological and anatomical responses of *Eucalyptus*

tereticornis along a soil phosphorus gradient. *Tree Physiology* **39**, 1821-1837 (2019).

117. Duan H*, et al.* Individual and interactive effects of drought and heat on leaf physiology of seedlings in an economically important crop. *Aob Plants*, (2017).

118. Duan X, Zhang L, Liu W, Guo L, Guo D, Hou X. Effects of nitrogen application on photosynthetic characteristics and yields of oil tree peony *Paeonia ostii* 'Feng Dan'. *Journal of Nanjing Forestry University* **42**, 48-54 (2018).

119. Durand M*, et al.* Impacts of a partial rainfall exclusion in the field on growth and transpiration: consequences for leaf-level and whole-plant water-use efficiency compared to controlled conditions. *Agricultural and Forest Meteorology* **282**, (2020).

120. Dusenge ME, Madhavji S, Way DA. Contrasting acclimation responses to elevated CO_2 and warming between an evergreen and a deciduous boreal conifer. *Global Change Biology* **26**, 3639-3657 (2020).

121. Dwyer SA, Ghannoum O, Nicotra A, Von Caemmerer S. High temperature acclimation of C_4 photosynthesis is linked to changes in photosynthetic biochemistry. *Plant, Cell and Environment* **30**, 53-66 (2007).

122. Eamus D, Berryman CA, Duff GA. Assimilation, stomatal conductance, specific leaf-area and chlorophyll responses to elevated CO_2 of *Maranthes corymbosa*, a tropical monsoon rain forest species. *Australian Journal of Plant Physiology* **20**, 741-755 (1993).

123. Edwards GS, Wullschleger SD, Kelly JM. Growth and physiology of northern red oak preliminary comparisons of mature and seedling responses to ozone. *Environmental Pollution* **83**, 215-221 (1994).

124. Egli P, Maurer S, Gunthardt-Goerg MS, Korner C. Effects of elevated CO_2 and soil quality on leaf gas exchange and above-ground growth in beech-spruce model ecosystems. *New Phytologist* **140**, 185-196 (1998).

125. Eguchi N*, et al.* Changes in petiole hydraulic properties and leaf water flow in birch and oak saplings in a CO_2-enriched atmosphere. *Tree Physiology* **28**, 287-295 (2008).

126. Eller F, Lambertini C, Nguyen LX, Brix H. Increased invasive potential of nonnative *Phragmites australis*: elevated CO_2 and temperature alleviate salinity effects on photosynthesis and growth. *Global Change Biology* **20**, 531-543 (2014).

127. Ellsworth DS. CO_2 enrichment in a maturing pine forest: are CO_2 exchange and water status in the canopy affected? *Plant, Cell and Environment* **22**, 461-472 (1999).

128. Ellsworth DS, Oren R, Huang C, Phillips N, Hendrey GR. Leaf and canopy responses to elevated CO_2 in a pine forest under free-air CO_2 enrichment. *Oecologia* **104**, 139-146 (1995).

129. Elvira S, Alonso R, Inclan R, Bermejo V, Castillo FJ, Gimeno BS. Ozone effects on Aleppo pine seedlings (*Pinus halepensis* Mill) grown in open-top chambers. *Water Air and Soil Pollution* **85**, 1387-1392 (1995).

130. Epron D, Dreyer E. Long term effects of drought on photosynthesis of adult oak trees [*Quercus petraea* Liebl. and *Quercus robur* L.] in a natural stand. *New Phytologist* **125**, 381-389 (1993).

131. Erice G, Irigoyen JJ, Pérez P, Martínez-Carrasco R, Sánchez-Díaz M. Effect of elevated CO_2, temperature and drought on photosynthesis of nodulated alfalfa during a cutting regrowth cycle. *Physiologia Plantarum* **126**, 458-468 (2006).

132. Evans JR, Schortemeyer M, McFarlane N, Atkin OK. Photosynthetic characteristics of 10 *Acacia* species grown under ambient and elevated atmospheric CO_2. *Australian Journal of Plant Physiology* **27**, 13-25 (2000).

133. Fan X*, et al.* Carbon dioxide fertilization effect on plant growth under soil water stress associates with changes in stomatal traits, leaf photosynthesis, and foliar nitrogen of bell pepper (*Capsicum annuum* L.). *Environmental and Experimental Botany* **179**, 104203 (2020).

134. Fang L, Zhao Q, Liu Y, Hao G. The influence of a five-year nitrogen fertilization treatment on hydraulic architecture of *Pinus sylvestris* var. *mongolica* in a water-limited plantation of NE China. *Forest Ecology and Management* **418**, 15-22 (2018).

135. Faralli M, Grove IG, Hare MC, Kettlewell PS, Fiorani F. Rising CO_2 from historical concentrations enhances the physiological performance of *Brassica napus* seedlings under optimal water supply but not under reduced water availability. *Plant, Cell and Environment* **40**, 317-325 (2017).

136. Fares S*, et al.* Impact of high ozone on isoprene emission, photosynthesis and histology of developing *Populus alba* leaves directly or indirectly exposed to the pollutant. *Physiologia Plantarum* **128**, 456-465 (2006).

137. Farfan-Vignolo ER, Asard H. Effect of elevated CO_2 and temperature on the oxidative stress response to drought in *Lolium perenne* L. and *Medicago sativa* L. *Plant Physiology and Biochemistry* **59**, 55-62 (2012).

138. Faria T, Wilkins D, Besford RT, Vaz M, Pereira JS, Chaves MM. Growth at elevated CO_2 leads to down-regulation of photosynthesis and altered response to high temperature in *Quercus suber* L. seedlings. *Journal of Experimental Botany* **47**, 1755-1761 (1996).

139. Fatichi S, Leuzinger S. Reconciling observations with modeling: the fate of water and carbon allocation in a mature deciduous forest exposed to elevated CO_2. *Agricultural and Forest Meteorology* **174**, 144-157 (2013).

140. Fauset S*, et al.* Contrasting responses of stomatal conductance and photosynthetic capacity to warming and elevated CO_2 in the tropical tree species *Alchomea glandulosa* under heatwave conditions. *Environmental and Experimental Botany* **158**, 28-39 (2019).

141. Feng X*, et al.* Nitrogen enhanced photosynthesis of *Miscanthus* by increasing stomatal conductance and phospho*enol*pyruvate carboxylase concentration. *Photosynthetica* **50**, 577-586 (2012).

142. Feng Z, Niu J, Zhang W, Wang X, Yao F, Tian Y. Effects of ozone exposure on sub-tropical evergreen *Cinnamomum camphora* seedlings grown in different nitrogen loads. *Trees* **25**, 617-625 (2011).

143. Feng Z, Wang X, Zheng Q, Feng Z, Xie J, Chen Z. Response of gas exchange of rape to ozone concentration and exposure regimes. *Acta Ecologica Sinica* **26**, 823-829 (2006).

144. Feng Z, Yao F, Chen Z, Wang X, Zheng Q, Feng Z. Response of gas exchange and yield components of field-grown *Triticum aestivum* L. to elevated ozone in China. *Photosynthetica* **45**, 441-446 (2007).

145. Feng Z, Zeng H, Wang X, Zheng Q, Feng Z. Sensitivity of *Metasequoia glyptostroboides* to ozone stress. *Photosynthetica* **46**, 463-465 (2008).

146. Fernandez M*, et al.* Photosynthesis in plants of four tropical species growing under elevated CO_2. *Photosynthetica* **37**, 587-599 (1999).

147. Fontaine V*, et al.* Carbon fixation in *Pinus halepensis* submitted to ozone. opposite response of ribulose-1,5-bisphosphate carboxylase/oxygenase and phosphoenolpyruvate carboxylase. *Physiologia Plantarum* **105**, 187-192 (1999).

148. Fu W*, et al.* Effect of high ozone concentration on photosynthesis of *Betula platyphylla* and *Populus alba x P. berolinensi*. *Chinese Journal of Ecology* **33**, 3184-3190 (2014).

149. Fusaro L*, et al.* Functional indicators of response mechanisms to nitrogen deposition, ozone, and their interaction in two Mediterranean tree species. *Plos One* **12**, (2017).

150. Gao F, Calatayud V, Garcia-Breijo F, Reig-Arminana J, Feng Z. Effects of elevated ozone on physiological, anatomical and ultrastructural characteristics of four common urban tree species in China. *Ecological Indicators* **67**, 367-379 (2016).

151. Gao J*, et al.* Leaf photosynthesis and yield components of mung bean under fully open-air elevated [CO_2]. *Journal of Integrative Agriculture* **14**, 977-983 (2015).

152. Garcia RL*, et al.* Photosynthesis and conductance of spring-wheat leaves: field response to continuous free-air atmospheric CO_2 enrichment. *Plant, Cell and Environment* **21**, 659-669 (1998).

153. Garcia-Forner N*, et al.* Responses of two semiarid conifer tree species to reduced precipitation and warming reveal new perspectives for stomatal regulation. *Plant, Cell and Environment* **39**, 38-49 (2016).

154. Gardner SDL, Freer-Smith PH, Tucker J, Taylor G. Elevated CO_2 protects poplar (*Populus trichocarpa* × *P. deltoides*) from damage induced by O_3: identification of mechanisms. *Functional Plant Biology* **32**, 221 (2005).

155. Gaucher C, Costanzo N, Afif D, Mauffette Y, Chevrier N, Dizengremel P. The impact of elevated ozone and carbon dioxide on young *Acer saccharum* seedlings. *Physiologia Plantarum* **117**, 392-402 (2003).

156. Gaucher C, Dizengremel P, Mauffette Y, Chevrier N. Response of *Acer saccharum* seedlings to elevated O_3 and CO_2 concentrations. *Phytoprotection* **86**, 7-17 (2005).

157. Ge Z*, et al.* Acclimation of photosynthesis in a boreal grass (*Phalaris arundinacea* L.) under different temperature, CO_2, and soil water regimes. *Photosynthetica* **50**, 141-151 (2012).

158. Ge ZM, Zhou X, Kellomaki S, Wang KY, Peltola H, Martikainen PJ. Responses of leaf photosynthesis, pigments and chlorophyll fluorescence within canopy position in a boreal grass (*Phalaris arundinacea* L.) to elevated temperature and CO_2 under varying water regimes. *Photosynthetica* **49**, 172-184 (2011).

159. Ghannoum O*, et al.* Exposure to preindustrial, current and future atmospheric CO_2 and temperature differentially affects growth and photosynthesis in *Eucalyptus*. *Global Change Biology* **16**, 303-319 (2010).

160. Ghannoum O*, et al.* Photosynthetic responses of two eucalypts to industrial-age changes in atmospheric [CO_2] and temperature. *Plant, Cell and Environment* **33**, 1671-1681 (2010).

161. Ghini R*, et al.* Coffee growth, pest and yield responses to free-air CO_2 enrichment. *Climatic Change* **132**, 307-320 (2015).

162. Gimeno TE*, et al.* Conserved stomatal behaviour under elevated CO_2 and varying water availability in a mature woodland. *Functional Ecology* **30**, 700-709 (2016).

163. Goodfellow J, Eamus D, Duff G. Diurnal and seasonal changes in the impact of CO_2 enrichment on assimilation, stomatal conductance and growth in a long-term study of *Mangifera indica* in the wet–dry tropics of Australia. *Tree Physiology* **17**, 291-299 (1997).

164. Grant RF*, et al.* Crop water relations under different CO_2 and irrigation: testing of ecosys with the free air CO_2 enrichment (FACE) experiment. *Agricultural and Forest Meteorology* **95**, 27-51 (1999).

165. Gravano E*, et al.* Ozone symptoms in leaves of woody plants in open-top chambers: ultrastructural and physiological characteristics. *Physiologia Plantarum* **121**, 620-633 (2004).

166. Gray SB*, et al.* Intensifying drought eliminates the expected benefits of elevated carbon dioxide for soybean. *Nature Plants* **2**, 16132 (2016).

167. Greitner CS, Winner WE. Effects of O_3 on alder photosynthesis and symbiosis with *Frankia*. *New Phytologist* **111**, 647-656 (1989).

168. Griffin KL, Tissue DT, Turnbull MH, Whitehead D. The onset of photosynthetic acclimation to elevated CO_2 partial pressure in field-grown *Pinus radiata* D. after 4 years. *Plant, Cell and Environment* **23**, 1089-1098 (2000).

169. Grombone-Guaratini MT, Gaspar M, Oliveira VF, Torres MAMG, do Nascimento A, Aidar MPM. Atmospheric CO_2 enrichment markedly increases photosynthesis and growth in a woody tropical bamboo from the Brazilian Atlantic forest. *New Zealand Journal of Botany* **51**, 275-285 (2013).

170. Grulke NE, Dobrowolski W, Mingus P, Fenn ME. California black oak response to nitrogen amendment at a high O_3, nitrogen-saturated site. *Environmental Pollution* **137**, 536-545 (2005).

171. Gunderson CA, Sholtis JD, Wullschleger SD, Tissue DT, Hanson PJ, Norby RJ. Environmental and stomatal control of photosynthetic enhancement in the canopy of a sweetgum (*Liquidambar styraciflua* L.) plantation during 3 years of CO_2 enrichment. *Plant, Cell and Environment* **25**, 379-393 (2002).

172. Guo Q, Wu Y, Wang W. Physiological and biochemical response differences of different wheat cultivars to drought, heat and co-stress. *Shandong Agricultural Sciences* **46**, 32-38 (2014).

173. Guo X*, et al.* Effects of nitrogen addition on growth and photosynthetic characteristics of *Acer truncatum* seedlings. *Dendrobiology* **72**, 151-161 (2014).

174. Guo Y*, et al.* Photosynthetic characteristics of *Stipa purpurea* under irrigation in northern Tibet and its short-term response to temperature and CO_2 concentration. *Chinese Journal of Plant Ecology* **35**, 311-321 (2011).

175. Habermann E*, et al.* Stomatal development and conductance of a tropical forage legume are regulated by elevated [CO_2] under moderate warming. *Frontiers in Plant Science* **10**, (2019).

176. Hakala K, Helio R, Tuhkanen E, Kaukoranta T. Photosynthesis and Rubisco kinetics in spying wheat and meadow fescue under conditions of simulated climate change with elevated CO_2 and increased temperatures. *Agricultural and Food Science in Finland* **8**, 441-457 (1999).

177. Hamerlynck EP, Huxman TE, Charlet TN, Smith SD. Effects of elevated CO_2 (FACE) on the functional ecology of the drought-deciduous Mojave desert shrub, *Lycium andersonii*. *Environmental and Experimental Botany* **48**, 93-106 (2002).

178. Hamerlynck EP, Huxman TE, Loik ME, Smith SD. Effects of extreme high temperature, drought and elevated CO_2 on photosynthesis of the Mojave Desert evergreen shrub, *Larrea tridentata*. *Plant Ecology* **148**, 183-193 (2000).

179. Hamerlynck EP*, et al.* Photosynthetic responses of *Larrea tridentata* to a step-increase in atmospheric CO_2 at the Nevada desert FACE facility. *Journal of Arid Environments* **44**, 425-436 (2000).

180. Han B*, et al.* Effects of N addition on photosynthetic characteristics and leaf senescence in *Stipa bungeana* of steppe grasslands in the Loess Plateau. *Pratacultural Science* **33**, 1070-1076 (2016).

181. Han C, Liu Q, Yang Y. Short term effects of experimental warming and enhanced ultraviolet-B radiation on photosynthesis and antioxidant defense of *Picea asperata* seedlings. *Plant Growth Regulation* **58**, 153-162 (2009).

182. Han S, Zhou Y, Wang C, Zhang J, Zou C. Ecophysiological responses and carbon distribution of *Pinus koraiensis* seedlings to elevated carbon dioxide. *Journal of Forestry Research* **11**, 149-149 (2000).

183. Hanson PJ, Samuelson LJ, Wullschleger SD, Tabberer TA, Edwards GS. Seasonal patterns of light-saturated photosynthesis and leaf conductance for mature and seedling *Quercus rubra* L. foliage: differential sensitivity to ozone exposure. *Tree Physiology* **14**, 1351-1366 (1994).

184. Hao X, Han X, Li P, Yang H, Lin E. Effects of elevated atmospheric CO_2 concentration on mung bean leaf photosynthesis and chlorophyll fluorescence parameters. *Chinese Journal of Applied Ecology* **22**, 2776-2780 (2011).

185. Hao X*, et al.* Effects of air CO_2 enrichment on growth and photosynthetic physiology of millet. *Journal of Nuclear Agricultural Sciences* **24**, 589-593 (2010).

186. Hao Y*, et al.* Effects of ozone stress and drought stress on photosynthesis characteristics of *Syzygium hainanense* and *Alstonia scholaris* seedlings. *Journal of Anhui Agricultural University* **41**, 193-197 (2014).

187. Hao Z, Zhou C, Liu D, Wei M, Tao J. Effects of high temperature stress on photosynthesi, chlorophyll fluorescence and ultrastructure of herbaceous peony (*Paeonia lactiflora* Pall.). *Molecular Plant Breeding* **15**, 2359-2367 (2017).

188. Hartikainen K*, et al.* Impact of elevated temperature and ozone on the emission of volatile organic compounds and gas exchange of silver birch (*Betula pendula* Roth.). *Environmental and Experimental Botany* **84**, 33-43 (2012).

189. Hättenschwiler S, Handa IT, Egli L, Asshoff R, Ammann W, Körner C. Atmospheric CO_2 enrichment of alpine treeline conifers. *New Phytologist* **156**, 363-375 (2002).

190. Havranek WM, Wieser G. Design and testing of twig chambers for ozone fumigation and gas exchange measurements in mature trees. *Proceedings of the Royal Society of Edinburgh* **102**, 541-546 (1994).

191. He J, Wolfe-Bellin KS, Bazzaz FA. Leaf level physiology, biomass, and reproduction of *Phytolacca americana* under conditions of elevated CO_2 and altered temperature regimes. *International Journal of Plant Sciences* **166**, 615-622 (2005).

192. He X, Fu S, Chen W, Zhao T, Xu S, Tuba Z. Changes in effects of ozone exposure on growth, photosynthesis, and respiration of *Ginkgo biloba* in Shenyang urban area. *Photosynthetica* **45**, 555-561 (2007).

193. He Z, Cai Z, Cai C. Effect of water and nitrogen on photosynthetic characteristics and growth of *Rauvolfia vomitoria*. *Chinese Journal of Eco-Agriculture* **18**, 758-764 (2010).

194. Heath J. Stomata of trees growing in CO_2 enriched air show reduced sensitivity to vapour pressure deficit and drought. *Plant, Cell and Environment* **21**, 1077-1088 (1998).

195. Heath J, Kerstiens G. Effects of elevated CO_2 on leaf gas exchange in beech and oak at two levels of nutrient supply: consequences for sensitivity to drought in beech. *Plant, Cell and Environment* **20**, 57-67 (1997).

196. Hebbar KB, Apshara E, Chandran KP, Prasad PVV. Effect of elevated CO_2, high

temperature, and water deficit on growth, photosynthesis, and whole plant water use efficiency of cocoa (*Theobroma cacao* L.). *International Journal of Biometeorology* **64**, 47-57 (2020).

197. Herbinger K*, et al.* Tree age dependence and within-canopy variation of leaf gas exchange and antioxidative defence in *Fagus sylvatica* under experimental free-air ozone exposure. *Environmental Pollution* **137**, 476-482 (2005).

198. Herrick JD, Maherali H, Thomas RB. Reduced stomatal conductance in sweetgum (*Liquidambar styraciflua*) sustained over long term CO_2 enrichment. *New Phytologist* **162**, 387-396 (2004).

199. Hileman DR*, et al.* Canopy photosynthesis and transpiration of field grown cotton exposed to free-air CO_2 enrichment (FACE) and differential irrigation. *Agricultural and Forest Meteorology* **70**, 189-207 (1994).

200. Hirano A, Hongo I, Koike T. Morphological and physiological responses of Siebold's beech (*Fagus crenata*) seedlings grown under CO_2 concentrations ranging from pre-industrial to expected future levels. *Landscape and Ecological Engineering* **8**, 59-67 (2012).

201. Hiraoka Y*, et al.* Species characteristics and intraspecific variation in growth and photosynthesis of *Cryptomeria japonica* under elevated O_3 and CO_2. *Tree Physiology* **37**, 733-743 (2017).

202. Hlaváčová M*, et al.* Interactive effects of high temperature and drought stress during stem elongation, anthesis and early grain filling on the yield formation and photosynthesis of winter wheat. *Field Crops Research* **221**, 182-195 (2018).

203. Hoshika Y, Carriero G, Feng ZZ, Zhang YL, Paoletti E. Determinants of stomatal sluggishness in ozone-exposed deciduous tree species. *Science of The Total Environment* **481**, 453-458 (2014).

204. Hoshika Y, Watanabe M, Inada N, Koike T. Growth and leaf gas exchange in three birch species exposed to elevated ozone and CO_2 in summer. *Water Air and Soil Pollution* **223**, 5017-5025 (2012).

205. Hou W, Sun A, Chen H, Yang F, Pan J, Guan M. Effects of chilling and high temperatures on photosynthesis and chlorophyll fluorescence in leaves of watermelon seedlings. *Biologia Plantarum* **60**, 148-154 (2016).

206. Huang C, Zhu X, Zhang L, Sun X, Hua W. Effects of drought and high temperature on photosynthesis and chlorophyll fluorescence characteristics of rapeseed leaves. *Chinese Journal of Oil Crop Sciences* **39**, 342-350 (2017).

207. Huang G, Zhang Q, Wei X, Peng S, Li Y. Nitrogen can alleviate the inhibition of photosynthesis caused by high temperature stress under both steady-state and flecked irradiance. *Frontiers in plant science* **8**, 945-953 (2017).

208. Huang L*, et al.* Effects of high temperature on leaf gas exchange and chlorophyll

fluorescence parameters of the north highbush blueberry. *Chinese Journal of Ecology* **35**, 871-879 (2016).

209. Hui J, Ye Q. Effects of elevated CO_2 concentration on photosynthsis, growth and development of the bromeliad *Guzmania*. *Chinese Journal of Ecology* **32**, 424-430 (2008).

210. Hussain HA*, et al.* Interactive effects of drought and heat stresses on morpho-physiological attributes, yield, nutrient uptake and oxidative status in maize hybrids. *Scientific Reports* **9**, (2019).

211. Huxman TE, Hamerlynck EP, Loik ME, Smith SD. Gas exchange and chlorophyll fluorescence responses of three south-western *Yucca* species to elevated CO_2 and high temperature. *Plant, Cell and Environment* **21**, 1275-1283 (1998).

212. Huxman TE, Smith SD. Photosynthesis in an invasive grass and native forb at elevated CO_2 during an El Niño year in the Mojave desert. *Oecologia* **128**, 193-201 (2001).

213. Inclán R, Gimeno BS, Dizengremel P, Sanchez M. Compensation processes of Aleppo pine (*Pinus halepensis* Mill.) to ozone exposure and drought stress. *Environmental Pollution* **137**, 517-524 (2005).

214. Inoue Y, Ichie T, Kenzo T, Yoneyama A, Kumagai T, Nakashizuka T. Effects of rainfall exclusion on leaf gas exchange traits and osmotic adjustment in mature canopy trees of *Dryobalanops aromatica* (Dipterocarpaceae) in a Malaysian tropical rain forest. *Tree Physiology* **37**, 1-11 (2017).

215. Jayawardena DM, Heckathorn SA, Bista DR, Boldt JK. Elevated carbon dioxide plus chronic warming causes dramatic increases in leaf angle in tomato, which correlates with reduced plant growth. *Plant, Cell and Environment* **42**, 1247-1256 (2019).

216. Jiang L, Guo R, Zhu T, Niu X, Guo J, Sun W. Water- and plant-mediated responses of ecosystem carbon fluxes to warming and nitrogen addition on the songnen grassland in northeast China. *Plos One* **7**, e45205 (2012).

217. Jiang S*, et al.* Interactive effects of elevated carbon dioxide and water on the growth and development of winter wheat. *Chinese Journal of Agrometeorology* **34**, 403-409 (2013).

218. Jiang Y, Zhang Q, zhang S, Wang G, Yue W, Yao Y. Responses of photosynthetic characteristics, stomatal conductance and transpiration of wheat to the increase of atmospheric CO_2 concentration. *Journal of Anhui Agriculture University* **32**, 169-173 (2005).

219. Jiang Y, Zhang S, Zhang Q. Effects of elevated atmospheric CO_2 concentration on photo-physiological characteristics of tea plant. *Journal of Tea Science* **25**, 43-48 (2005).

220. Jin J, Lauricella D, Armstrong R, Sale P, Tang C. Phosphorus application and elevated CO_2 enhance drought tolerance in field pea grown in a phosphorus-deficient vertisol. *Annals of Botany* **116**, 975-985 (2015).

221. Jin S, Li X, Wang J. Effects of high temperature stress on photosynthesis in *Ficus concinna*

var.subsessilis. *Chinese Agricultural Science Bulletin* **25**, 83-87 (2009).

222. Jing L*, et al.* Effect of increasing atmospheric CO_2 concentration on photosynthetic diurnal variation characteristics of hybrid rice: a FACE study. *Acta Ecologica Sinica* **37**, 2033-2044 (2017).

223. Jo H, Chang H, An J, Cho MS, Son Y. Species specific physiological responses of *Pinus densiflora* and *Larix kaempferi* seedlings to open-field experimental warming and precipitation manipulation. *Forest Science and Technology* **15**, 44-50 (2019).

224. Johnsen KH. Growth and ecophysiological responses of black spruce seedlings to elevated CO_2 under varied water and nutrient additions. *Canadian Journal of Forest Research* **23**, 1033-1042 (1993).

225. Kalva MS, Sreeharsha RV, Mudalkar S, Reddy AR. Persistent stimulation of photosynthesis in short rotation coppice mulberry under elevated CO_2 atmosphere. *Journal of Photochemistry and Photobiology B: Biology* **137**, 21-30 (2014).

226. Keel SG, Pepin S, Leuzinger S, Koerner C. Stomatal conductance in mature deciduous forest trees exposed to elevated CO_2. *Trees* **21**, 151-159 (2007).

227. Kellomäki S, Wang K. Photosynthetic responses of Scots pine to elevated CO_2 and nitrogen supply: results of a branch-in-bag experiment. *Tree Physiology* **17**, 231-240 (1997).

228. Kellomaki S, Wang KY. Effects of elevated O_3 and CO_2 concentrations on photosynthesis and stomatal conductance in Scots pine. *Plant, Cell and Environment* **20**, 995-1006 (1997).

229. Kets K, Darbah JNT, Sober A, Riikonen J, Sober J, Karnosky DF. Diurnal changes in photosynthetic parameters of *Populus tremuloides*, modulated by elevated concentrations of CO_2 and/or O_3 and daily climatic variation. *Environmental Pollution* **158**, 1000-1007 (2010).

230. Keutgen N, Chen K. Responses of citrus leaf photosynthesis, chlorophyll fluorescence, macronutrient and carbohydrate contents to elevated CO_2. *J Plant Physiol* **158**, 1307-1316 (2001).

231. Killi D*, et al.* Photosynthetic and morphological responses of oak species to temperature and CO_2 increased to levels predicted for 2050. *Urban Forestry and Urban Greening* **31**, 26-37 (2018).

232. Killi D, Bussotti F, Raschi A, Haworth M. Adaptation to high temperature mitigates the impact of water deficit during combined heat and drought stress in C_3 sunflower and C_4 maize varieties with contrasting drought tolerance. *Physiologia Plantarum* **159**, 130-147 (2017).

233. Kim S-H, Gitz DC, Sicher RC, Baker JT, Timlin DJ, Reddy VR. Temperature dependence of growth, development, and photosynthesis in maize under elevated CO_2. *Environmental and Experimental Botany* **61**, 224-236 (2007).

234. Kitao M, Komatsu M, Yazaki K, Kitaoka S, Tobita H. Growth overcompensation against O_3 exposure in two Japanese oak species, *Quercus mongolica* var. crispula and *Quercus serrata*,

grown under elevated CO_2. *Environmental Pollution* **206**, 133-141 (2015).

235. Kivimaenpaa M, Riikonen J, Ahonen V, Tervahauta A, Holopainen T. Sensitivity of Norway spruce physiology and terpenoid emission dynamics to elevated ozone and elevated temperature under open-field exposure. *Environmental and Experimental Botany* **90**, 32-42 (2013).

236. Kivimaenpaa M*, et al.* Warming and elevated ozone differently modify needle anatomy of Norway spruce (*Picea abies*) and Scots pine (*Pinus sylvestris*). *Canadian Journal of Forest Research* **47**, 488-499 (2017).

237. Klein T*, et al.* Growth and carbon relations of mature *Picea abies* trees under 5 years of free-air CO_2 enrichment. *Journal of Ecology* **104**, 1720-1733 (2016).

238. Kobayakawa H, Imai K. Effect of acute O_3 exposure on leaf photosynthesis of paddy rice grown at elevated CO_2 concentration. *Environmental Control in Biology* **50**, 31-39 (2012).

239. Kosvancova M*, et al.* Photosynthetic induction in broadleaved *Fagus sylvatica* and coniferous *Picea abies* cultivated under ambient and elevated CO_2 concentrations. *Plant Science* **177**, 123-130 (2009).

240. Kroner Y, Way DA. Carbon fluxes acclimate more strongly to elevated growth temperatures than to elevated CO_2 concentrations in a northern conifer. *Global Change Biology* **22**, 2913-2928 (2016).

241. Kronfu G, Polle A, Tausz M, Havranek WM, Wieser G. Effects of ozone and mild drought stress on gas exchange, antioxidants and chloroplast pigments in current-year needles of young Norway spruce (*Picea abies* (L.) karst.). *Trees* **12**, 482-489 (1998).

242. Kubiske ME, Pregitzer KS. Ecophysiological responses to simulated canopy gaps of two tree species of contrasting shade tolerance in elevated CO_2. *Functional Ecology* **11**, 24-32 (1997).

243. Kull O*, et al.* Photosynthetic responses of aspen clones to simultaneous exposures of ozone and CO_2. *Canadian Journal of Forest Research* **26**, 639-648 (1996).

244. Lahive F, Hadley P, Daymond AJ. The impact of elevated CO_2 and water deficit stress on growth and photosynthesis of juvenile cacao (*Theobroma cacao* L.). *Photosynthetica* **56**, 911-920 (2018).

245. Lambreva M, Stoyanova-Koleva D, Baldjiev G, Tsonev T. Early acclimation changes in the photosynthetic apparatus of bean plants during short-term exposure to elevated CO_2 concentration under high temperature and light intensity. *Agriculture, Ecosystems and Environment* **106**, 219-232 (2005).

246. Lavoir AV*, et al.* Drought reduced monoterpene emissions from the evergreen Mediterranean oak *Quercus ilex*: results from a throughfall displacement experiment. *Biogeosciences* **6**, 1167-1180 (2009).

247. Lawson JR, Fryirs KA, Leishman MR. Interactive effects of waterlogging and atmospheric

CO_2 concentration on gas exchange, growth and functional traits of Australian riparian tree seedlings. *Ecohydrology* **10**, (2017).

248. Leakey ADB, Bernacchi CJ, Dohleman FG, Ort DR, Long SP. Will photosynthesis of maize (*Zea mays*) in the US Corn Belt increase in future CO_2 rich atmospheres? An analysis of diurnal courses of CO_2 uptake under free-air concentration enrichment (FACE). *Global Change Biology* **10**, 951-962 (2004).

249. Leakey ADB, Press MC, Scholes JD, Watling JR. Relative enhancement of photosynthesis and growth at elevated CO_2 is greater under sunflecks than uniform irradiance in a tropical rain forest tree seedling. *Plant, Cell and Environment* **25**, 1701-1714 (2002).

250. Leakey ADB*, et al.* Photosynthesis, productivity, and yield of maize are not affected by open-air elevation of CO_2 concentration in the absence of drought. *Plant physiology* **140**, 779-790 (2006).

251. Leaky ADB, Press MC, Scholes JD. High-temperature inhibition of photosynthesis is greater under sunflecks than uniform irradiance in a tropical rainforest tree seedling. *Plant, Cell and Environment* **26**, 1681-1690 (2003).

252. Lee JK*, et al.* Effects of elevated temperature and ozone in *Brassica juncea* L.: growth, physiology, and ROS accumulation. *Forests* **11**, (2020).

253. Lee TD, Tjoelker MG, Ellsworth DS, Reich PB. Leaf gas exchange responses of 13 prairie grassland species to elevated CO_2 and increased nitrogen supply. *New Phytologist* **150**, 405-418 (2001).

254. Lenka NK*, et al.* Carbon dioxide and temperature elevation effects on crop evapotranspiration and water use efficiency in soybean as affected by different nitrogen levels. *Agricultural Water Management* **230**, 105936 (2020).

255. Leon Sánchez L, Nicolás E, Nortes PA, Maestre FT, Querejeta JI. Photosynthesis and growth reduction with warming are driven by nonstomatal limitations in a Mediterranean semi-arid shrub. *Ecology and Evolution* **6**, 2725-2738 (2016).

256. Leonardi S, Langebartels C. Fall exposure of beech saplings (*Fagus sylvatica* L.) to ozone and simulated acidic mist - effects on gas-exchange and leachability. *Water Air and Soil Pollution* **54**, 143-153 (1990).

257. Leverenz JW, Bruhn D, Saxe H. Responses of two provenances of *Fagus sylvatica* seedlings to a combination of four temperature and two CO_2 treatments during their first growing season: gas exchange of leaves and roots. *New Phytologist* **144**, 437-454 (1999).

258. Lewis JD*, et al.* Rising temperature may negate the stimulatory effect of rising CO_2 on growth and physiology of Wollemi pine (*Wollemia nobilis*). *Functional Plant Biology* **42**, 836-850 (2015).

259. Lewis JD, Smith RA, Ghannoum O, Logan BA, Phillips NG, Tissue DT. Industrial-age

changes in atmospheric CO_2 and temperature differentially alter responses of faster- and slower-growing *Eucalyptus* seedlings to short-term drought. *Tree Physiology* **33**, 475-488 (2013).

260. Li D*, et al.* Effects of elevated CO_2 on the growth, seed yield, and water use efficiency of soybean (*Glycine max* (L.) Merr.) under drought stress. *Agricultural Water Management* **129**, 105-112 (2013).

261. Li D, Mo J, Fang Y, Cai Xa, Xue J, Xu G. Effects of simulated nitrogen deposition on growth and photosynthesis of *Schima superba*, *Castanopsis chinensis* and *Cryptocarya concinna* seedlings. *Acta Ecologica Sinica* **24**, 876-882 (2004).

262. Li J, Chang Y, Yu J. Changes of some photosynthetic properties and photosystem II photochemical activities in cucumber seedings under high temperature stress. *Plant Physiology Journal* **43**, 1085-1088 (2007).

263. Li J, Erickson JE, Peresta G, Drake BG. Evapotranspiration and water use efficiency in a Chesapeake Bay wetland under carbon dioxide enrichment. *Global Change Biology* **16**, 234-245 (2010).

264. Li J, Johnson DP, Dijkstra P, Hungate BA, Hinkle CR, Drake BG. Elevated CO_2 mitigates the adverse effects of drought on daytime net ecosystem CO_2 exchange and photosynthesis in a Florida scrub-oak ecosystem. *Photosynthetica* **45**, 51-58 (2007).

265. Li JH*, et al.* Direct and indirect effects of elevated CO_2 on transpiration from *Quercus myrtifolia* in a scrub-oak ecosystem. *Global Change Biology* **9**, 96-105 (2003).

266. Li L, Duan B, Deng D, Tu W, Zhang Y. Soil salinity alters the sexual responses to elevated CO_2 and temperature in growth and leaf traits of a dioecious plant. *Canadian Journal of Forest Research* **44**, 1292-1301 (2014).

267. Li L, Manning WJ, Tong L, Wang X. Chronic drought stress reduced but not protected Shantung maple (*Acer truncatum* Bunge) from adverse effects of ozone (O_3) on growth and physiology in the suburb of Beijing, China. *Environmental Pollution* **201**, 34-41 (2015).

268. Li M. Effects of ozone on photosynthesis of several plants. *Environmental Science* **36**, 1888-1901 (2015).

269. Li M, Chao J, Gu W, Hou H. Effects of high-temperature stress on photosynthetic characteristics and physiological indexes of *Atractylodes lancea* (Thunb.) from different producing areas. *Journal of Southern Agriculture* **46**, 1651-1657 (2015).

270. Li M, Guo W, Du N, Xu Z, Guo X. Nitrogen deposition does not affect the impact of shade on *Quercus acutissima* seedlings. *Plos One* **13**, e0194261 (2018).

271. Li P*, et al.* Photosynthesis and yield response to elevated CO_2, C_4 plant foxtail millet behaves similarly to C_3 species. *Plant Science* **285**, 239-247 (2019).

272. Li Q, Liu B, Zou Z. Effects of doubled CO_2 concentration on photosynthetic characteristics of cucumber seedlings under drought stresses. *Scientia Agricultura Sinica* **44**, 963-971 (2011).

273. Li Q*, et al.* Effects of ozone and drought stress on photosynthetic physiology of three species seedlings in south china. *Forestry and Environmental Science* **30**, 45-52 (2014).

274. Li T, Li M, Li Y, Lu S. Effect of $CaCl_2$ and salicylic on the photosynthesis in the leaves of tomato under daytime sub-high temperature. *Acta Agriculturae Boreali-occidentalis Sinica* **18**, 284-289 (2009).

275. Li X, Dong J, Gruda NS, Chu W, Duan Z. Interactive effects of the CO_2 enrichment and nitrogen supply on the biomass accumulation, gas exchange properties, and mineral elements concentrations in cucumber plants at different growth stages. *Agronomy* **10**, (2020).

276. Li X, Kristiansen K, Rosenqvist E, Liu F. Elevated CO_2 modulates the effects of drought and heat stress on plant water relations and grain yield in wheat. *Journal of Agronomy and Crop Science* **205**, 362-371 (2019).

277. Li Y, Chen J, Ai S, Shi H. Responses of leaf water potential and gas exchange to the precipitation manipulation in two shrubs on the Chinese Loess Plateau. *Journal of Arid Land* **12**, 267-282 (2020).

278. Li Y, Chen S, Li Y, Zhuang M, Guo Z, Gu D. Photosynthetic physio-response of *Oligostachyum lubricum* to atmospheric ozone stress. *Ecologic Science* **31**, 390-395 (2012).

279. Li Y, Dong T, Duan B, Zhang Y. Physiological responses of *Abies faxoniana* populations from different elevations to increased CO_2 and N application. *Acta Physiologiae Plantarum* **37**, 1724-1735 (2014).

280. Li Y, Li X, Yu J, Liu F. Effect of the transgenerational exposure to elevated CO_2 on the drought response of winter wheat: stomatal control and water use efficiency. *Environmental and Experimental Botany* **136**, 78-84 (2017).

281. Li Y, Li Y, Chen S, Zhuang M, Guo Z, Yang Q. Impacts of elevated atmospheric ozone concentration on the photosynthetic physiology of *Phyllostachys edulis*. *Chinese Journal of Ecology* **32**, 344-350 (2013).

282. Li Y, Zhang X, Yang Y, Duan B. Soil cadmium toxicity and nitrogen deposition differently affect growth and physiology in *Toxicodendron vernicifluum* seedlings. *Acta Physiologiae Plantarum* **35**, 529-540 (2013).

283. Li Y, Zhang Y, Zhang X, Korpelainen H, Berninger F, Li C. Effects of elevated CO_2 and temperature on photosynthesis and leaf traits of an understory dwarf bamboo in subalpine forest zone, China. *Physiologia Plantarum* **148**, 261-272 (2013).

284. Li Z*, et al.* The influence of precipitation regimes and elevated CO_2 on photosynthesis and biomass accumulation and partitioning in seedlings of the rhizomatous perennial grass *Leymus chinensis*. *Plos One* **9**, e103633 (2014).

285. Liang J, Yang J, Ye Y. Effects of temperatures on photosynthetic characteristics and saponins of *Paris polyphylla*. *Guihaia* **36**, 323-328 (2016).

286. Liang N, Maruyama K. Interactive effects of CO_2 enrichment and drought stress on gas exchange and water-use efficiency in *Alnus firma*. *Environmental and Experimental Botany* **35**, 353-361 (1995).

287. Liang N, Maruyama K, Huang Y. Interactions of elevated CO_2 and drought stress in gas-exchange and water-use efficiency in 3 temperate deciduous tree species. *Photosynthetica* **31**, 529-539 (1995).

288. Liao Y, Fan H, Li Y, Liu W, Yuan Y. Effects of simulated nitrogen deposition on growth and photosynthesis of 1-year-old Chinese fir (*Cunninghamia lanceolata*) seedlings. *Acta Ecologica Sinica* **30**, 150-154 (2010).

289. Lima WdP, Jarvis P, Rhizopoulou S. Stomatal responses of *Eucalyptus* species to elevated CO_2 concentration and drought stress. *Scientia Agricola* **60**, 231-238 (2003).

290. Limousin J-M*, et al.* Regulation and acclimation of leaf gas exchange in a piñon-juniper woodland exposed to three different precipitation regimes. *Plant, Cell and Environment* **36**, 1812-1825 (2013).

291. Liu A, Chen S, Wang M, Wang F, He C. Effects of heat stress on photosynthesis and chlorophyll fluorescence parameters in tomato seedlings. *Acta Agriculturae Boreali-occidentalis Sinica* **19**, 145-148 (2010).

292. Liu H, Wang Y, Shen Y. Effects of doubled atmospheric CO_2 concentration on photosynthesis of *Dendrobium chrysotoxum*. *Guangdong Agricultural Sciences* **39**, 6-9 (2012).

293. Liu J, Li J, Zhang J. Influences of drought stress on photosynthetic characteristics and water use efficiency of 4 tree species under elevated CO_2 concentration. *Forest Research* **28**, 339-345 (2015).

294. Liu N, Dang Q, Parker WH. Genetic variation of *Populus tremuloides* in ecophysiological responses to CO_2 elevation. *Canadian Journal of Botany* **84**, 294-302 (2006).

295. Liu S, Barton C, Lee H, Jarvis PG, Durrant D. Long term response of Sitka spruce (*Picea sitchensis*) to CO_2 enrichment and nitrogen supply. I. Growth, biomass allocation and physiology. *Plant Biosystems* **136**, 189-198 (2002).

296. Liu S, Teskey RO. Responses of foliar gas exchange to long-term elevated CO_2 concentrations in mature loblolly pine trees. *Tree Physiology* **15**, 351-359 (1995).

297. Liu X*, et al.* Effects of soil water and nitrogen availability on photosynthesis and water use efficiency of Robinia pseudoacacia seedlings. *Journal of Environmental Sciences* **25**, 585-595 (2013).

298. Liu X, Yang X, Wang X, Zhang S. Effects of simulated nitrogen deposition on growth and photosynthetic characteristics of *Quercus wutaishanica* and *Acer pictum* subsp. *mono* in a warm-temperate deciduous broadleaved forest. *Chinese Journal of Plant Ecology* **43**, 197-207 (2019).

299. Liu X*, et al.* Increased CO_2 concentrations increasing water use efficiency and improvement PSII function of mulberry seedling leaves under drought stress. *Journal of Plant Interactions* **14**, 213-223 (2019).

300. Liu Y, Li Z, Han J, Mu C. Influences of precipitation regimes and elevated CO_2 on photosynthesis and biomass accumulation in *Leymus chinensis*. *Acta Prataculturae Sinica* **24**, 128-136 (2015).

301. Llusia J, Bermejo-Bermejo V, Calvete-Sogo H, Penuelas J. Decreased rates of terpene emissions in *Ornithopus compressus* L. and *Trifolium striatum* L. by ozone exposure and nitrogen fertilization. *Environmental Pollution* **194**, 69-77 (2014).

302. Loats KV, Rebbeck J. Interactive effects of ozone and elevated carbon dioxide on the growth and physiology of black cherry, green ash, and yellow-poplar seedlings. *Environmental Pollution* **106**, 237-248 (1999).

303. Locke AM, Sack L, Bernacchi CJ, Ort DR. Soybean leaf hydraulic conductance does not acclimate to growth at elevated CO_2 or temperature in growth chambers or in the field. *Annals of Botany* **112**, 911-918 (2013).

304. Lodge RJ, Dijkstra P, Drake BG, Morison JIL. Stomatal acclimation to increased CO_2 concentration in a Florida scrub oak species *Quercus myrtifolia* Willd. *Plant, Cell and Environment* **24**, 77-88 (2001).

305. Loik ME. Sensitivity of water relations and photosynthesis to summer precipitation pulses for *Artemisia tridentata* and *Purshia tridentata*. *Plant Ecology* **191**, 95-108 (2007).

306. Lotfiomran N, Koehl M, Fromm J. Interaction effect between elevated CO_2 and fertilization on biomass, gas exchange and C/N ratio of european beech (*Fagus sylvatica* L.). *Plants* **5**, (2016).

307. Lu T*, et al.* Sub-high temperature and high light intensity induced irreversible inhibition on photosynthesis system of tomato plant (*Solanum lycopersicum* L.). *Frontiers in Plant Science* **8**, 365-380 (2017).

308. Lu X*, et al.* Plant acclimation to long-term high nitrogen deposition in an N-rich tropical forest. *Proceedings of the National Academy of Sciences* **115**, 5187-5192 (2018).

309. Ludwig F, Jewitt RA, Donovan LA. Nutrient and water addition effects on day and night time conductance and transpiration in a C_3 desert annual. *Oecologia* **148**, 219-225 (2006).

310. Luo H, Ma L, Duan W, Li S, Wang L. Influence of heat stress on photosynthesis in *Vitis vinifera* L. Cv. cabernet sauvignon. *Scientia Agricultura Sinica* **43**, 2744-2750 (2010).

311. Luomala EM, Laitinen K, KellomÄki S, Vapaavuori E. Variable photosynthetic acclimation in consecutive cohorts of Scots pine needles during 3 years of growth at elevated CO_2 and elevated temperature. *Plant, Cell and Environment* **26**, 645-660 (2003).

312. Ma F, Xu T, Zhang X, Zhao C. Ecophysiological responses of seedlings of hippophae

neurocarpa to elevated carbon dioxide. *Scientia Silvae Sinicae* **48**, 30-35 (2012).

313. Madhana Sekhar K, Rachapudi VS, Mudalkar S, Reddy AR. Persistent stimulation of photosynthesis in short rotation coppice mulberry under elevated CO_2 atmosphere. *Journal of Photochemistry and Photobiology B: Biology* **137**, 21-30 (2014).

314. Maenpaa M, Riikonen J, Kontunen-Soppela S, Rousi M, Oksanen E. Vertical profiles reveal impact of ozone and temperature on carbon assimilation of *Betula pendula* and *Populus tremula*. *Tree physiology* **31**, 808-818 (2011).

315. Maggard AO, Will RE, Wilson DS, Meek CR, Vogel JG. Fertilization reduced stomatal conductance but not photosynthesis of *Pinus taeda* which compensated for lower water availability in regards to growth. *Forest Ecology and Management* **381**, 37-47 (2016).

316. Maherali H, DeLucia EH. Interactive effects of elevated CO_2 and temperature on water transport in ponderosa pine. *American Journal of Botany* **87**, 243-249 (2000).

317. Mao Q, Watanabe M, Imori M, Kim YS, Kita K, Koike T. Photosynthesis and nitrogen allocation in needles in the sun and shade crowns of hybrid larch saplings: effect of nitrogen application. *Photosynthetica* **50**, 422-428 (2012).

318. Mao Q, Watanabe M, Makoto K, Kita K, Koike T. High nitrogen deposition may enhance growth of a new hybrid larch F_1 growing at two phosphorus levels. *Landscape and Ecological Engineering* **10**, 1-8 (2014).

319. Marchi S*, et al.* Physiological and morphological responses of grassland species to elevated atmospheric CO_2 concentrations in FACE-systems and natural CO_2 springs. *Functional Plant Biology* **31**, 181-194 (2004).

320. Markelz RJC, Strellner RS, Leakey ADB. Impairment of C_4 photosynthesis by drought is exacerbated by limiting nitrogen and ameliorated by elevated [CO_2] in maize. *Journal of Experimental Botany* **62**, 3235-3246 (2011).

321. Martinez-Carrasco R, Perez P, Morcuende R. Interactive effects of elevated CO_2, temperature and nitrogen on photosynthesis of wheat grown under temperature gradient tunnels. *Environmental and Experimental Botany* **54**, 49-59 (2005).

322. Martinez-Sancho E, Vasconez Navas LK, Seidel H, Dorado-Linan I, Menzel A. Responses of contrasting tree functional types to air warming and drought. *Forests* **8**, 450-468 (2017).

323. Martin-Stpaul NK*, et al.* The temporal response to drought in a Mediterranean evergreen tree: comparing a regional precipitation gradient and a throughfall exclusion experiment. *Global Change Biology* **19**, 2413-2426 (2013).

324. Marzuoli R, Monga R, Finco A, Chiesa M, Gerosa G. Increased nitrogen wet deposition triggers negative effects of ozone on the biomass production of *Carpinus betulus* L. young trees. *Environmental and Experimental Botany* **152**, 128-136 (2018).

325. Marzuoli R, Monga R, Finco A, Gerosa G. Biomass and physiological responses of *Quercus*

robur (L.) young trees during 2 years of treatments with different levels of ozone and nitrogen wet deposition. *Trees* **30**, 1995-2010 (2016).

326. Matyssek R, Gunthardtgoerg MS, Keller T, Scheidegger C. Impairment of gas-exchange and structure in birch leaves (*Betula pendula*) caused by low ozone concentrations. *Trees* **5**, 5-13 (1991).

327. McBranch NA*, et al.* Lack of acclimation of leaf area : sapwood area ratios in piñon pine and juniper in response to precipitation reduction and warming. *Tree Physiology* **00**, 1-8 (2018).

328. McElrone AJ, Reid CD, Hoye KA, Hart E, Jackson RB. Elevated CO_2 reduces disease incidence and severity of a red maple fungal pathogen via changes in host physiology and leaf chemistry. *Global Change Biology* **11**, 1828-1836 (2005).

329. Meng F*, et al.* Effects of elevated CO_2 and different irrigation on photosynthetic parameters and yield of maize in northeast China. *Acta Ecologica Sinica* **35**, 2126-2135 (2015).

330. Mikkelsen TN. Physiological-responses of *Fagus sylvatica* L. exposed to low-levels of ozone in open-top chambers. *Trees* **9**, 355-361 (1995).

331. Mikkelsen TN, Ropoulsen HR. Exposure of Norway spruce to ozone increases the sensitivity of current year needles to photoinhibition and desiccation. *New Phytologist* **128**, 153-163 (1994).

332. Momen B, Anderson PD, Helms JA, Houpis JLJ. Acid rain and ozone effects on gas exchange of *Pinus ponderosa*: A comparison between trees and seedlings. *International Journal of Plant Sciences* **158**, 617-621 (1997).

333. Moraes RM*, et al.* Physiological responses of saplings of *Caesalpinia echinata* Lam. a Brazilian tree species, under ozone fumigation. *Ecotoxicology and Environmental Safety* **63**, 306-312 (2006).

334. Morais MC*, et al.* Combined soil and foliar nitrogen fertilization effects on rainfed almond tree performance. *J Soil Sci Plant Nut* **20**, 2552-2565 (2020).

335. Mozdzer TJ, Caplan JS. Complementary responses of morphology and physiology enhance the stand-scale production of a model invasive species under elevated CO_2 and nitrogen. *Functional Ecology* **32**, 1784-1796 (2018).

336. Mu Z*, et al.* Seasonal and diurnal variations of plant isoprenoid emissions from two dominant species in Mediterranean shrubland and forest submitted to experimental drought. *Atmospheric Environment* **191**, 105-115 (2018).

337. Murthy R*, et al.* Increased leaf area dominates carbon flux response to elevated CO_2 in stands of *Populus deltoides* (Bartr.). *Global Change Biology* **11**, 716-731 (2005).

338. Murthy R, Zarnoch SJ, Dougherty PM. Seasonal trends of light-saturated net photosynthesis and stomatal conductance of loblolly pine trees grown in contrasting environments of nutrition, water and carbon dioxide. *Plant, Cell and Environment* **20**, 558-568 (1997).

339. Nackley LL*, et al.* CO_2 enrichment does not entirely ameliorate *Vachellia karroo* drought inhibition: A missing mechanism explaining savanna bush encroachment. *Environmental and Experimental Botany* **155**, 98-106 (2018).

340. Naidoo G. Differential effects of nitrogen and phosphorus enrichment on growth of dwarf *Avicennia marina* mangroves. *Aquatic Botany* **90**, 184-190 (2009).

341. Nakaji T, Fukami M, Dokiya Y, Izuta T. Effects of high nitrogen load on growth, photosynthesis and nutrient status of *Cryptomeria japonica* and *Pinus densiflora* seedlings. *Trees* **15**, 453-461 (2001).

342. Naumburg E, Ellsworth DS. Photosynthetic sunfleck utilization potential of understory saplings growing under elevated CO_2 in FACE. *Oecologia* **122**, 163-174 (2000).

343. Niinemets U*, et al.* Interactive effects of nitrogen and phosphorus on the acclimation potential of foliage photosynthetic properties of cork oak, *Quercus suber*, to elevated atmospheric CO_2 concentrations. *Global Change Biology* **5**, 455-470 (1999).

344. Nikula S, Percy K, Oksanen E, Holopainen T, Manninen S. Effects of elevated ozone on growth and foliar traits of European and hybrid aspen. *Boreal Environment Research* **14**, 29-47 (2009).

345. Niu J, Feng Z, Zhang W, Zhao P, Wang X. Non-stomatal limitation to photosynthesis in *Cinnamomum camphora* seedings exposed to elevated O_3. *Plos One* **9**, (2014).

346. Niu S, Wu M, Han Y, Xia J, Li L, Wan S. Water-mediated responses of ecosystem carbon fluxes to climatic change in a temperate steppe. *New Phytologist* **177**, 209-219 (2008).

347. Nogués I*, et al.* Physiological and antioxidant responses of *Quercus ilex* to drought in two different seasons. *Plant Biosystems* **148**, 268-278 (2014).

348. Nogues I, Medori M, Fortunati A, Lellei-Kovács E, Krel-Dulay G, Calfapietra C. Leaf gas exchange and isoprene emission in poplar in response to long-term experimental night-time warming and summer drought in a forest-steppe ecosystem. *Environmental and Experimental Botany* **152**, 60-67 (2018).

349. Noia Junior RdS, Macedo Pezzopane JE, Vinco JS, Teixeira Xavier TM, Cecilio RA, Macedo Pezzopane JR. Characterization of photosynthesis and transpiration in two rubber tree clones exposed to thermal stress. *Brazilian Journal of Botany* **41**, 785-794 (2018).

350. Noormets A*, et al.* Stomatal and non-stomatal limitation to photosynthesis in two trembling aspen (*Populus tremuloides* Michx.) clones exposed to elevated CO_2 and/or O_3. *Plant, Cell and Environment* **24**, 327-336 (2001).

351. Norby RJ, Oneill EG. Leaf area compensation and nutrient interactions in CO_2-enriched seedlings of yellow-poplar (*Liriodendron tulipifera* L.). *New Phytologist* **117**, 515-528 (1991).

352. Novak K*, et al.* Seasonal trends in reduced leaf gas exchange and ozone-induced foliar injury in three ozone sensitive woody plant species. *Environmental Pollution* **136**, 33-45 (2005).

353. Novriyanti E, Mao Q, Agathokleous E, Watanabe M, Hashidoko Y, Koike T. Elevated CO_2 offsets the alteration of foliar chemicals (*n*-icosane, geranyl acetate, and elixene) induced by elevated O_3 in three taxa of O_3-tolerant *Eucalypts*. *Journal of Forestry Research* **32**, 789-803 (2020).

354. Nowak RS*, et al.* Leaf conductance decreased under free-air CO_2 enrichment (FACE) for three perennials in the Nevada desert. *New Phytologist* **150**, 449-458 (2001).

355. O'Keefe K, Del Cid C, Pinedo CA, Puetz W, Springer CJ. Elevated CO_2 does not ameliorate the negative consequences of infection with the xylem-limited bacteria xylella fastidiosa in *Quercus rubra* seedlings. *Castanea* **78**, 216-226 (2013).

356. Oksanen E, Freiwald V, Prozherina N, Rousi M. Photosynthesis of birch (*Betula pendula*) is sensitive to springtime frost and ozone. *Canadian Journal of Forest Research* **35**, 703-712 (2005).

357. Oksanen E, Saleem A. Ozone exposure results in various carry-over effects and prolonged reduction in biomass in birch (*Betula pendula* Roth). *Plant, Cell and Environment* **22**, 1401-1411 (1999).

358. Onoda Y, Hirose T, Hikosaka K. Effect of elevated CO_2 levels on leaf starch, nitrogen and photosynthesis of plants growing at three natural CO_2 springs in Japan. *Ecological Research* **22**, 475-484 (2007).

359. Osório ML, Osório J, Romano A. Photosynthesis, energy partitioning, and metabolic adjustments of the endangered Cistaceae species *Tuberaria major* under high temperature and drought. *Photosynthetica* **51**, 75-84 (2013).

360. Paakkonen E, Gunthardt-Goerg MS, Holopainen T. Responses of leaf processes in a sensitive birch (*Betula pendula* Roth) clone to ozone combined with drought. *Annals of Botany* **82**, 49-59 (1998).

361. Paakkonen E*, et al.* Induction of genes for the stress proteins PR-10 and PAL in relation to growth, visible injuries and stomatal conductance in birch (*Betula pendula*) clones exposed to ozone and/or drought. *New Phytologist* **138**, 295-305 (1998).

362. Paakkonen E, Vahala J, Pohjolai M, Holopainen T, Karenlampi L. Physiological, stomatal and ultrastructural ozone responses in birch (*Betula pendula* Roth.) are modified by water stress. *Plant, Cell and Environment* **21**, 671-684 (1998).

363. Padu E, Kollist H, Tulva I, Oksanen E, Moldau H. Components of apoplastic ascorbate use in *Betula pendula* leaves exposed to CO_2 and O_3 enrichment. *New Phytologist* **165**, 131-141 (2005).

364. Palanisamy K. Interactions of elevated CO_2 concentration and drought stress on photosynthesis in *Eucalyptus cladocalyx* F. Muell. *Photosynthetica* **36**, 635-638 (1999).

365. Palmroth S, Holm Bach L, Nordin A, Palmqvist K. Nitrogen addition effects on leaf traits

and photosynthetic carbon gain of boreal forest understory shrubs. *Oecologia* **175**, 457-470 (2014).

366. Panigrahi S, Pradhan MK, Panda DK, Panda SK, Joshi PN. Diminution of photosynthesis in rice (*Oryza sativa* L.) seedlings under elevated CO_2 concentration and increased temperature. *Photosynthetica* **54**, 359-366 (2016).

367. Paoletti E, Nali C, Lorenzini G. Photosynthetic behavior of two Italian clones of European beech (*Fagus sylvatica* L.) exposed to ozone. *Phyton-Annales Rei Botanicae* **42**, 149-155 (2002).

368. Parvin S, Uddin S, Tausz-Posch S, Fitzgerald G, Armstrong R, Tausz M. Elevated CO_2 improves yield and N_2 fixation but not grain N concentration of faba bean (*Vicia faba* L.) subjected to terminal drought. *Environmental and Experimental Botany* **165**, 161-173 (2019).

369. Pasquini SC, Santiago LS. Nutrients limit photosynthesis in seedlings of a lowland tropical forest tree species. *Oecologia* **168**, 311-319 (2012).

370. Pastore MA, Lee TD, Hobbie SE, Reich PB. Interactive effects of elevated CO_2, warming, reduced rainfall, and nitrogen on leaf gas exchange in five perennial grassland species. *Plant, Cell and Environment* **43**, 1862-1878 (2020).

371. Pataki DE*, et al.* Water use of two Mojave desert shrubs under elevated CO_2. *Global Change Biology* **6**, 889-897 (2000).

372. Paudel I*, et al.* Elevated CO_2 compensates for drought effects in lemon saplings via stomatal downregulation, increased soil moisture, and increased wood carbon storage. *Environmental and Experimental Botany* **148**, 117-127 (2018).

373. Pearson M, Davies WJ, Mansfield TA. Asymmetric responses of adaxial and abaxial stomata to elevated CO_2: impacts on the control of gas exchange by leaves. *Plant, Cell and Environment* **18**, 837-843 (1995).

374. Pell EJ, Eckardt N, Enyedi AJ. Timing of ozone stress and resulting status of ribulose bisphosphate carboxylase oxygenase and associated net photosynthesis. *New Phytologist* **120**, 397-405 (1992).

375. Peng B*, et al.* Effects of ozone stress on photosynthesis and dry matter production of rice II-you 084 under different planting densities. *Chinese Journal of Applied Ecology* **26**, 17-24 (2015).

376. Peng B*, et al.* Effects of ozone stress on photosynthesis, dry matter production and yield of rice under different seedling quality and plant density. *Acta Ecologica Sinica* **33**, 3668-3675 (2013).

377. Perdomo JA, Carmo-Silva E, Hermida-Carrera C, Flexas J, Galmés J. Acclimation of biochemical and diffusive components of photosynthesis in rice, wheat, and maize to heat and water deficit: Implications for modeling photosynthesis. *Frontiers in Plant Science* **7**, 1719-1734 (2016).

378. Pérez-Soba M, Dueck TA, Puppi G, Kuiper PJC. Interactions of elevated CO_2, NH_3 and O_3 on mycorrhizal infection, gas exchange and N metabolism in saplings of Scots pine. *Plant and Soil* **176**, 107-116 (1995).

379. Phillips NG, Attard RD, Ghannoum O, Lewis JD, Logan BA, Tissue DT. Impact of variable CO_2 and temperature on water transport structure-function relationships in *Eucalyptus*. *Tree Physiology* **31**, 945-952 (2011).

380. Picon C, Ferhi A, Guehl JM. Concentration and delta ^{13}C of leaf carbohydrates in relation to gas exchange in *Quercus robur* under elevated CO_2 and drought. *Journal of Experimental Botany* **48**, 1547-1556 (1997).

381. Picon C, Guehl JM, Aussenac G. Growth dynamics, transpiration and water-use efficiency in *Quercus robur* plants submitted to elevated CO_2 and drought. *Annales Des Sciences Forestieres* **53**, 431-446 (1996).

382. Picon C, Guehl JM, Ferhi A. Leaf gas exchange and carbon isotope composition responses to drought in a drought-avoiding (*Pinus pinaster*) and a drought-tolerant (*Quercus petraea*) species under present and elevated atmospheric CO_2 concentrations. *Plant, Cell and Environment* **19**, 182-190 (1996).

383. Pivovaroff AL, Santiago LS, Vourlitis GL, Grantz DA, Allen MF. Plant hydraulic responses to long-term dry season nitrogen deposition alter drought tolerance in a Mediterranean-type ecosystem. *Oecologia* **181**, 721-731 (2016).

384. Polle A, McKee I, Blaschke L. Altered physiological and growth responses to elevated CO_2 in offspring from holm oak (*Quercus ilex* L.) mother trees with lifetime exposure to naturally elevated CO_2. *Plant, Cell and Environment* **24**, 1075-1083 (2001).

385. Polley HW, Johnson HB, Mayeux HS. Leaf physiology, production, water use, and nitrogen dynamics of the grassland invader *Acacia smallii* at elevated CO_2 concentrations. *Tree Physiology* **17**, 89-96 (1997).

386. Poole I, Lawson T, Weyers JDB, Raven JA. Effect of elevated CO_2 on the stomatal distribution and leaf physiology of *Alnus glutinosa*. *New Phytologist* **145**, 511-521 (2000).

387. Pozo DA, Pérez P, Morcuende R, Alonso A, Martínez-Carrasco R. Acclimatory responses of stomatal conductance and photosynthesis to elevated CO_2 and temperature in wheat crops grown at varying levels of N supply in a Mediterranean environment. *Plant Science* **169**, 908-916 (2005).

388. Prasad PVV, Djanaguiraman M. High night temperature decreases leaf photosynthesis and pollen function in grain sorghum. *Functional Plant Biology* **38**, 993-1003 (2011).

389. Prieto P, Penuelas J, Llusia J, Asensio D, Estiarte M. Effects of long-term experimental night-time warming and drought on photosynthesis, Fv/Fm and stomatal conductance in the dominant species of a Mediterranean shrubland. *Acta Physiologiae Plantarum* **31**, 729-739 (2009).

390. Qi H, Wang D, Qi M, Liu Y, He Y, Li T. Regulation of different calcium forms on the photosynthesis of tomato leaves under heat stress. *Chinese Journal of Applied Ecology* **25**, 3540-3546 (2014).

391. Quentin AG, Crous KY, Barton CVM, Ellsworth DS. Photosynthetic enhancement by elevated CO_2 depends on seasonal temperatures for warmed and non-warmed *Eucalyptus globulus* trees. *Tree Physiology* **35**, 1249-1263 (2015).

392. Rebbeck J, Jensen KF, Greenwood MS. Ozone effects on grafted mature and juvenile red spruce photosynthesis: stomatal conductance, and chlorophyll concentration. *Canadian Journal of Forest Research* **23**, 450-456 (1993).

393. Rebbeck J, Loats KV. Ozone effects on seedling sugar maple (*Acer saccharum*) and yellow-poplar (*Liriodendron tulipifera*): gas exchange. *Canadian Journal of Forest Research* **27**, 1595-1605 (1997).

394. Rebbeck J, Scherzer AJ, Loats KV. Foliar physiology of yellow-poplar (*Liriodendron tulipifera* L.) exposed to O_3 and elevated CO_2 over five seasons. *Trees* **18**, 253-263 (2004).

395. Reekie EG, Bazzaz FA. Competition and patterns of resource use among seedlings of five tropical trees grown at ambient and elevated CO_2. *Oecologia* **79**, 212-222 (1989).

396. Reinhardt K, McAbee K, Germino MJ. Changes in structure and physiological functioning due to experimentally enhanced precipitation seasonality in a widespread shrub species. *Plant Ecology* **220**, 199-211 (2019).

397. Ren J, Wang H, Wang W, Qu D, Wang Q, Zhong Z. Responses of photosynthesis, chlorophyll fluorescence of poplar leaf and bark chlorenchyma to elevated temperature. *Bulletin of Botanical Research* **34**, 758-764 (2014).

398. Ren Y, Wu B, Lu Q, He J, Liu M, Yao B. Responses of stomatal traits of desert plant *Niraria tangutorum* leaves to artificially simulated precipitation. *Forest Reaserch* **28**, 865-870 (2015).

399. Rey A, Jarvis PG. Long-term photosynthetic acclimation to increased atmospheric CO_2 concentration in young birch (*Betula pendula*) trees. *Tree Physiology* **18**, 441-450 (1998).

400. Ribas A, Penuelas J, Elvira S, Gimeno BS. Contrasting effects of ozone under different water supplies in two Mediterranean tree species. *Atmospheric Environment* **39**, 685-693 (2005).

401. Richet N*, et al.* Elevated CO_2 and or ozone modify lignification in the wood of poplars (*Populus tremula* x *alba*). *Journal of Experimental Botany* **63**, 4291-4301 (2012).

402. Riikonen J*, et al.* Needle metabolome, freezing tolerance and gas exchange in Norway spruce seedlings exposed to elevated temperature and ozone concentration. *Tree Physiology* **32**, 1102-1112 (2012).

403. Ripullone F*, et al.* Physiological and structural changes in response to altered precipitation regimes in a Mediterranean macchia ecosystem. *Trees* **23**, 823-834 (2009).

404. Roberntz P, Stockfors J. Effects of elevated CO_2 concentration and nutrition on net photosynthesis, stomatal conductance and needle respiration of field grown Norway spruce trees. *Tree Physiology* **18**, 233-241 (1998).

405. Robredo A*, et al.* Elevated CO_2 alleviates the impact of drought on barley improving water status by lowering stomatal conductance and delaying its effects on photosynthesis. *Environmental and Experimental Botany* **59**, 252-263 (2007).

406. Rodrigues WP*, et al.* Long-term elevated air [CO_2] strengthens photosynthetic functioning and mitigates the impact of supra-optimal temperatures in tropical *Coffea arabica* and *C. canephora* species. *Global Change Biology* **22**, 415-431 (2016).

407. Rogers A*, et al.* Leaf photosynthesis and carbohydrate dynamics of soybeans grown throughout their life-cycle under free-air carbon dioxide enrichment. *Plant, Cell and Environment* **27**, 449-458 (2004).

408. Rosa BL, Souza JP, Pereira EG. Increased atmospheric CO_2 changes the photosynthetic responses of *Acrocomia aculeata* (*Arecaceae*) to drought. *Acta Botanica Brasilica* **33**, 486-497 (2019).

409. Roumet C, Garnier E, Suzor H, Salager JL, Roy J. Short and long term responses of whole plant gas exchange to elevated CO_2 in four herbaceous species. *Environmental and Experimental Botany* **43**, 155-169 (2000).

410. Rowland L*, et al.* Plant traits controlling growth change in response to a drier climate. *New Phytologist* **229**, 1363-1374 (2020).

411.Ruehr NK, Martin JG, Law BE. Effects of water availability on carbon and water exchange in a young ponderosa pine forest: above- and belowground responses. *Agricultural and Forest Meteorology* **164**, 136-148 (2012).

412. Ruhil K, Sheeba, Ahmad A, Iqbal M, Tripathy BC. Photosynthesis and growth responses of mustard (*Brassica juncea* L. cv pusa bold) plants to free air carbon dioxide enrichment (FACE). *Protoplasma* **252**, 935-946 (2015).

413. Ruiz-Vera UM*, et al.* Global warming can negate the expected CO_2 stimulation in photosynthesis and productivity for soybean grown in the midwestern united states. *Plant Physiology* **162**, 410-423 (2013).

414. Ruiz-Vera UM, Siebers MH, Drag DW, Ort DR, Bernacchi CJ. Canopy warming caused photosynthetic acclimation and reduced seed yield in maize grown at ambient and elevated [CO_2]. *Global Change Biology* **21**, 4237-4249 (2015).

415. Sage E, Heisler-White J, Morgan J, Pendall E, Williams DG. Climate warming alters photosynthetic responses to elevated CO_2 in prairie plants. *American Journal of Botany* **107**, 1238-1252 (2020).

416. Salazar-Parra C*, et al.* Carbon balance, partitioning and photosynthetic acclimation in

fruit-bearing grapevine (*Vitis vinifera* L. cv. tempranillo) grown under simulated climate change (elevated CO_2, elevated temperature and moderate drought) scenarios in temperature gradient greenhouses. *J Plant Physiol* **174**, 97-109 (2015).

417. Samuelson LJ. The role of microclimate in determining the sensitivity of *Quercus rubra* L. to ozone. *New Phytologist* **128**, 235-241 (1994).

418. Samuelson LJ*, et al.* Fertilization increased leaf water use efficiency and growth of *Pinus taeda* subjected to five years of throughfall reduction. *Canadian Journal of Forest Research* **48**, 227-236 (2018).

419. Samuelson LJ*, et al.* Two-year throughfall and fertilization effects on leaf physiology and growth of loblolly pine in the Georgia Piedmont. *Forest Ecology and Management* **330**, 29-37 (2014).

420. Sanches RFE, Centeno DD, Braga MR, da Silva EA. Impact of high atmospheric CO_2 concentrations on the seasonality of water-related processes, gas exchange, and carbohydrate metabolism in coffee trees under field conditions. *Climatic Change* **162**, 1231-1248 (2020).

421. Schaub M*, et al.* Physiological and foliar injury responses of *Prunus serotina*, *Fraxinus americana*, and *Acer rubrum* seedlings to varying soil moisture and ozone. *Environmental Pollution* **124**, 307-320 (2003).

422. Schmid I*, et al.* Effects of CO_2 enrichment and drought on photosynthesis, growth and yield of an old and a modern barley cultivar. *Journal of Agronomy and Crop Science* **202**, 81-95 (2016).

423. Schoenbeck L*, et al.* Soil nutrients and lowered source:sink ratio mitigate effects of mild but not of extreme drought in trees. *Environmental and Experimental Botany* **169**, (2020).

424. Sebastiani L, Minnocci A, Tognetti R. Genotypic differences in the response to elevated CO_2 concentration of one-year-old olive cuttings (*Olea europaea* L. cv. frantoio and moraiolo). *Plant Biosystems* **136**, 199-207 (2002).

425. Semedo JN*, et al.* Intrinsic non-stomatal resilience to drought of the photosynthetic apparatus in *Coffea* spp. is strengthened by elevated air [CO_2]. *Tree Physiology* **41**, 708-727 (2021).

426. Shao Z*, et al.* Impact of elevated atmospheric carbon dioxide and ozone concentrations on leaf photosynthesis of 'Shanyou 63' hybrid rice. *Chinese Journal of Eco-agriculture* **22**, 422-429 (2014).

427. Shehab AESAE, Guo Y. Effects of nitrogen fertilization and drought on hydrocyanic acid accumulation and morpho-physiological parameters of sorghums. *Journal of the Science of Food and Agriculture* **101**, 3355-3365 (2021).

428. Shi B, Wang Y, Meng B, Zhong S, Sun W. Effects of nitrogen addition on the drought susceptibility of the *Leymus chinensis* meadow ecosystem vary with drought duration. *Frontiers*

in Plant Science **9**, (2018).

429. Shimono H, Okada M, Inoue M, Nakamura H, Kobayashi K, Hasegawa T. Diurnal and seasonal variations in stomatal conductance of rice at elevated atmospheric CO_2 under fully open-air conditions. *Plant, Cell and Environment* **33**, 322-331 (2010).

430. Sicher R. Combined effects of CO_2 enrichment and elevated growth temperatures on metabolites in soybean leaflets: evidence for dynamic changes of TCA cycle intermediates. *Planta* **238**, 369-380 (2013).

431. Signarbieux C, Feller U. Effects of an extended drought period on physiological properties of grassland species in the field. *Journal of Plant Research* **125**, 251-261 (2012).

432. Sigurdsson BD*, et al.* Impact studies on Nordic forests: effects of elevated CO_2 and fertilization on gas exchange. *Canadian Journal of Forest Research* **32**, 779-788 (2002).

433. Singsaas EL, Ort DR, DeLucia EH. Diurnal regulation of photosynthesis in understory saplings. *New Phytologist* **145**, 39-49 (2000).

434. Smith NG, Pold G, Goranson C, Dukes JS. Characterizing the drivers of seedling leaf gas exchange responses to warming and altered precipitation: indirect and direct effects. *Aob Plants* **8**, (2016).

435. Smith RA, Lewis JD, Ghannoum O, Tissue DT. Leaf structural responses to pre-industrial, current and elevated atmospheric CO_2 and temperature affect leaf function in *Eucalyptus sideroxylon*. *Functional Plant Biology* **39**, 285-296 (2012).

436. Soldatini GF, Lorenzini G, Filippi F, Nali C, Guidi L. Photosynthesis of two poplar clones under long-term exposure to ozone. *Physiologia Plantarum* **104**, 707-712 (1998).

437. Song M*, et al.* Nitrogen-controlled intra- and interspecific competition between *Populus purdomii* and *Salix rehderiana* drive primary succession in the Gongga mountain glacier retreat area. *Tree Physiology* **37**, 799-814 (2017).

438. Song Y, Chen Q, Ci D, Shao X, Zhang D. Effects of high temperature on photosynthesis and related gene expression in poplar. *BMC Plant Biology* **14**, 111-130 (2014).

439. Sonjaroon W, Kaveeta L, Chai-arree W, Klinsakorn S, Suksamrarn A, Jutamanee K. Exogenous 7,8-dihydro-8α-20-hydroxyecdysone application improves antioxidative enzyme system, photosynthesis, and yield in rice under high temperature condition. *Acta Physiologiae Plantarum* **38**, 202-212 (2016).

440. Su B*, et al.* Photosynthesis and biochemical responses to elevated O_3 in *Plantago major* and *Sonchus oleraceus* growing in a lowland habitat of northern China. *Journal of Environmental Sciences* **53**, 113-121 (2017).

441. Sugai T, Watanabe T, Kita K, Koike T. Nitrogen loading increases the ozone sensitivity of larch seedlings with higher sensitivity to nitrogen loading. *Science of The Total Environment* **663**, 587-595 (2019).

442. Sun J, Wu J, Ren L, Zhang G, Ren F, Yao F. Response of photosynthetic physiological characteristics to nitrogen addition by seedlings of two dominant tree species in a broadleaved-Korean pine mixed forest on Changbai mountain. *Acta Ecologica Sinica* **36**, 6777-6785 (2016).

443. Sun J, Yao F, Wu J, Zhang P, Xu W. Effect of nitrogen levels on photosynthetic parameters, morphological and chemical characters of saplings and trees in a temperate forest. *Journal of Forestry Research* **29**, 1481-1488 (2018).

444. Sun S, Wang Q, Sun C, Liu F, Bi H, Ai X. Response and adaptation of photosynthesis of cucumber seedlings to high temperature stress. *Chinese Journal of Applied Ecology* **28**, 1603-1610 (2017).

445. Takemoto BK, Bytnerowicz A, Dawson PJ, Morrison CL, Temple PJ. Effects of ozone on *Pinus ponderosa* seedlings: comparison of responses in the first and second growing seasons of exposure. *Canadian Journal of Forest Research* **27**, 23-30 (1997).

446. Talhelm AF, Pregitzer KS, Burton AJ. No evidence that chronic nitrogen additions increase photosynthesis in mature sugar maple forests. *Ecological Applications* **21**, 2413-2424 (2011).

447. Tang B, Yin C, Yang H, Sun Y, Liu Q. The coupling effects of water deficit and nitrogen supply on photosynthesis, WUE, and stable isotope composition in *Picea asperata*. *Acta Physiologiae Plantarum* **39**, (2017).

448. Teng Z, Zhi L, Jun L, Zong X, Wang S, He G. Effects of high temperature on photosynthesis characteristics, phytohormones and grain quality during filling-periods in rice. *Acta Ecologica Sinica* **30**, 6504-6511 (2010).

449. Tezara W, Mitchell V, Driscoll SP, Lawlor DW. Effects of water deficit and its interaction with CO_2 supply on the biochemistry and physiology of photosynthesis in sunflower. *Journal of Experimental Botany* **53**, 1781-1791 (2002).

450. Thiec DL, Dixon G, Garrec JP. The effects of slightly elevated ozone concentrations and mild drought stress on the physiology and growth of Norway spruce, *Picea abies* (L.) Karst. and beech, *Fagus sylvatica* L., in open-top chambers. *New Phytologist* **128**, 671-678 (2010).

451. Thiec LD, Dixon M. Acclimation of photosynthesis in Norway spruce and red oak grown in open-top chambers and subjected to natural drought and to elevated CO_2. *Canadian Journal of Forest Research* **26**, 87-94 (1996).

452. Thiec LD, Dixon M, Garrec JP. The effects of slightly elevated ozone concentrations and mild drought stress on the physiology and growth of Norway spruce, *Picea abies* (L.) Karst and beech, *Fagus sylvatica* L., in open-top chambers. *New Phytologist* **128**, 671-678 (1994).

453. Thiec LD, Manninen S. Ozone and water deficit reduced growth of *Aleppo* pine seedlings. *Plant Physiology and Biochemistry* **41**, 55-63 (2003).

454. Throop HL, Reichmann LG, Sala OE, Archer SR. Response of dominant grass and shrub

species to water manipulation: an ecophysiological basis for shrub invasion in a Chihuahuan desert grassland. *Oecologia* **169**, 373-383 (2012).

455. Thruppoyil SB, Ksiksi T. Time dependent stomatal conductance and growth responses of *Tabernaemontana divaricata* to short term elevated CO_2 and water stress at higher than optimal growing temperature. *Current Plant Biology* **22**, 100127 (2020).

456. Tissue DT, Thomas RB, Strain BR. Atmospheric CO_2 enrichment increases growth and photosynthesis of *Pinus taeda*: a 4 year experiment in the field. *Plant, Cell and Environment* **20**, 1123-1134 (1997).

457. Tjoelker MG, Luxmoore RJ. Soil nitrogen and chronic ozone stress influence physiology, growth and nutrient status of *Pinus taeda* L. and *Liriodendron tulipifera* L. seedlings. *New Phytologist* **119**, 69-81 (1991).

458. Tjoelker MG, Oleksyn J, Reich PB. Seedlings of five boreal tree species differ in acclimation of net photosynthesis to elevated CO_2 and temperature. *Tree Physiology* **18**, 715-726 (1998).

459. Tjoelker MG, Volin JC, Oleksyn J, Reich PB. Light environment alters response to ozone stress in seedlings of *Acer saccharum* Marsh and hybrid *Populus* L. *New Phytologist* **124**, 627-636 (1993).

460. Tjoelker MG, Volin JC, Oleksyn J, Reich PB. Interaction of ozone pollution and light effects on photosynthesis in a forest canopy experiment. *Plant, Cell and Environment* **18**, 895-905 (1995).

461. Tognetti R, Johnson JD. The effect of elevated atmospheric CO_2 concentration and nutrient supply on gas exchange, carbohydrates and foliar phenolic concentration in live oak (*Quercus virginiana* Mill.) seedlings. *Annals of Forest Science* **56**, 379-389 (1999).

462. Tognetti R, Johnson JD. Responses to elevated atmospheric CO_2 concentration and nitrogen supply of *Quercus ilex* L. seedlings from a coppice stand growing at a natural CO_2 spring. *Annals of Forest Science* **56**, 549-561 (1999).

463. Tognetti R, Longobucco A, Raschi A, Miglietta F, Fumagalli I. Responses of two *Populus* clones to elevated atmospheric CO_2 concentration in the field. *Annals of Forest Science* **56**, 493-500 (1999).

464. Tognetti R, Sebastiani L, Vitagliano C, Raschi A, Minnocci A. Responses of two olive tree (*Olea europaea* L.) cultivars to elevated CO_2 concentration in the field. *Photosynthetica* **39**, 403-410 (2001).

465. Tom-Dery D, Eller F, Fromm J, Jensen K, Reisdorff C. Elevated CO_2 does not offset effects of competition and drought on growth of shea (*Vitellaria paradoxa*) seedlings. *Agroforestry Systems* **93**, 1807-1819 (2019).

466. Torres-Ruiz JM*, et al.* Shoot hydraulic characteristics, plant water status and stomatal

response in olive trees under different soil water conditions. *Plant and Soil* **373**, 77-87 (2013).

467. Triboulot MB, Fauveau ML, Breda N, Label P, Dreyer E. Stomatal conductance and xylem-sap abscisic acid (ABA) in adult oak trees during a gradually imposed drought. *Annales Des Sciences Forestieres* **53**, 207-220 (1996).

468. Tricker PJ*, et al.* Stomatal conductance and not stomatal density determines the long-term reduction in leaf transpiration of poplar in elevated CO_2. *Oecologia* **143**, 652-660 (2005).

469. Tschaplinski BTJ, Stewart DB, Hanson PJ, Norby RJ. Interactions between drought and elevated CO_2 on growth and gas exchange of seedlings of three deciduous tree species. *New Phytologist* **129**, 63-71 (1995).

470. Turner CL, Knapp AK. Responses of a C_4 grass and three C_3 forbs to variation in nitrogen and light in *Tallgrass Prairie*. *Ecology* **77**, 1738-1749 (1996).

471. Uddling J, Hogg AJ, Teclaw RM, Carroll MA, Ellsworth DS. Stomatal uptake of O_3 in aspen and aspen-birch forests under free-air CO_2 and O_3 enrichment. *Environmental Pollution* **158**, 2023-2031 (2010).

472. Uddling J, Teclaw RM, Pregitzer KS, Ellsworth DS. Leaf and canopy conductance in aspen and aspen-birch forests under free-air enrichment of carbon dioxide and ozone. *Tree Physiology* **29**, 1367-1380 (2009).

473. Uddling J, Wallin G. Interacting effects of elevated CO_2 and weather variability on photosynthesis of mature boreal Norway spruce agree with biochemical model predictions. *Tree Physiology* **32**, 1509-1521 (2012).

474. Ultra VU, Jr., Han S-H, Kim D-H, Aggangan NS. Changes in growth, photosynthetic parameters, carbon and nitrogen uptake of *Pinus densiflora* under elevated temperature and CO_2 level. *Asia Life Sciences* **21**, 523-536 (2012).

475. Urban L, Barthelemy L, Bearez P, Pyrrha P. Effect of elevated CO_2 on photosynthesis and chlorophyll fluorescence of rose plants grown at high temperature and high photosynthetic photon flux density. *Photosynthetica* **39**, 275-281 (2001).

476. Urban O*, et al.* Interactive effects of ultraviolet radiation and elevated CO_2 concentration on photosynthetic characteristics of European beech saplings during the vegetation season. *Plant Physiology and Biochemistry* **134**, 20-30 (2019).

477. Urban O*, et al.* Impact of elevated CO_2 concentration on dynamics of leaf photosynthesis in *Fagus sylvatica* is modulated by sky conditions. *Environmental Pollution* **185**, 271-280 (2014).

478. Verdaguer D, Diaz-Guerra L, Font J, Gonzalez JA, Llorens L. Contrasting seasonal morphological and physio-biochemical responses to UV radiation and reduced rainfall of two mature naturally growing Mediterranean shrubs in the context of climate change. *Environmental and Experimental Botany* **147**, 189-201 (2018).

479. Volin JC, Reich PB. Interaction of elevated CO_2 and O_3 on growth, photosynthesis and

respiration of three perennial species grown in low and high nitrogen. *Physiologia Plantarum* **97**, 674-684 (1996).

480. Volin JC, Reich PB, Givnish TJ. Elevated carbon dioxide ameliorates the effects of ozone on photosynthesis and growth: species respond similarly regardless of photosynthetic pathway or plant functional group. *New Phytologist* **138**, 315-325 (1998).

481. Volin JC, Tjoelker MG, Oleksyn J, Reich PB. Light environment alters response to ozone stress in seedlings of *Acer saccharum* Marsh and hybrid *Populus* L. *New Phytologist* **124**, 637-646 (1993).

482. Vu JCV. Acclimation of peanut (*Arachis hypogaea* L.) leaf photosynthesis to elevated growth CO_2 and temperature. *Environmental and Experimental Botany* **53**, 85-95 (2005).

483. Vu JCV, Allen LH, Jr. Growth at elevated CO_2 delays the adverse effects of drought stress on leaf photosynthesis of the C_4 sugarcane. *J Plant Physiol* **166**, 107-116 (2009).

484. Vu JCV, Newman YC, Allen LH, Gallo-Meagher M, Zhang MQ. Photosynthetic acclimation of young sweet orange trees to elevated growth CO_2 and temperature. *J Plant Physiol* **159**, 147-157 (2002).

485. Wall GW, *et al.* Acclimation response of spring wheat in a free-air CO_2 enrichment (FACE) atmosphere with variable soil nitrogen regimes. 2. Net assimilation and stomatal conductance of leaves. *Photosynthesis Research* **66**, 79-95 (2000).

486. Wall GW, *et al.* Elevated atmospheric CO_2 improved sorghum plant water status by ameliorating the adverse effects of drought. *New Phytologist* **152**, 231-248 (2001).

487. Wall GW, *et al.* Interactive effects of elevated carbon dioxide and drought on wheat. *Agronomy Journal* **98**, 354-381 (2006).

488. Wall GW, Garcia RL, Wechsung F, Kimball BA. Elevated atmospheric CO_2 and drought effects on leaf gas exchange properties of barley. *Agriculture Ecosystems and Environment* **144**, 390-404 (2011).

489. Wallin G, Skarby L. The influence of ozone on the stomatal and nonstomatal limitation of photosynthesis in Norway spruce, *Picea abies* (L.) Karst, exposed to soil moisture deficit. *Trees* **6**, 128-136 (1992).

490. Wallin G, Skarby L, Sellden G. Long-term exposure of Norway spruce, *Picea abies* (L.) Karst, to ozone in open-top chambers. *New Phytologist* **115**, 335-344 (1990).

491. Walter A, Christ MM, Barron-gafford GA, Grieve KA, Murthy R, Rascher U. The effect of elevated CO_2 on diel leaf growth cycle, leaf carbohydrate content and canopy growth performance of *Populus deltoides*. *Global Change Biology* **11**, 1207-1219 (2005).

492. Wand SJE, Midgley GF, Musil CF. Physiological and growth responses of two African species, *Acacia karroo* and *Themeda triandra*, to combined increases in CO_2 and UV-B radiation. *Physiologia Plantarum* **98**, 882-890 (1996).

493. Wang A*, et al.* Responses of hydraulics at the whole-plant level to simulated nitrogen deposition of different levels in *Fraxinus mandshurica*. *Tree Physiology* **36**, 1045-1055 (2016).

494. Wang B*, et al.* Leaf photosynthesis and stomatal conductance acclimate to elevated CO_2 and temperature thus increasing dry matter productivity in a double rice cropping system. *Field Crops Research* **248**, (2020).

495. Wang C*, et al.* Effects of elevated atmospheric CO_2 concentration on soybean photosynthesis and chlorophyll fluorescence parameters. *Journal of Nuclear Agricultural Sciences* **29**, 1583-1588 (2015).

496. Wang D, Heckathorn SA, Mainali K, Hamilton EW. Effects of N on plant response to heat-wave: a field study with prairie vegetation. *Journal of Integrative Plant Biology* **50**, 1416-1425 (2008).

497. Wang D*, et al.* Impact of nitrogen allocation on growth and photosynthesis of Miscanthus (*Miscanthus × giganteus*). *GCB Bioenergy* **4**, 688-697 (2012).

498. Wang H, Zhou G, Jiang Y, Shi H, Xu Z. Interactive effects of changing precipitation and elevated CO_2 concentration on photosynthetic parameters of *Stipa breviflora*. *Chinese Journal of Ecology* **36**, 597-606 (2012).

499. Wang J, Li Y, Huang S, Liu L, Ye Q. Effects of elevated CO2 concentration on photosynthetic rate, growth, and development in *Anthurium andraeanum* L. leaves. *Acta Horticulturae Sinica* **32**, 335-335 (2005).

500. Wang K, Kellomaki S. Stomatal conductance and transpiration in shoots of Scots pine after 4-year exposure to elevated CO_2 and temperature. *Canadian Journal of Botany* **75**, 552-561 (1997).

501. Wang L, Li Y, Li X, Ma L, He X. Co-ordination of photosynthesis and stomatal responses of mongolian oak (*Quercus mongolica*) to elevated O_3 and/or CO_2 levels. *Applied Ecology and Environmental Research* **17**, 4257-4268 (2019).

502. Wang M, Shi S, Lin F, Hao Z, Jiang P, Dai G. Effects of soil water and nitrogen on growth and photosynthetic response of Manchurian Ash (*Fraxinus mandshurica*) seedlings in Northeastern China. *Plos One* **7**, e30754 (2012).

503. Wang M*, et al.* Effects of different elevated CO_2 concentrations on chlorophyll contents, gas exchange, water use efficiency, and PSII activity on C_3 and C_4 cereal crops in a closed artificial ecosystem. *Photosynthesis Research* **126**, 351-362 (2015).

504. Wang N, Zhang Y, Qian W, Wang Z, Gu J. Effects of elevated CO_2 concentration on root and needle anatomy and physiological functions in *Pinus koraiensis* seedlings. *Chinese Journal of Plant Ecology* **40**, 60-68 (2016).

505. Wang R*, et al.* Growth, gas exchange, root morphology and cadmium uptake responses of poplars and willows grown on cadmium-contaminated soil to elevated CO_2. *Environmental Earth*

Sciences **67**, 1-13 (2012).

506. Wang R, Xiong X. Effects of temperature stress on growth and metabolism in perennial ryegrass. *Acta Prataculturae Sinica* **25**, 81-90 (2016).

507. Wang X, Curtis PS. Gender specific responses of *Populus tremuloides* to atmospheric CO_2 enrichment. *New Phytologist* **150**, 675-684 (2001).

508. Wang X, Curtis PS, Pregitzer KS, Zak DR. Genotypic variation in physiological and growth responses of *Populus tremuloides* to elevated atmospheric CO_2 concentration. *Tree Physiology* **20**, 1019-1028 (2000).

509. Warren CR, Loew M, Matyssek R, Tausz M. Internal conductance to CO_2 transfer of adult *Fagus sylvatica*: variation between sun and shade leaves and due to free-air ozone fumigation. *Environmental and Experimental Botany* **59**, 130-138 (2007).

510. Watanabe M, Kinose Y, Izuta T. Photosynthesis of three evergreen broad-leaved tree species, *Castanopsis sieboldii*, *Quercus glauca*, and *Q. myrsinaefolia*, under elevated ozone. *Iforest-Biogeosciences and Forestry* **11**, 360-366 (2018).

511. Watanabe M*, et al.* Photosynthetic traits and growth of *Quercus mongolica* var. *crispula* sprouts attacked by powdery mildew under free-air CO_2 enrichment. *European Journal of Forest Research* **133**, 725-733 (2014).

512. Watanabe M*, et al.* Photosynthetic traits of Siebold's beech seedlings in changing light conditions by removal of shading trees under elevated CO_2. *Plant Biology* **18**, 56-62 (2016).

513. Watanabe M, Ryu K, Kita K, Takagi K, Koike T. Effect of nitrogen load on growth and photosynthesis of seedlings of the hybrid larch F 1 (*Larix gmelinii* var. *japonica*× *L. kaempferi*) grown on serpentine soil. *Environmental and experimental botany* **83**, 73-81 (2012).

514. Watanabe M*, et al.* Influences of nitrogen load on the growth and photosynthetic responses of *Quercus serrata* seedlings to O_3. *Trees* **21**, 421-432 (2007).

515. Watanabe Y, Satomura T, Sasa K, Funada R, Koike T. Differential anatomical responses to elevated CO_2 in saplings of four hardwood species. *Plant, Cell and Environment* **33**, 1101-1111 (2010).

516. Watanabe Y*, et al.* Effects of elevated CO_2 and nitrogen on wood structure related to water transport in seedlings of two deciduous broad-leaved tree species. *Trees* **22**, 403-411 (2008).

517. Watanabe Y*, et al.* Response of tree growth and wood structure of *Larix kaempferi*, *Kalopanax septemlobus* and *Betula platyphylla* saplings to elevated CO_2 concentration for 5 years exposure in a FACE system. *Trees* **30**, 1569-1579 (2016).

518. Weber JA, Clark CS, Hogsett WE. Analysis of the relationships among O_3 uptake, conductance, and photosynthesis in needles of *Pinus ponderosa*. *Tree Physiology* **13**, 157-172 (1993).

519. Wechsung F, *et al.* Photosynthesis and conductance of spring wheat ears: field response to free-air CO_2 enrichment and limitations in water and nitrogen supply. *Plant, Cell and Environment* **23**, 917-929 (2000).

520. Wedler M, Weikert RM, Lippert M. Photosynthetic performance, chloroplast pigments and mineral content of Norway spruce (*Picea abies* (L.) Karst) exposed to SO_2 and O_3 in an open-air fumigation experiment. *Plant, Cell and Environment* **18**, 263-276 (1995).

521. Wei Z, Du T, Li X, Fang L, Liu F. Interactive effects of CO_2 concentration elevation and nitrogen fertilization on water and nitrogen use efficiency of tomato grown under reduced irrigation regimes. *Agricultural Water Management* **202**, 174-182 (2018).

522. Wertin TM, McGuire MA, Teskey RO. The influence of elevated temperature, elevated atmospheric CO_2 concentration and water stress on net photosynthesis of loblolly pine (*Pinus taeda* L.) at northern, central and southern sites in its native range. *Global Change Biology* **16**, 2089-2103 (2010).

523. Wieser G, Havranek WM. Exposure of mature norway spruce to ozone in twig-chambers effects on gas-exchange. *Proceedings of the Royal Society of Edinburgh* **102**, 119-125 (1994).

524. Will RE, Ceulemans R. Effects of elevated CO_2 concentration on photosynthesis, respiration and carbohydrate status of coppice *Populus* hybrids. *Physiologia Plantarum* **100**, 933-939 (1997).

525. Wolfe-Bellin KS, He J, Bazzaz FA. Leaf-level physiology, biomass, and reproduction of *Phytolacca americana* under conditions of elevated carbon dioxide and increased nocturnal temperature. *International Journal of Plant Sciences* **167**, 1011-1020 (2006).

526. Wu FZ, Bao WK, Li FL, Wu N. Effects of water stress and nitrogen supply on leaf gas exchange and fluorescence parameters of *Sophora davidii* seedlings. *Photosynthetica* **46**, 40-48 (2008).

527. Wu G, *et al.* Differential responses of stomata and photosynthesis to elevated temperature in two co-occurring subtropical forest tree species. *Frontiers in Plant Science* **9**, (2018).

528. Wujeska-Klause A, Bossinger G, Tausz M. Responses to heatwaves of gas exchange, chlorophyll fluorescence and antioxidants ascorbic acid and glutathione in congeneric pairs of *Acacia* and *Eucalyptus* species from relatively cooler and warmer climates. *Trees* **29**, 1929-1941 (2015).

529. Wullschleger SD, Gunderson CA, Hanson PJ, Wilson KB, Norby RJ. Sensitivity of stomatal and canopy conductance to elevated CO_2 concentration – interacting variables and perspectives of scale. *New Phytologist* **153**, 485-496 (2002).

530. Wullschleger SD, Hanson PJ, Edwards GS. Growth and maintenance respiration in leaves of northern red oak seedlings and mature trees after 3 years of ozone exposure. *Plant, Cell and Environment* **19**, 577-584 (1996).

531. Wullschleger SD, Norby RJ, Hendrix DL. Carbon exchange rates, chlorophyll content, and carbohydrate status of two forest tree species exposed to carbon dioxide enrichment. *Tree Physiology* **10**, 21-31 (1992).

532. Xiao S, Dong Y, Qi Y, Peng Q, He Y, Liu X. Effects of mineral fertilizer addition on leaf functional traits and photosynthetic characteristics of *Leymus chinensis* from a temperate grassland in Inner Mongolia in China. *Acta Scientiae Circumstantiae* **30**, 2535-2543 (2010).

533. Xin Y, Shang B, Chen X, Feng Z. Effects of elevated ozone and nitrogen deposition on photosynthetic characteristics and biomass of *Populus cathayana*. *Environmental Science* **37**, 3642-3649 (2016).

534. Xiong D*, et al.* Effects of elevated O_3 concentration on photosynthetic physiological characteristics of *Catalpa ovata*. *Chinese Journal of Ecology* **36**, 944-950 (2017).

535. Xu G, Singh SK, Reddy VR, Barnaby JY, Sicher RC, Li T. Soybean grown under elevated CO_2 benefits more under low temperature than high temperature stress: varying response of photosynthetic limitations, leaf metabolites, growth, and seed yield. *J Plant Physiol* **205**, 20-32 (2016).

536. Xu Q, Henry RL, Guikema JA, Paulsen GM. Association of high-temperature injury with increased sensitivity of photosynthesis to abscisic acid in wheat. *Environemntal and Experimental Botany* **35**, 441-454 (1995).

537. Xu S, Chen W, Huang Y, He X. Responses of growth, photosynthesis and voc emissions of *Pinus tabulaeformis* carr. exposure to elevated CO_2 and/or elevated O_3 in an urban area. *Bulletin of Environmental Contamination and Toxicology* **88**, 443-448 (2012).

538. Xu W*, et al.* Effects of elevated ozone concentration and nitrogen addition on ammonia stomatal compensation point in a poplar clone. *Environmental Pollution* **238**, 760-770 (2018).

539. Xu Y, Feng Z, Shang B, Yuan X, Tarvainen L. Limited water availability did not protect poplar saplings from water use efficiency reduction under elevated ozone. *Forest Ecology and Management* **462**, (2020).

540. Xu Z, Zhou G, Shimizu H. Effects of soil drought with nocturnal warming on leaf stomatal traits and mesophyll cell ultrastructure of a perennial grass. *Crop Science* **49**, 1843-1851 (2009).

541. Xu ZZ, Zhou GS. Combined effects of water stress and high temperature on photosynthesis, nitrogen metabolism and lipid peroxidation of a perennial grass *Leymus chinensis*. *Planta* **224**, 1080-1090 (2006).

542. Yamaguchi M*, et al.* Growth and photosynthetic responses of *Fagus crenata* seedlings to O_3 under different nitrogen loads. *Trees* **21**, 707-718 (2007).

543. Yamaguchi M*, et al.* Effects of sulfur dioxide on growth and net photosynthesis of six Japanese forest tree species grown under different nitrogen loads. *Trees* **26**, 1859-1874 (2012).

544. Yamaji K, Julkunen-Tiitto R, Rousi M, Freiwald V, Oksanen E. Ozone exposure over two

growing seasons alters root-to-shoot ratio and chemical composition of birch (*Betula pendula* Roth). *Global Change Biology* **9**, 1363-1377 (2010).

545. Yan H, Wu Q, Ding J, Zhang S. Effects of precipitation and nitrogen addition on photosynthetically eco-physiological characteristics and biomass of four tree seedlings in Gutian Mountain Zhejiang Province, China. *Acta Ecologica Sinica* **33**, 4226-4236 (2013).

546. Yan K, Chen W, He X, Zhang G, Xu S, Wang L. Responses of photosynthesis, lipid peroxidation and antioxidant system in leaves of *Quercus mongolica* to elevated O_3. *Environmental and Experimental Botany* **69**, 198-204 (2010).

547. Yan K*, et al.* Elevated CO_2 ameliorated oxidative stress induced by elevated O_3 in *Quercus mongolica*. *Acta Physiologiae Plantarum* **32**, 375-385 (2010).

548. Yang H, Luo Y. Responses of the functional traits in *Cleistogenes squarrosa* to nitrogen addition and drought. *Chinese Journal of Plant Ecology* **39**, 32-42 (2015).

549. Yang K, Meng F, Guo X. Effects of CO_2 fertilization on photosynthesis and growth of cut *Anthurium andraeanum* in solar greenhouse in winter. *Chinese Journal of Applied Ecology* **28**, 1941-1947 (2017).

550. Yang Q, Zhao W, Liu B, Liu H. Physiological responses of *Haloxylon ammodendron* to rainfall pulses in temperate desert regions, Northwestern China. *Trees* **28**, 709-722 (2014).

551. Yang S, Yang Z, Wang L, Li J, Zhang M, Li K. Effect of high humidity and high temperature interaction on photosynthetic characteristics of greenhouse tomato crops. *Chinese Journal of Ecology* **37**, 57-63 (2018).

552. Yang W, Huang F, Cao Z, Lei B, Hu D, Cheng F. Effects of high temperature stress on PSII function and its relation to D1 protein in chloroplast thylakoid in rice flag leaves. *Acta Agronomica Sinica* **39**, 1060-1068 (2013).

553. Yang Y, Zong J, Wang J, Liu P, Yang F. Effects of high temperature stress on physiological indexes of *Eucommia ulmoides* seedlings. *Journal of Northeast Forestry University* **43**, 14-17 (2015).

554. Yao F, Wang X, Feng Z, Zheng F, Feng Z, Ouyang Z. Influence of ozone and ethylenediurea (EDU) on physiological characters and foliar symptom of spinach (*Spinacia oleracea* L.) in open-top chambers. *Ecology and Environment* **16**, 1399-1405 (2007).

555. Yao X, Liu Q. Changes in morphological, photosynthetic and physiological responses of Mono Maple seedlings to enhanced UV-B and to nitrogen addition. *Plant Growth Regulation* **50**, 165-178 (2006).

556. Yao X, Liu Q. Photosynthetic and physiological responses of *Swida hemsleyi* subjected to enhanced UV-B enhanced and to nitrogen suppy. *Polish Journal of Ecology* **57**, 483-494 (2009).

557. Yao X, Liu Q. The effects of enhanced ultraviolet-B and nitrogen supply on growth, photosynthesis and nutrient status of *Abies faxoniana* seedlings. *Acta Physiologiae Plantarum* **31**,

523-529 (2009).

558. Yao X, Liu Q, Han C. Growth and photosynthetic responses of *Picea asperata* seedlings to enhanced ultraviolet-B and to nitrogen supply. *Brazilian Journal of Plant Physiology* **20**, 11-18 (2008).

559. Yeboah S*, et al.* Soil water content and photosynthetic capacity of spring wheat as affected by soil application of nitrogen-enriched biochar in a semiarid environment. *Photosynthetica* **55**, 532-542 (2017).

560. Yendrek CR*, et al.* Elevated ozone reduces photosynthetic carbon gain by accelerating leaf senescence of inbred and hybrid maize in a genotype-specific manner. *Plant, Cell and Environment* **40**, 3088-3100 (2017).

561. Yin C, Palmroth S, Pang X, Tang B, Liu Q, Oren R. Differential responses of *Picea asperata* and *Betula albosinensis* to nitrogen supply imposed by water availability. *Tree Physiology* **38**, 1694-1705 (2018).

562. Yu F, Xu X, Robert DG. Effects of water plus heat stress on gas exchange and water use efficiency in four species of coniferous seedlings. *Sciential Silvae Sinicae* **40**, 38-44 (2004).

563. Yu H, Chen Z, Cao J, Shang H. Effects of elevated O_3 level on photosynthesis, antioxidant capacity and biomass of *Phoebe zhennan* and *Phoebe bournei* seedlings in subtropical china. *Forest Research* **31**, 33-41 (2018).

564. Yu H*, et al.* Short term nitrogen addition does not significantly alter the effects of seasonal drought on leaf functional traits in *Machilus pauhoi* Kanehira seedlings. *Forests* **10**, 78-97 (2019).

565. Yu H, Shang H, Chen Z, Cao J. Effects of elevated O_3 level on photosynthesis and injury of *Phoebe* and *Machilus* seedlings in subtropical china. *Forest Research* **29**, 902-910 (2016).

566. Yu K*, et al.* Photosynthetic physiological resposne character of different maturity maize leaves to heat stress and their genotype difference. *Journal of Henan Agricultural Sciences* **46**, 34-38 (2017).

567. Yu L, Dong TF, Lu YB, Song MY, Duan BL. Ecophysiological responses of *Cunninghamia lanceolata* to nongrowing-season warming, nitrogen deposition, and their combination. *Photosynthetica* **54**, 598-610 (2016).

568. Yu L, Liu N, Zhao Q, Wei R, Guo W. Effects of different ozone concentrations on growth and development of eggplants. *Northern Horticulture* **19**, 1-5 (2017).

569. Yu X, Zhang X, Guo T, Yu J. Effects of nitrogen fertilization on wheat leaf photosynthesis under elevated atmospheric CO_2 concentration. *Chinese Journal of Applied Ecology* **21**, 2342-2346 (2010).

570. Yuan X*, et al.* No significant interactions between nitrogen stimulation and ozone inhibition of isoprene emission in *Cathay poplar*. *Science of The Total Environment* **601**, 222-229 (2017).

571. Yun S, Laurence JA. The response of clones of *Populus tremuloides* differing in sensitivity to ozone in the field. *New Phytologist* **141**, 411-421 (1999).

572. Zhan W, Sha W, Wang M, Shi S, Wen Q. Effects of the variations in precipitation and temperature on photosynthetic parameters and growth of *Fraxinus mandshurica* seedlings in Changbai Mountains of Northeast China. *Chinese Journal of Applied Ecology* **23**, 617-624 (2012).

573. Zhang B*, et al.* Asymmetric sensitivity of ecosystem carbon and water processes in response to precipitation change in a semi-arid steppe. *Functional Ecology* **31**, 1301-1311 (2017).

574. Zhang C, Guo J, Chen G, Hengcai X. E ffects of high temperature and /or drought on grow th and secondary metabo lites in *Ginkgo biloba* leaves. *Journal of Ecology and Rural Environment* **21**, 11-15 (2005).

575. Zhang G*, et al.* Effects of high temperature on physiological and biochemical characteristics in flag leaf of rice during heading and flowering period. *Scientia Agricultura Sinica* **40**, 1345-1352 (2007).

576. Zhang H*, et al.* Responses of functional traits to seven-year nitrogen addition in two tree species: coordination of hydraulics, gas exchange and carbon reserves. *Tree Physiology* **41**, 190-205 (2021).

577. Zhang J, Ferdinand JA, Vanderheyden DJ, Skelly JM, lnnes JL. Variation of gas exchange within native plant species of Switzerland and relationships with ozone injury: an open-top experiment. *Environmental Pollution* **113**, 177-185 (2001).

578. Zhang L, Hoshika Y, Carrari E, Cotrozzi L, Pellegrini E, Paoletti E. Effects of nitrogen and phosphorus imbalance on photosynthetic traits of poplar Oxford clone under ozone pollution. *Journal of Plant Research* **131**, 915-924 (2018).

579. Zhang L, Su B, Xu H, Li Y. Growth and photosynthetic responses of four landscape shrub species to elevated ozone. *Photosynthetica* **50**, 67-76 (2012).

580. Zhang L, Xu H, J. Y, Li W, Jiang G, Li Y. Photosynthetic characteristics of diploid honeysuckle (*Lonicera japonica* Thunb.) and its autotetraploid cultivar subjected to elevated ozone exposure. *Photosynthetica* **48**, 87-95 (2010).

581. Zhang N, Huang W, Hu T, Lio X, Yin L, Liu W. Effects of high temperature stress on physiological characteristic of seedlings leafin *Simmondsia chinensis*. *Journal of Sichuan Agricultural University* **29**, 311-316 (2011).

582. Zhang P, Huang Z, Zhang W, Wang Q, Liu M. Effect of high temperature on photosynthetic performance in wheat. *Journal of Anhui Agricultural University* **42**, 92-96 (2015).

583. Zhang Q, Zhang Y, Peng S, Zobel K. Climate warming may facilitate invasion of the exotic shrub *Lantana camara*. *Plos One* **9**, (2014).

584. Zhang Q, Zong Y, Dong Q, Hu X, Hao X. Effects of elevated atomosphere CO_2

concentration on soybean photosynthesis. *Journal of Shanxi Agricultural Sciences* **44**, 1675-1679 (2016).

585. Zhang S, Dang Q. Effects of soil temperature and elevated atmospheric CO_2 concentration on gas exchange, in vivo carboxylation and chlorophyll fluorescence in jack pine and white birch seedlings. *Tree Physiology* **25**, 523-531 (2005).

586. Zhang T*, et al.* Leaf hydraulic acclimation to nitrogen addition of two dominant tree species in a subtropical forest. *Science of The Total Environment* **771**, 145415 (2021).

587. Zhang T, Wang L, Yang J. Effect of elevated CO_2 concentration on photosynthetic efficiency soybean under drought s tress. *Journal of Henan Agricultural Sciences* **34**, 47-50 (2005).

588. Zhang W, Feng Z, Wang X, Niu J. Responses of native broadleaved woody species to elevated ozone in subtropical China. *Environmental Pollution* **163**, 149-157 (2012).

589. Zhang W, Feng Z, Wang X, Niu J. Impacts of elevated ozone on growth and photosynthesis of *Metasequoia glyptostroboides*. *Plant Science* **226**, 182-188 (2014).

590. Zhang W, Niu J, Feng Z, Wang X, Tian Y, Yao F. Responses of *Ilex integra* Thunb. seedlings to elevated air ozone concentration. *Environmental Science* **32**, 2414-2421 (2011).

591. Zhang W, Wang G, Wang M, Liu X, Feng Z. Responses of soybean cultivar Dongsheng-1 to different O_3 concentrations in northeast china. *Environmental Science* **35**, 1473-1478 (2014).

592. Zhang W, Wang M, Wang A, Yin X, Feng Z, Hao G. Elevated ozone concentration decreases whole plant hydraulic conductance and disturbs water use regulation in soybean plants. *Physiologia Plantarum* **163**, 183-195 (2018).

593. Zhang W*, et al.* Effects of simulated nitrogen deposition on photosynthetic characteristics of the invasive plant *Mikania micrantha*. *Ecology and Environmental Sciences* **22**, 1859-1866 (2013).

594. Zhang X, Chen L, Wang J, Wang M, Yang S, Zhao C. Photosynthetic acclimation to long term high temperature and soil drought stress in two spruce species (*Picea crassifolia* and *P. wilsonii*) used for afforestation. *Journal of Forestry Research* **29**, 363-372 (2018).

595. Zhang X, Yu X, Hou H, Wang H, Ma Y. Effect of nitrogen and shade on photosynthetic gas exchange and productivity of wheat under elevated atmospheric CO_2 concentration. *Proceedings of the National Academy of Sciences* **87**, 965-974 (2015).

596. Zhang X, Yu X, Ma Y. Effect of nitrogen application and elevated CO_2 on photosynthetic gas exchange and electron transport in wheat leaves. *Photosynthetica* **51**, 593-602 (2013).

597. Zhang Y, Duan B, Qiao Y, Wang K, Korpelainen H, Li C. Leaf photosynthesis of *Betula albosinensis* seedlings as affected by elevated CO_2 and planting density. *Forest Ecology and Management* **255**, 1937-1944 (2008).

598. Zhang Y, Li X, Zhang F, Liao Y. Effects of elevated CO_2 on physiological characteristics of water in *Pinus koraiensis* seedlings and soil moisture in a controlled environment. *Journal of Beijing Forestry University* **37**, 37-41 (2015).

599. Zhang Y, Yu X, Chen L, Jia G. Whole plant instantaneous and short-term water-use efficiency in response to soil water content and CO_2 concentration. *Plant and Soil* **444**, 281-298 (2019).

600. Zhang Y, Zhang Y, Liu X, Zhu J. Effects of elevated temperature and drought on photosynthetic characteristics and antioxidant enzyme system of *Poplar* seedlings *Journal of Northeast Forestry University* **45**, 32-38 (2017).

601. Zhang Z, Li L, Jian C, Chen S, Li K. Photosynthetic characteristic of temperature response for *Cassava* seeding leaf. *Chinese Journal of Tropical Crops* **36**, 103-109 (2015).

602. Zhao C, Liu Q. Growth and photosynthetic responses of two coniferous species to experimental warming and nitrogen fertilization. *Canadian Journal of Forest Research* **39**, 1-11 (2008).

603. Zhao H, Xu X, Zhang Y, Korpelainen H, Li C. Nitrogen deposition limits photosynthetic response to elevated CO_2 differentially in a dioecious species. *Oecologia* **165**, 41-54 (2011).

604. Zhao L, Deng R, Xiang Q, Xia L, Zhao L. Effects of high temperature stress on gas exchange and chlorophyll fluorescence parameters of *Fagopyrum dibotrys*. *Partacultural Science* **32**, 560-569 (2015).

605. Zhao S, Yu J, Xiao G. Effects of high temperature stress on the photosynthesis and osmoregulation substances of flag leaves in *Oryza stavia* L. ssp. *javanica*. *Ecology and Environmental Sciences* **22**, 110-115 (2013).

606. Zhao Y, Xu S, He X, Chen W. Physiological response of two *Lolium* varieties to the enhancements of tropospheric O_3 concentration. *Acta Agrestia Sinica* **23**, 1013-1020 (2015).

607. Zheng Y, Hu C, Wu R, Liu R, Zhao Z, Zhang J. Effects of ozone stress upon winter wheat photosynthesis, lipid peroxidation and antioxidant systems. *Environmental Science* **31**, 1643-1651 (2010).

608. Zheng Y, Liu R, Wu R, Zhao Z, Hu C. Effects of ozone stress on diurnal change of winter wheat photosynthesis at milking stage. *Chinese Journal of Ecology* **30**, 1075-1082 (2011).

609. Zheng Y, Xu M, Hou R, Shen R, Qiu S, Ouyang Z. Effects of experimental warming on stomatal traits in leaves of maize (*Zea may* L.). *Ecology and Evolution* **3**, 3095-3111 (2013).

610. Zhou N*, et al.* Effects of elevated atmospheric CO_2 and temperature on diurnal courses of photosynthesis in leaves of *Japonica* rice. *Chinese Journal of Ecology* **35**, 2404-2416 (2016).

611. Zhou Y*, et al.* Ten-year exposure to elevated CO_2 increases stomatal number of *Pinus koraiensis* and *P. sylvestriformis* needles. *European Journal of Forest Research* **132**, 899-908 (2013).

612. Zhu F, Lu X, Mo J. Phosphorus limitation on photosynthesis of two dominant understory species in a lowland tropical forest. *Journal of Plant Ecology* **7**, 526-534 (2014).

613. Zhu L, Li X, Jia X, Wang B, Jin S. High temperature stress with photosynthesis in *Festuca arundinacea*. *Journal of Zhejiang Forestry College* **26**, 652-655 (2009).

614. Zhu X, Song F, Liu S, Liu T. Effects of arbuscular mycorrhizal fungus on photosynthesis and water status of maize under high temperature stress. *Plant Soil* **346**, 189-199 (2011).

615. Zhu Y*, et al.* Effects of high temperature on leaf stomatal traits and gas exchange parameters of blueberry. *Transactions of the Chinese Society of Agricultural Engineering* **32**, 218-225 (2016).

616. Zhuang M, Chen S, Li Y, Guo Z, Yang Q, Deng Z. Changes of physiological responses of *Phyllostachys edulis* to atomosphere ozone stress. *Acta Botanical Boreali-Occidentalia Sinica* **33**, 132-138 (2013).
